# Supplementary material for: Sexual activity and contraceptive use among adolescents: A descriptive survey in a Ghanaian municipality
Source: PLOS Glob Public Health. 2025 Aug 25;5(8):e0005039. doi: 10.1371/journal.pgph.0005039 (PMC12377570; doi:10.1371/journal.pgph.0005039)
Supplement: S1 Text — (DOCX) [file pgph.0005039.s001.docx]

**QUESTIONNAIRE**

Dear Respondent,

This is a data collection tool (questionnaire) on a study titled “***Sexual activity and contraceptive use among adolescence; a study in senior high schools in Ejisu Municipality”.***

Thank you for volunteering to complete this questionnaire. Your response is very important. The purpose of this questionnaire is purely academic; therefore, all your responses will be treated with strict confidentiality and will in no way be linked to you.

Thank You.

**INSTRUCTIONS:**

- Where alternatives have been provided, tick (✓) your answer in the spaces provided.
- For the other questions without alternatives at all, kindly write your answers in the spaces provided.
- For section Five, indicate your level of agreement to each statement using a five-point Likert Scale of Strongly disagree (SD), Disagree (D), Uncertain (U), Agree (A) and strongly agree (SA)

**SECTION A: SOCIO-DEMOGRAPHIC INFORMATION OF RESPONDENTS**

| **Information** | **Response** |
| --- | --- |
| 1. Age range (Years) | ☐10-14  ☐ 15-19 |
| 1. Gender/Sex | ☐Male  ☐Female |
| 1. Class | ☐S.H. S 1  ☐S.H. S 2  ☐S.H. S 3 |
| 1. Religion | ☐Muslim  ☐Christian  ☐Traditionalist  ☐If other specify……………………… |
| 1. Ethnicity | ☐Akan  ☐Fante  ☐Ga-Adangbe  ☐ Ewe  ☐Others,  specify……………………… |
| 1. Whom do you stay with? | ☐Both parents  ☐Single condition  ☐Live alone |
| 1. Employment status of the person you live with | ☐Unemployed  ☐ Employed |

**SECTION 2A: SEXUAL ACTIVITY AMONG ADOLECSCENTS**

| **Questions** | **Response** |
| --- | --- |
| 8.Do you have a boyfriend/girlfriend? | ☐Yes  ☐No |
| 9.If yes, what is the age of your boyfriend/girlfriend? | ☐10-19  ☐More than 19 |
| 10.Have you ever had sex before? | ☐Yes  ☐No |
| 11.How many sexual partners have you had in your lifetime? | ☐1  ☐2  ☐3  ☐More than 3 |
| 12. How old were you when you had your first sexual intercourse? | ☐10-14  ☐15-19 |
| 13.How did your first sexual intercourse happen? | ☐Own will  ☐Coaxed  ☐Forced |
| 14.Do you feel any pressure from others to have sexual intercourse? | ☐Yes  ☐No |
| 15.If yes, indicate the main group of persons from whom you were pressured. | ☐Friends  ☐Relatives  ☐Teachers  ☐Boyfriend/Girlfriend |

**SECTION THREE: AWARENESS OF CONTRACEPTIVES**

| **Questions** | **Response** |
| --- | --- |
| 16.Have you ever heard about contraception? | ☐Yes  ☐No |
| 17.Heard of any method of contraceptives | ☐Yes  ☐No |
| 18.Where did you hear it from? | ☐Radio  ☐Television  ☐Teacher  ☐Peers  ☐Parents  ☐Healthcare worker |
| 19.What method of contraceptive do you know | ☐Male condom  ☐Abstinence  ☐Implant  ☐Withdrawal method  ☐Female condom  ☐IUD  ☐Urinating after sex  ☐ Injectables  ☐Pills |
| 20.Heard about emergency contraceptives? | ☐Yes  ☐No |
| 21.If yes, where did you heard it from | ☐Radio  ☐Television  ☐Peers  ☐Health worker  ☐Teacher  ☐Internet  ☐Parents |
| 22.How often do you know emergency contraceptives can be used within a year? | ☐Once a year  ☐Twice a year  ☐Three times a year  ☐More than three times a year |
| 23.Do you know where one can access contraceptives? | ☐Yes  ☐No  ☐I don’t know |
| 24.If yes, Where? | ☐Pharmacy  ☐Friend  ☐Hospital/clinic  ☐Health personnel |
| 25.Can a girl become pregnant from just one sexual intercourse? | ☐Yes  ☐No  ☐I don’t know |
| 26.Does contraceptive provide 100% protection from pregnancy? | ☐Yes  ☐No  ☐I don’t know |

**SECTION FOUR: USE OF CONTRACEPTIVES AMONG ADOLESECENTS**

| **Questions** | **Response** |
| --- | --- |
| 27.Have you ever used contraceptive method? If no, skip to question | ☐Yes  ☐No |
| 28.What method did you use? ***(you can select more than one answer)*** | ☐Condom  ☐Injectables  ☐Pills  ☐Withdrawal  ☐IUD  ☐Safe period  ☐Implant  ☐Others,  Specify ……………… |
| 29.Do you use contraceptive any time you have sex? | ☐Yes  ☐No |
| 30.If yes, what is/are your reason(s) for using modern contraceptives? ***(you can select more than one answer)*** | ☐To avoid pregnancy  ☐To prevent STD |
| 31.Did you discuss about contraceptive method with your partner the first time you had sex? | ☐Yes  ☐No  ☐I do not remember |
| 32.Did you use contraceptive the first time you had sex? | ☐Yes  ☐No |
| 33.If yes, what method(s) did you or your partner used the first time you had sex? ***(you can select more than one answer)*** | ☐Condom  ☐Withdrawal  ☐Pills  ☐Safe period  ☐Implants  ☐injectables  ☐IUD  ☐Others,  Specify…………………... |
| 34.Where did you get access to this contraceptive(s)? ***(you can select more than one answer)*** | ☐Hospital  ☐Pharmacy  ☐Friends  ☐Health personnel  ☐Others,  Specify…………………. |
| 35.How often do you use contraceptive? | ☐Every time I have sex  ☐Only on my first sexual intercourse  ☐Once a while |
| 36.Have you ever used emergency contraceptive? (**females only)** | ☐Yes  ☐No |
| 37.If yes, how often do you use emergency contraceptive? ***(females only)*** | ☐Every time I have sex  ☐Once a while  ☐Twice a year  ☐Three times a year  ☐More than three times a year |

**SECTION FIVE: ATTITUDE OF ADOLESCENTS TOWARDS CONTRACEPTIVE.**

| **Questions** | **Response** |
| --- | --- |
| 38.I approve of the use of contraception by adolescents anytime they have sex | ☐Strongly disagree  ☐disagree  ☐Uncertain  ☐agree  ☐Strongly agree |
| 39.Use of contraceptive by a girl before her first birth can lead to infertility | ☐Strongly disagree  ☐disagree  ☐Uncertain  ☐agree  ☐Strongly agree |
| 40.Sex is not enjoyable when I use condom | ☐Strongly disagree  ☐disagree  ☐Uncertain  ☐agree  ☐Strongly agree |
| 41.People who insist on condom use are promiscuous (have multiple sexual partners) | ☐Strongly disagree  ☐disagree  ☐Uncertain  ☐agree  ☐Strongly agree |
| 42.Contraception usage is against my religion | ☐Strongly disagree  ☐disagree  ☐Uncertain  ☐agree  ☐Strongly agree |
| 43.I will use contraception in the future | ☐Strongly disagree  ☐disagree  ☐Uncertain  ☐agree  ☐Strongly agree |
| 44.Contraception is the responsibility of only females | ☐Strongly disagree  ☐disagree  ☐Uncertain  ☐agree  ☐Strongly agree |
