# Supplementary material for: Sexual activity and contraceptive use among adolescents: A descriptive survey in a Ghanaian municipality
Source: PLOS Glob Public Health. 2025 Aug 25;5(8):e0005039. doi: 10.1371/journal.pgph.0005039 (PMC12377570; doi:10.1371/journal.pgph.0005039)
Supplement: S1 Checklist — (DOCX) [file pgph.0005039.s002.docx]

STROBE Statement—checklist of items that should be included in reports of observational studies

|  | Item No. | Recommendation | Page  No. | Relevant text from manuscript |
| --- | --- | --- | --- | --- |
| **Title and abstract** | 1 | (*a*) Indicate the study’s design with a commonly used term in the title or the abstract | 2 | Sexual activity and contraceptive use among adolescents: A quantitative study |
|  |  | (*b*) Provide in the abstract an informative and balanced summary of what was done and what was found | 2-3 | A cross-sectional study was conducted among 330 Senior High School (SHS) adolescents using simple random sampling. Categorical data were analyzed using frequencies and percentages. Pearson’s chi-squared test and binary logistic regression respectively were employed to assess associations and predict the relationship between independent variables and contraceptive use.  Almost fifty-percent (50.3%) of the adolescents were in an intimate relationship. over one-third (38%) had ever had sexual intercourse and out of this more than half (51.6%) had early initiation of sexual intercourse between the ages of 10-14years. Among those who had ever had sex, approximately seventy-nine (79.4%) had ever used contraceptive. Adolescents who had boyfriend or girlfriend (AOR=5.604; 95%CL: 2.918-10.763), having multiple sexual partners (AOR=10.631; 95%CL: 5.016-22.532), and being aware of contraceptive (AOR=5.105; 95%CI: 1.533-16.999) had higher odds of contraceptive use than their counterparts. |
| Introduction | | | |  |
| Background/rationale | 2 | Explain the scientific background and rationale for the investigation being reported | 4-5 | Contraceptive usage is very substantive in preventing unwanted pregnancies, unsafe abortions, and abortion related complications that expose adolescents to health related risks such as infertility and sometimes death (17). Comparing to other regions like Europe, Latin America and the Caribbean, and Northern America contraceptive use in Middle and Western Africa is below 25 percent(18).  Many studies on adolescent sexual behavior in the Ghanaian context have predominantly focused on adolescent girls and young women (12,19–21). Additionally, studies that included all adolescents have primarily focused on those in late adolescence (17,22,23) failing to capture the full range of adolescent experiences. Notably, there is a lack of research specifically targeting adolescents in senior high schools. Contraceptive use in Ghana remains low, and the factors influencing contraceptive use among high school adolescents have not been adequately assessed. Therefore, this study aims to fill this gap by examining the sexual activity and contraceptive use among adolescents in senior high schools. Addressing this issue is crucial not only for improving adolescent health outcomes but also for advancing economic growth, innovation, and long-term national development. |
| Objectives | 3 | State specific objectives, including any prespecified hypotheses | 5 | The current study sought to sexual activity and contraceptive use among adolescents in a senior high school. |
| Methods | | | |  |
| Study design | 4 | Present key elements of study design early in the paper | 6 | The study employed quantitative study which used an analytical cross-sectional design, which involved collection of data using a structured questionnaire. |
| Setting | 5 | Describe the setting, locations, and relevant dates, including periods of recruitment, exposure, follow-up, and data collection | 6 | The study was conducted within the setting of a senior high school located at Ejisu municipality, Ghana, which constitutes both rural and urban localities(25). Senior high school is a second cycle institution after one graduate from Junior high school. This school was selected as the study setting due to its diverse student population and it caters for students from various economic backgrounds. The school is a public mixed school which operates day and boarding. The School Offers five (5) programmes which includes; Business, Home Economics, Visual Arts, General Arts and General Science. The average yearly enrollment is about 700. Facilities within the school include classrooms, administrative offices, a library, laboratories and outdoor spaces such as playgrounds and sports fields. |
| Participants | 6 | (*a*) *Cohort study*—Give the eligibility criteria, and the sources and methods of selection of participants. Describe methods of follow-up  *Case-control study*—Give the eligibility criteria, and the sources and methods of case ascertainment and control selection. Give the rationale for the choice of cases and controls  *Cross-sectional study*—Give the eligibility criteria, and the sources and methods of selection of participants | 6-7 | Participants who were eligible to be sampled for the study included those who: a) were between the ages of 10 to 19years, b) could speak, understand, or write either English. Sections of students who were on vacation, critically ill, or had mental impairments were excluded from the study.  Simple random sampling technique was used in selecting eligible participants for this study. Thus, all participants who met the inclusion criteria were asked to choose ‘’yes’’ or ‘’no’’ from an opaque sealed envelope. |
|  |  | (*b*) *Cohort study*—For matched studies, give matching criteria and number of exposed and unexposed  *Case-control study*—For matched studies, give matching criteria and the number of controls per case |  |  |
| Variables | 7 | Clearly define all outcomes, exposures, predictors, potential confounders, and effect modifiers. Give diagnostic criteria, if applicable | 10 | The independent variables were the sociodemographic variables (age, sex, class, residential status, religion, ethnicity and employment status of the person participant lives with), having boyfriend/girlfriend, number of sexual partners, and having heard of contraceptive.  The dependent variable was **Contraceptive use.** Participants were classified as ever used contraceptive when he or she indicated ‘’yes.’’ |
| Data sources/ measurement | 8* | For each variable of interest, give sources of data and details of methods of assessment (measurement). Describe comparability of assessment methods if there is more than one group | 7-8 | A structured questionnaire consisting of six sections was used for the survey. The first section had sociodemographic characteristics of adolescents (7 items): This section includes questions on age, gender, class, religion, ethnicity, residential status (i.e., whom the adolescent lives with), and the employment status of the person the adolescent stays with. Sexual activity (8 items): This section covers topics such as whether the respondent has a boyfriend or girlfriend, the age of their sexual partner, whether they have ever had sex, the number of sexual partners, the age of first sexual intercourse, how the first sexual experience occurred (whether they were coaxed, forced, or it was their own will), and whether they feel pressured to have sex. Awareness of contraceptives (11 items): Questions in this section include whether the respondent has heard of modern contraceptives, the source of this information, the methods of contraception they are aware of, where one can access contraceptives, as well as similar inquiries regarding emergency contraceptives. Contraceptive use (11 items): This section includes questions on whether the respondent has ever used contraceptives, which methods they have used, whether they use contraceptives every time they have sex, and the reasons for using them, such as “to avoid teenage pregnancy” or “to prevent STIs.” Other questions include whether they used contraceptives during their first sexual intercourse, where they accessed contraceptives, and how frequently they use them, with options like “every time I have sex,” “only during my first sexual intercourse,” or “once in a while.” Questions on the use of emergency contraceptives and their frequency of use were also included. Attitudes of adolescents towards contraceptives (7 Likert scale items): This section includes statements like “I approve of adolescents using contraceptives every time they have sex.” Higher scores reflect a positive attitude. Other statements, presented in a negative form and reverse-coded, include: “Using contraceptives before a girl’s first birth can lead to infertility,” “Sex is not enjoyable when I use a condom,” and “People who insist on condom use are promiscuous.” Barriers to contraceptive use among adolescents (8 items): Respondents were asked to indicate whether certain factors were barriers to their contraceptive use. These factors include religious beliefs, the attitude of providers, fear of side effects, embarrassment when buying contraceptives, fear of being seen by parents, partner disapproval, unavailability of contraceptives, and cultural beliefs. |
| Bias | 9 | Describe any efforts to address potential sources of bias | 9 | To reduce social desirability bias, participants were assured of confidentiality and anonymity, and teachers were asked to leave the classrooms during questionnaire administration. This created a more comfortable environment, encouraging participants to provide honest responses without fear of judgment. |
| Study size | 10 | Explain how the study size was arrived at | 7 | A sample size of 330 was determined using the Slovin’s formula:  $n=\frac{N}{1+N{(e)}^{2}}$ (26), and with a confidence interval of 95% (at a Z-score of 1.96), margin of error of 5%, where **n** signifies the sample size, **N** signifies the population under study and **e** signifies the margin of error (0.05). The total population was 1210 using the total enrollment of students at the time of the study obtained from the school’s administrative office. The minimum required sample size obtained was 300. To compensate for non-response, a 10% non-response rate was considered, resulting in a total estimated sample size of 330. |

Continued on next page

| Quantitative variables | 11 | Explain how quantitative variables were handled in the analyses. If applicable, describe which groupings were chosen and why | 9-10 | To identify the factors associated with contraceptive use, first chi-square test was done to assess the association between the independent variables and the dependent variable. The ones that were found to be significant were further analyzed using binary logistic regression. Multivariable logistic regression analyses were done and reported using odds ratio and their 95% confidence intervals. The significance level was set at a p-value of ≤ 0.05. |
| --- | --- | --- | --- | --- |
| Statistical methods | 12 | (*a*) Describe all statistical methods, including those used to control for confounding | 9 | Descriptive statistics such as frequencies and percentages were used to describe all categorical variables including age, sex, class, religion, ethnicity and among others. To identify the factors associated contraceptive use, a binary logistic regression analysis was done. Multivariable logistic regression analyses were done and reported using odds ratio and their 95% confidence intervals. The significance level was set at a p-value of ≤ 0.05. |
|  |  | (*b*) Describe any methods used to examine subgroups and interactions |  |  |
|  |  | (*c*) Explain how missing data were addressed | 14 | There were no missing data in the survey responses as the researchers thoroughly examined all sections to ensure their completeness. |
|  |  | (*d*) *Cohort study*—If applicable, explain how loss to follow-up was addressed  *Case-control study*—If applicable, explain how matching of cases and controls was addressed  *Cross-sectional study*—If applicable, describe analytical methods taking account of sampling strategy |  |  |
|  |  | (*e*) Describe any sensitivity analyses |  |  |
| Results | | | | |
| Participants | 13* | (a) Report numbers of individuals at each stage of study—eg numbers potentially eligible, examined for eligibility, confirmed eligible, included in the study, completing follow-up, and analysed | 6 | Between July and November 2022, a total of 330 Senior High School adolescents participated in the survey. |
|  |  | (b) Give reasons for non-participation at each stage |  |  |
|  |  | (c) Consider use of a flow diagram |  |  |
| Descriptive data | 14* | (a) Give characteristics of study participants (eg demographic, clinical, social) and information on exposures and potential confounders | 12-13 | Majority of the participants were in their late adolescents’ stage 290 (87.9%), and were females 196 (59.4%). Most of the participants lived with both parents 194 (58.8%), and were Christians 300 (90.9%). More than half 190 (57.6%) were in SHS 2, and were Akan’s 272 (82.4%). Majority of persons adolescent lives with were employed 201(60.9%). |
|  |  | (b) Indicate number of participants with missing data for each variable of interest |  |  |
|  |  | (c) *Cohort study*—Summarise follow-up time (eg, average and total amount) |  |  |
| Outcome data | 15* | *Cohort study*—Report numbers of outcome events or summary measures over time |  |  |
|  |  | *Case-control study—*Report numbers in each exposure category, or summary measures of exposure |  |  |
|  |  | *Cross-sectional study—*Report numbers of outcome events or summary measures | 14-18 | More than two-third 126(38.0%). of the participants had ever had sex, and out of this number approximately seventy-nine 100(79.4%) had ever used contraceptive. |
| Main results | 16 | (*a*) Give unadjusted estimates and, if applicable, confounder-adjusted estimates and their precision (eg, 95% confidence interval). Make clear which confounders were adjusted for and why they were included | 28-29 | In multivariable logistic regression, it was found that adolescents having boyfriend or girlfriend had 5 times higher odds to contraceptive use than those who are not in intimate relationship (AOR= 5.604, 95% CI: 2.918-10.763, p=0.006) (**Table 8). Also, adolescents who has multiple sexual partners had higher odds of using contraceptives** (AOR= 10.631, 95% CI: 5.016-22.532, p=0.001) and participants who had heard of contraceptive had 5 times higher odds to contraceptive use (AOR= 5.105, 95% CI: 1.533-16.999, p=0.016) (**Table 8).** |
|  |  | (*b*) Report category boundaries when continuous variables were categorized |  |  |
|  |  | (*c*) If relevant, consider translating estimates of relative risk into absolute risk for a meaningful time period |  |  |

Continued on next page

| Other analyses | 17 | Report other analyses done—eg analyses of subgroups and interactions, and sensitivity analyses |  |  |
| --- | --- | --- | --- | --- |
| Discussion | | | | |
| Key results | 18 | Summarise key results with reference to study objectives | 29-33 | From the study, it was revealed that notable number of the participants had ever had sex and out of that higher number engaged in sexual intercourse in the early stages of adolescents. Among those who had ever had sex, the majority had used contraceptives. This is further supported by the results of the multivariable logistic regression, which indicated that the likelihood of contraceptive use was higher among adolescents who had a boyfriend or girlfriend, multiple sexual partners, and had heard of contraceptives. A significant proportion of adolescents who had ever had sex also reported having a boyfriend or girlfriend, multiple sexual partners, and being aware of contraceptives. |
| Limitations | 19 | Discuss limitations of the study, taking into account sources of potential bias or imprecision. Discuss both direction and magnitude of any potential bias | 34-35 | Sections of students who were on vacation, critically ill, or had mental impairments were excluded from the study. This may be a possible source of bias and may limit generalization of findings of the study. Additionally, we used self-reporting and school environment may have influenced the participants to provide responses that they perceived as appropriate for the investigators. |
| Interpretation | 20 | Give a cautious overall interpretation of results considering objectives, limitations, multiplicity of analyses, results from similar studies, and other relevant evidence | 34 | Based on findings of this study, it is evident that adolescent sexual activity and contraceptive use are significant issues that requires attention and targeted interventions. The prevalence of multiple sexual partners and early sex debut among adolescents provides a reflection of potential risks such as unintended pregnancies, unsafe abortions, and sexually transmitted infections (STIs). On a positive note, there is a high level of awareness and positive attitudes towards contraceptive use among adolescents. However, misconceptions persist, potentially due to unreliable sources of information. Given this, health care providers and other interested stakeholders should use multidimensional and individualized interventions to improve reproductive health of adolescents.  Parents and guardians should be encouraged to educate adolescents on sexual activity and contraceptive use. Collaboration between the Ministry of Health and the Ministry of Education is essential to organize regular health seminars to keep adolescents well-informed on issues related to sexuality and contraception. Additionally, there is a need to reconsider incorporating comprehensive sex education starting at the basic school level. Peer educators should be identified and trained to provide sexual education, as adolescents tend to feel more comfortable seeking information from their peers. |
| Generalisability | 21 | Discuss the generalisability (external validity) of the study results | 49 | Students who were on vacation, critically ill, or had mental impairments were excluded from the study. This may be a possible source of bias and may limit the generalization of findings to all persons living with T2D in Ghana. |
| Other information | |  | | |
| Funding | 22 | Give the source of funding and the role of the funders for the present study and, if applicable, for the original study on which the present article is based | Not included in manuscript. Stated during submission to journal |  |

*Give information separately for cases and controls in case-control studies and, if applicable, for exposed and unexposed groups in cohort and cross-sectional studies.

**Note:** An Explanation and Elaboration article discusses each checklist item and gives methodological background and published examples of transparent reporting. The STROBE checklist is best used in conjunction with this article (freely available on the Web sites of PLoS Medicine at http://www.plosmedicine.org/, Annals of Internal Medicine at http://www.annals.org/, and Epidemiology at http://www.epidem.com/). Information on the STROBE Initiative is available at www.strobe-statement.org.
