# Supplementary material for: Sexual activity and contraceptive use among adolescents: A descriptive survey in a Ghanaian municipality
Source: PLOS Glob Public Health. 2025 Aug 25;5(8):e0005039. doi: 10.1371/journal.pgph.0005039 (PMC12377570; doi:10.1371/journal.pgph.0005039)
Supplement: Attachment — Submitted filename: _PGPH-D-25-00048-1.pdf [file pgph.0005039.s004.pdf]

PLOS Global Public Health

Sexual activity and contraceptive use among adolescents: A descriptive survey in a Ghanaian municipality

--Manuscript Draft--

Manuscript Number:	PGPH-D-25-00048
Article Type:	Research Article
Full Title:	Sexual activity and contraceptive use among adolescents: A descriptive survey in a Ghanaian municipality
Short Title:	Sexual activity and contraceptive use among adolescents
Corresponding Author:	Peter Boakye, BSc. Nursing<br>KNUST: Kwame Nkrumah University of Science and Technology<br>Kumasi, GHANA
Order of Authors:	Peter Boakye, BSc. Nursing
	Evans Adaboh, BSc. Nursing
	Alberta Yemotsoo Lomotey, Master's in public health (MPH)
	Jacob Tetteh, BSc. Nursing
	Abigail Kusi-Amponsah Diji, Dip., BSc., MSc., PhD
	Victoria Bubunyo Bam, PhD
Keywords:	adolescents; sexual activity; contraceptive use
Abstract:	Background<br><br>Contraceptive use is an imperative approach to reducing unwanted pregnancies and sexually transmitted infections (STIs) among adolescents. Sexual activity during adolescence, particularly in developing countries, remains a major public health concern with significant implications for reproductive health. Adolescents aged 10-19 experience heightened sexual drive due to hormonal changes, leading to early sexual activity and low contraceptive uptake, despite widespread knowledge of modern methods.<br><br>Methods<br><br>A cross-sectional study was conducted from August to September 2022, collecting data from 330 Senior High School (SHS) adolescents using simple random sampling. Categorical data were analyzed using frequencies and percentages. Pearson's chi-squared test and binary logistic regression, were employed to assess associations and predict the relationship between independent variables and contraceptive use. Statistical significance was set at p ≤ 0.05 with a confidence interval of 95%.<br><br>Results<br><br>Out of the total participants, 290 (87.9%) were within the age range of 15-19 years and majority of the respondents were females (n=196, 59.4%). Approximately half were in an intimate relationship (n=166, 50.3%) and 126(38%) had ever engaged in sexual intercourse. Of those who have ever engaged in sexual intercourse, over half (n=65, 51.6%) had their first sexual intercourse between the ages of 10-14 years and 100(79.4%) indicated that they have ever used contraceptive. Almost two-thirds of those who had ever used contraceptive reported of having used condom, (n=66, 66.0%), followed by pills, (n=43, 43.0%), and the least used method was IUD (n=2, 2.0%). Adolescents who had boyfriend or girlfriend (AOR=5.604; 95%CL: 2.918-10.763), having multiple sexual partners (AOR=10.631; 95%CL: 5.016-22.532), and being aware of contraceptive (AOR=5.105; 95%CI: 1.533-16.999) had higher odds of contraceptive use than their counterparts.<br><br>Conclusion

Powered by Editorial Manager® and ProduXion Manager® from Aries Systems Corporation

	Early sexual initiation and contraceptive use are prevalent among adolescents. Comprehensive sex education and peer counseling on reproductive health are needed to address the consequences of early sexual activity without contraceptive use.
Additional Information:	
Question	Response
**Financial Disclosure**<br><br>Enter a financial disclosure statement that describes the sources of funding for the work included in this submission and the role the funder(s) played. This includes grants and any commercial funding of the work or authors.<br><br>This statement will be typeset if the manuscript is accepted for publication.<br><br>*Please review the submission guidelines and the instructions link below for detailed requirements and guidance.*	There was no funding
**Competing Interests**<br><br>On behalf of all authors, disclose any competing interests that could be perceived to bias this work.<br><br>This statement will be typeset if the manuscript is accepted for publication.<br><br>*Please review the instructions link below and PLOS Global Public Health's competing interests policy to determine what information must be disclosed at submission.*	The authors received no specific funding for this work.
**Data Availability**<br><br>Before publication, Authors are required to make fully available and without restriction all data underlying their findings. Please see our PLOS Data Policy page for detailed information on	The Data is available in Excel format, and it has been uploaded as a supporting document.

this policy.<br><br>A **Data Availability Statement**, detailing where the data can be accessed, is required at first submission. Insert your Data Availability Statement in the box below.<br><br>Please see the data reporting section of our submission guidelines for instructions on what you need to include in your Data Availability Statement.<br><br>This statement will be typeset if the manuscript is accepted for publication.<br><br>*PLOS allows rare exemptions to address legal and ethical concerns. If you have legal or ethical restrictions, please use the box below to detail these in full sentences for the Journal team to consider.*	

Manuscript Click here to access/download;Manuscript;Manuscript.docx

Full title: Sexual activity and contraceptive use among adolescents: A descriptive survey in a Ghanaian municipality

Short title: Contraceptive use among Senior High school adolescents

Peter Boakye[1*], Evans Adaboh[1], Alberta Yemotsoo Lomotey[1], Jacob Tetteh[1], Abigail Kusi-Amponsah Diji[1], Victoria Bubunyo Bam[1]

[1] School of Nursing and Midwifery, College of Health Sciences, Kwame Nkrumah University of Science and Technology, Kumasi, Ghana

* peterboakye180@gmail.com

ABSTRACT

Background

Contraceptive use is an imperative approach to reducing unwanted pregnancies and sexually transmitted infections (STIs) among adolescents. Sexual activity during adolescence, particularly in developing countries, remains a major public health concern with significant implications for reproductive health. Adolescents aged 10-19 experience heightened sexual drive due to hormonal changes, leading to early sexual activity and low contraceptive uptake, despite widespread knowledge of modern methods.

Methods

A cross-sectional study was conducted from August to September 2022, collecting data from 330 Senior High School (SHS) adolescents using simple random sampling. Categorical data were analyzed using frequencies and percentages. Pearson's chi-squared test and binary logistic regression, were employed to assess associations and predict the relationship between independent variables and contraceptive use. Statistical significance was set at $p \leq 0.05$ with a confidence interval of 95%.

Results

Out of the total participants, 290 (87.9%) were within the age range of 15-19 years and majority of the respondents were females (n=196, 59.4%). Approximately half were in an intimate relationship (n=166, 50.3%) and 126(38%) had ever engaged in sexual intercourse. Of those who have ever engaged in sexual intercourse, over half (n=65, 51.6%) had their first sexual intercourse between the ages of 10-14 years and 100(79.4%) indicated that they have ever used contraceptive. Almost two-thirds of those who had ever used contraceptive reported of having used condom, (n=66, 66.0%), followed by pills, (n=43, 43.0%), and the

least used method was IUD (n=2, 2.0%). Adolescents who had boyfriend or girlfriend (AOR=5.604; 95%CL: 2.918-10.763), having multiple sexual partners (AOR=10.631; 95%CL: 5.016-22.532), and being aware of contraceptive (AOR=5.105; 95%CI: 1.533-16.999) had higher odds of contraceptive use than their counterparts.

Conclusion

Early sexual initiation and contraceptive use are prevalent among adolescents. Comprehensive sex education and peer counseling on reproductive health are needed to address the consequences of early sexual activity without contraceptive use.

Keywords

Adolescents; sexual activity; contraceptive use

Introduction

Adolescent sexual activity is a public health concern that significantly impacts reproductive health outcomes, especially in developing countries such as Ghana(1). Adolescence is a transitional phase of growth and development between childhood and adulthood between the ages of 10 to 19(2). This phase is a very critical period for sexual drive as a result of hormonal changes which occur naturally at this stage of life (3). In Ghana, as in many other African nations, adolescents engage in sexual activity at earlier ages, contributing to a host of challenges, including unintended pregnancies, unsafe abortions and sexually transmitted infections (STIs)(4–6).

According to the World Health Organization(WHO) (2), 37.1% of female adolescents and 21.3% of male adolescents had ever had sex in Ghana. Among these, 9.9% of females and 6.9% of males reported having sex before the age of 15. The number of births per 1,000 adolescent girls was 62.5 in 2020. Additionally, almost 20,000 adolescents were living with HIV and more than 2000 new cases every year(2).

The World Health Organization emphasizes that adolescents have the right to access appropriate information and services regarding sexual and reproductive health. However, lack of comprehensive knowledge, misconceptions, fears, and social and cultural norms including religious prejudices limit adolescents' contraceptive use(7). Additionally, psychological factors, such as the fear of judgement from peers and parents, along with accessibility issues like the location of service points, make adolescents hesitant to discuss or seek contraceptive options(8–10). Consequently, many adolescents engage in unprotected sex, leading to a high prevalence of unintended pregnancies and unsafe abortions, as well as increasing the risk of STIs. Studies indicate that Ghanaian adolescents' contraceptive use

remains low, despite the increasing knowledge about modern contraceptive methods(11–13). Multiple studies conducted among sexually active adolescents and young women reported high unmet needs for modern methods of contraceptives(14,15)

One of the necessary approaches to reduce the burden of the consequences of unprotected sex mentioned above is the use of contraceptives (16). Contraceptive usage is very substantive in preventing unwanted pregnancies, unsafe abortions, and abortion- related complications that expose adolescents to health related risks such as infertility and sometimes death (16). Compared to other regions like Europe, Latin America and the Caribbean, and Northern America contraceptive use in Middle and Western Africa is below 25 percent(17).

Many studies on adolescent sexual behaviour in the Ghanaian context have predominantly focused on adolescent girls and young women (12,18–20). Additionally, studies that included all adolescents have primarily focused on those in late adolescence (16,21,22), failing to capture the full range of adolescent experiences. Notably, research specifically targeting adolescents in senior high schools is lacking. Contraceptive use in Ghana remains low, and the factors influencing contraceptive use among high school adolescents have not been adequately assessed. Therefore, this study aims to fill this gap by examining the sexual activity and contraceptive use among adolescents in senior high schools. Addressing this issue is crucial not only for improving adolescent health outcomes but also for advancing economic growth and long-term national development.

Materials and methods

Study design

A descriptive cross-sectional study was deemed appropriate as the researchers aimed to collect data at a specific point in time and describe the characteristics of adolescents concerning sexual activity and contraceptive use(23).

Study setting

The study was conducted within a senior high school in Ejisu municipality, Ghana, which constitutes both rural and urban localities(24). Senior high school is a second-cycle institution after one graduate from Junior high school. This school was selected as the study setting due to its diverse student population and its ability to cater for students from various economic backgrounds. The school is a public mixed school which admits day students and it also has boarding facilities for a section of the student body. The school offers five (5) programmes which include; Business, Home Economics, Visual Arts, General Arts and General Science. The average yearly enrollment is about 600. Facilities within the school include classrooms, administrative offices, a library, laboratories and outdoor spaces such as playgrounds and sports fields.

Study population

Between 16/08/2022 and 15/09/2022, a total of 330 adolescents participated in the survey. The study population was made up of adolescent from Senior High School. Adolescents were categorized into two age groups; early (10–14 years) and late (15–19 years).

Inclusion and exclusion criteria

Adolescents aged 10 to 19 who were present in school at the time of the study and could speak, understand, and write English were eligible to participate. Sections of students who were on vacation, suspension, critically ill, or had mental impairments were excluded from the study.

Sample size determination and sampling

A sample size of 330 was determined using the Slovin's formula:

$n = \frac{N}{1+N(e)^2}$ (25), and with a confidence interval of 95% (at a Z-score of 1.96), a margin of error of 5%, where **n** signifies the sample size, **N** signifies the population under study and **e** signifies the margin of error (0.05). The total population was 1210 using the total enrollment of students at the time of the study obtained from the school's administrative office. The minimum required sample size obtained was 300. A 10% non-response rate was considered to compensate for a non- response, resulting in a total estimated sample size of 330.

Data collection Tool

A structured questionnaire was used in this study, developed based on current literature and tailored to the study context. The questionnaire was pretested to ensure clarity and relevance. It was divided into six sections the first section was Sociodemographic characteristics of adolescents (7 items): This section includes questions on age, gender, class, religion, ethnicity, residential status (i.e., whom the adolescent lives with), and the employment status of the person the adolescent stays with. Second section was sexual activity (8 items): This section covers topics such as whether the respondent has a boyfriend or girlfriend, the age of their sexual partner, whether they have ever had sex, the number of

sexual partners, the age of first sexual intercourse, how the first sexual experience occurred (whether they were coaxed, forced, or it was their own will), and whether they feel pressured to have sex. Section three was awareness of contraceptives (11 items): Questions in this section include whether the respondent has heard of modern contraceptives, the source of information, the methods of contraception they were aware of, where one can access contraceptives, as well as similar inquiries regarding emergency contraceptives. The fourth section was contraceptive use (11 items): This section includes questions on whether the respondent has ever used contraceptives, which methods they have used, whether they use contraceptives every time they have sex, and the reasons for using them, such as "to avoid teenage pregnancy" or "to prevent STIs." Other questions include whether they used contraceptives during their first sexual intercourse, where they accessed contraceptives, and how frequently they use them, with options like "every time I have sex," "only during my first sexual intercourse," or "once in a while." The fifth section was attitudes of adolescents towards contraceptives (7 Likert scale items): This section includes statements like "I approve of adolescents using contraceptives every time they have sex." Higher scores reflect a positive attitude. Other statements, presented in a negative form and were reverse-coded, include: "Using contraceptives before a girl's first birth can lead to infertility," "Sex is not enjoyable when I use a condom," and "People who insist on condom use are promiscuous." Sixth section was barriers to contraceptive use among adolescents (8 items): Respondents were asked to indicate whether certain factors were barriers to their contraceptive use. These factors include religious beliefs, the attitude of providers, fear of side effects, embarrassment when buying contraceptives, fear of being seen by parents, partner disapproval, unavailability of contraceptives, and cultural beliefs.

Data collection procedure

A structured questionnaire was administered to 330 adolescents attending a Senior High School. A simple random sampling technique was employed to ensure an unbiased selection of participants(26). The researchers first obtained permission from the school administration, and students were briefed on the purpose of the study before participation. Participants were asked to choose between two sealed opaque folded papers one labelled 'yes' and the other 'no'. Those who selected 'yes' after providing informed consent were included in the study.

To minimize potential bias, teachers were asked to leave the classrooms during the questionnaire administration to ensure participants did not feel intimidated or coerced. The questionnaire, comprising 45 questions, was distributed to participants, and they were given a maximum of one hour to complete it. Data was collected in all classes simultaneously to prevent students from discussing the questionnaire's content beforehand. After collection, the completed questionnaires were reviewed by the research team to ensure completeness and accuracy before analysis.

Data processing and analysis

The researchers confirmed the completeness of all collected questionnaires by reviewing each section, ensuring there were no missing data. Serial numbers were assigned to the questionnaire and were entered into Microsoft Excel for data cleaning and transferred to Statistical Product and Service Solutions (SPSS) version 22.0 for Windows (IBM SPSS Statistics) for the data to be analysed. Descriptive statistics (frequencies and percentages) were used to describe the categorical characteristics of participants.

To identify the factors associated with contraceptive use, first chi-square test was done to assess the association between the independent variables and the dependent variable. The ones that were found to be significant were further analyzed using binary logistic regression. Multivariable logistic regression analyses were performed and reported using odds ratio and95% confidence intervals. The significance level was set at a p-value of ≤ 0.05.

Operational definitions

Sociodemographic variables (age, sex, class, religion, ethnicity, residential status [the person adolescent lives with], employment status of the person adolescent lives with).

Adolescent: Individuals aged between **10-19 years**, as defined by the World Health Organization (WHO), who are transitioning from childhood to adulthood and experiencing physical, hormonal, and emotional changes.

Sexual Activity refers to the engagement in any form of sexual intercourse (penile-vaginal), by adolescents. In this study, it includes individuals who reported having had sexual intercourse at least once.

Contraceptive Use: The use of any method (such as condoms, pills, IUDs, injectables, etc.) to prevent pregnancy and reduce the risk of sexually transmitted infections (STIs) during sexual intercourse. In this study, contraceptive use refers to adolescents who reported using any form of contraception.

Quality assurance; Validity

To ensure content validity, we invited six public health experts to assess the relevance of each question to its corresponding factor through a face-to-face approach. This process helped

determine whether the questionnaire accurately measured the intended information. Minor adjustments were made to some questions by all five experts to enhance their cultural relevance and acceptability to the study participants.

Ethical considerations

Prior to the conduct of the study, an institutional-based approval was sought from the management of the school in order to conduct the research in the school. Ethical approval, referenced as CHRPE/AP/426/22, was granted by the Committee on Human Research, Publication, and Ethics (CHRPE) at the School of Medicine and Dentistry, Kwame Nkrumah University of Science and Technology in Kumasi, Ghana. Since majority of the participants resided in the school's boarding house and many parents lived far from the school, direct consent from parents was not feasible. Therefore, verbal consent was obtained from the school authorities acting in loco parentis due to the boarding school arrangement for the participation of the students. The participants were briefed on the research's objectives before seeking their consent to participate. They were provided with an opportunity to inquire about the study procedures. Verbal consent was documented by the research team and witnessed by the teachers who acted as custodians in the school setting. Participation was entirely voluntary, with no direct benefits to participants, and they had the freedom to withdraw at any point. To protect anonymity, no names or other identifying details were obtained.

RESULTS

Sociodemographic characteristics of adolescents

Majority of the participants were in their late adolescents' stage (n=290, 87.9%), and were females (n=196, 59.4%). Most of the participants lived with both parents (n=194, 58.8%), and were Christians (90.9%, n=300). As illustrated in **(Table 1)**

Table 1 Sociodemographic characteristics of adolescents

Variable	Frequency	Percentage (%)
**Age**		
10-14	40	12.1
15-19	290	87.9
**Sex**		
Male	134	40.6
Female	196	59.4
**Class**		
SHS Level 2	190	57.6
SHS Level 3	140	42.4
**Religion**		
Christian	300	90.9
Muslim	30	9.1
**Ethnicity**		
Akan	272	82.4

Ga-Adangbe	12	3.6
Ewe	18	5.5
Others (Dagoba, Mamprusi, Fafra)	28	8.5
**Residential status**		
Living with both Parent	194	58.8
Living with Single parent	120	36.4
Living alone	16	4.8
**Employment status [of the person adolescent lives with]**		
Unemployed	129	39.1
Employed	201	60.9

Sexual activity among adolescents

Half of the participants (n=166, 50.3%) had boyfriends or girlfriends and majority had multiple sexual partners (n=64, 50.2%). Additionally, over a-third of participants (n=126, 38.2%) have ever had sex, and of this, more than half (n=65, 51.6%) experienced their first sexual intercourse during early adolescence, between the ages of 10 and 14 years. Participants who engaged in sex on their own will were (n= 87, 69%). Furthermore, 119 (36.1%) of the participants reported feeling pressured to have sex, predominantly from friends (n=70, 58.8%). As illustrated in **Table 2**

Table 2 Sexual Activity among Adolescents

Variable	Frequency	Percentage (%)

**Having a Boyfriend/Girlfriend**		
Yes<br>No	163<br>167	50.3<br>49.7
**Age of boyfriend/girlfriend (N=166)**		
10-19<br>More than 19	108<br>58	65.0<br>35.0
**Ever had sex**		
Yes<br>No	126<br>204	38.2<br>61.8
**Do you have multiple partners**		
Yes<br>No	65<br>265	19.7<br>80.3
**Number of sexual partners (N=126)**		
1<br>2<br>3<br>More than 3	62<br>24<br>12<br>28	49.2<br>19.0<br>9.6<br>22.2
**Age of first Sexual Intercourse**		
10-14<br>15-19	65<br>61	51.6<br>48.4
**How first sexual intercourse happened (N=126)**		

Own will<br>Coaxed<br>Forced	84<br>17<br>15	66.7<br>13.5<br>11.9
**Pressure to have sex**		
Yes<br>No	119<br>211	36.1<br>63.9
**Sources of pressure to have sex (N=119)**		
Friends<br>Relatives<br>Teachers<br>Boyfriend/Girlfriend	70<br>12<br>10<br>27	58.8<br>10.0<br>8.4<br>22.7

Awareness of contraceptives among Adolescents

Majority of the participants (n=283, 85.8%) surveyed were aware of contraceptive methods. Study results indicated that (n=155, 54.8%) were aware of male condoms; the least (n=12, 4.20%) were aware of IUD. Among those who knew about the contraceptive methods, television (n=144, 50.9%) and peers (n=113, 39.9%) were the major sources of their information and the least were parents (n=10, 5.4%). On whether modern contraceptives provide 100% protection from pregnancy, a notable number of participants, 117(35.5%) reported that they did not know as shown in **Table 3**

Table 3 Awareness of contraceptives among Adolescents

**Variables**	**Frequency**	**Percentage (%)**
**Heard of contraceptives**		
Yes	283	85.8

No	47	14.2
**Heard of any method of contraceptive**		
Yes<br>No	283<br>47	85.8<br>14.2
**Where did you hear it from (N=283)**		
Radio<br>Television<br>Peers<br>Parent<br>Teacher<br>Health worker<br>Internet	105<br>144<br>113<br>62<br>81<br>79<br>113	37.1<br>50.9<br>39.9<br>21.9<br>28.6<br>27.9<br>39.9
**What method of contraception do you know (N=283)**		
Male condom<br>Abstinence<br>Implant<br>Withdrawal method<br>Female condom<br>IUD<br>Urinating after sex<br>Injectable<br>Pills	155<br>35<br>18<br>44<br>40<br>12<br>30<br>20<br>96	54.8<br>12.4<br>6.4<br>15.5<br>14.1<br>4.2<br>10.6<br>7.1<br>33.9
**Heard about emergency contraceptive**		
Yes	185	56.1

No	145	43.9
**If yes, where did you hear about this emergency contraceptive from (N=185)**		
Radio<br>Television<br>Peers<br>Health worker<br>Teacher<br>Internet<br>Parents	88<br>103<br>74<br>73<br>26<br>73<br>10	47.6<br>55.7<br>40<br>39.5<br>14.1<br>39.5<br>5.4
**How often do you know emergency contraceptives can be used within a year (N=185)**		
Once a year<br>Twice a year<br>Three times a year<br>More than three times a year	40<br>48<br>22<br>75	21.6<br>26.5<br>11.4<br>40.5
**Do you know where you can access contraceptive**		
Yes<br>No<br>Unanswered	249<br>40<br>41	75.5<br>12.1<br>12.42
**If yes, where (N=249)**		
Pharmacy<br>Friend<br>Hospital or clinic<br>Health personnel	194<br>51<br>208<br>68	58.8<br>15.5<br>63.0<br>20.6

**Can a girl become pregnant from just one sexual intercourse**		
Yes	240	72.7
No	29	8.8
I don't know	61	18.5
**Do contraceptives provide 100% protection from pregnancy**		
Yes	79	23.9
No	134	40.6
I don't know	117	35.5

Use of contraceptives among adolescents

Among the participants who have ever had sex, 100 (79.4%) had ever used contraceptives. Condoms (n=66, 66%) and pills (n=43, 43%) were the most common methods of contraception. Contraceptive use during the first sexual encounter was reported by 67 (67%). The majority of the participants used contraceptives to avoid pregnancy (n=58, 65.9%). Regarding the frequency of emergency contraceptive usage, 20 (42.3%) indicated they use it every time they have sex**. (Table 4)**

Table 4 Use of contraceptive among adolescents who have had sex

Variable	Frequency	Percentage (%)
**Use of Contraceptives(N=126)**		
Yes	100	79.4
No	26	20.6

**Method of Contraceptive used (N=100)**		
Condom<br>Pills<br>IUD<br>Implants<br>Injectable<br>Withdrawal<br>Safe Period	66<br>43<br>2<br>3<br>3<br>14<br>23	66.0<br>43.0<br>2.0<br>3.0<br>3.0<br>14.0<br>23.0
**Do you use contraceptive anytime you have sex**		
Yes<br>No	50<br>50	50.0<br>50.0
**Reasons for using contraceptives (N=100)**		
To avoid teenage pregnancy<br>To prevent STI	58<br>28	67.4<br>32.6
**Discuss Contraceptive use with your partner(N=100)**		
Yes<br>No<br>I do not remember	59<br>31<br>10	59.0<br>31.0<br>10.00
**Use of contraceptive first time you have sex**		
Yes<br>No	67<br>33	67.0<br>33.0
**Method of Contraceptive use first time of having sex**		

**(N=67)**		
Condom<br>Pills<br>Implants<br>IUD<br>Withdrawal<br>Safe Period<br>Injectable	46<br>32<br>3<br>1<br>12<br>18<br>1	68.7<br>47.8<br>4.5<br>1.5<br>17.9<br>26.7<br>1.5
**Access to Contraceptive(s) (N=67)**		
Hospital<br>Pharmacy<br>Friends<br>Health Personnel	16<br>49<br>22<br>7	23.9<br>73.1<br>32.8<br>10.4
**Frequency of Contraceptive use (N=100)**		
Every time I have sex<br>Only on my first sexual intercourse<br>Once a while	50<br>7<br>43	50.0<br>7.0<br>43.0
**Use of Emergency Contraceptives(N=75)**		
Yes<br>No	47<br>28	62.7<br>37.3
**Frequency of Emergency Contraceptive use (N=47)**		
Every time I have sex	20	42.5

Once a year	10	21.3
Twice a year	9	19.1
Three times a year	4	8.5
More than three times a year	4	8.5

Attitude Towards Contraceptive Use

Participants were asked to indicate their level of agreement to 7 statements on contraceptive use, using a five-point Likert scale rating of strongly agree=5, agree=4, uncertain=3, disagree=2 and strongly disagree=1. All the negative statements coding were reversed, with the minimum, maximum and median scores being 7, 35 and 18 respectively; participants whose overall score was above the median score (18) were said to have a positive overall attitude towards contraceptive use. Detailed results are presented in **Table 5** and **Figure 1** below

Responses Regarding Attitude of Adolescents Towards Contraceptive Use

From the survey, majority (n= 80, 24.2%) agree with the usage of contraceptives by adolescents. Most of the participants (n=129, 39.1%) were uncertain that the use of contraceptives by a girl before her first birth can lead to infertility. Approximately, 117(35%) of the respondents were not certain as to whether “sex is not enjoyable when I use condom”. Participants whose religion was against usage of contraceptives were (n=141, 42.7%), and 207(62.7%) reported contraceptive use is not only the responsibility of females.

Table 5 Attitude of adolescents towards contraceptive use

Variable	Frequency

	Strongly disagree	Disagree	Uncertain	Agree	Strongly agree
I approve the use of contraceptive by adolescents anytime they have sex [preferred]	53(16.1%)	73(22.1%)	58(17.6%)	80(24.2%)	66(20.0%)
Use of contraceptive by a girl before her first birth can lead to infertility	35(10.6%)	46(13.9%)	129(39.1%)	73(22.1%)	47(14.2%)
Sex is not enjoyable when I use condom	51(15.5%)	30(9.1%)	117(35.3%)	71(21.5%)	61(18.5%)
People who insist on condom use are promiscuous	44(13.3%)	68(20.6%)	86(26.1%)	68(20.6%)	64(19.4%)
Contraception usage is against my religion	59(17.9)	75(22.7%)	55(16.7%)	74(22.4%)	67(20.3%)

I will use contraception in the future	96(29.1%)	79(23.9%)	79(23.9%)	47(14.2%)	29(8.8%)
Contraception is the responsibility of only females	125(37.9%)	82(24.8%)	51(15.5%)	42(12.7)	30(9.1%)

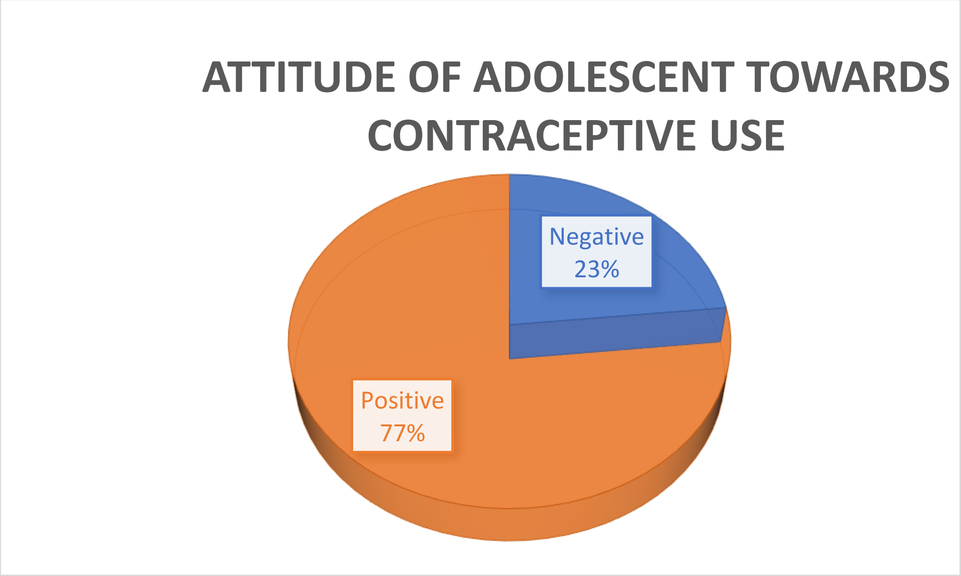

*Figure 1: Attitude of adolescent towards contraceptive use**

Barriers to contraceptive use among adolescents

Among these barriers to the use of contraceptives, majority of the respondent reported fear of side effects of contraceptive as a barrier (n=16, 63.2%). Other barriers were religious beliefs (n=14, 55.3%) and embarrassment on the part of adolescents (n=9, 35.5%). The least

reported barrier to the use of contraceptive was unavailability of contraceptive (n= 2, 9.2%) as presented in **Table 6.**

Table 6 Barriers to contraceptive use among adolescents

Variable	Frequency	Percentage
Reason(s) for not using any of the contraceptive methods (N=26)		
Religious beliefs	14	55.3
Attitude of contraceptive providers	3	14.5
Fear of side effects	16	63.2
Embarrassment to buy	9	35.5
Fear of being seen by parents	8	30.3
Partner disapproval	4	17.1
Unavailability of contraceptive	2	9.2
Cultural beliefs	7	28.9

Association between socio-demographic characteristics of Adolescents and use of contraceptives

The study found that having a boyfriend or girlfriend, the number of sexual partners an adolescent had, and awareness of modern contraceptives (all with a p-value of $p \leq 0.001$) were statistically significant factors influencing contraceptive use. There was no significant

association between some of the sociodemographic characteristics (age, sex, class, religion, ethnicity, residential status, and employment status of the person adolescent lives with) **(Table 7)**

Table 7 Association between socio-demographic characteristics of Adolescents and use of contraceptives

Variables	Usage of contraceptive		Chi-square (p-value)
	Yes, n (%)	No, n (%)	
Age			
14-16	8(20.0)	32(80.0)	0.309
17-19	92(31.7)	198(68.3)	
Sex			
Male	41(30.6)	93(69.4)	0.700
Female	59(30.1)	137(69.9)	
Class			
SHS 2	57(29.8)	134(70.2)	0.666
SHS 3	43(30.9)	96(69.1)	
Religion			
Christian	88(29.3)	212(70.7)	0.220
Muslim	12(40.0)	18(60.0)	
Ethnicity			
Akan	77(31.7)	166(68.3)	0.887

Fante<br>Ga-Adangbe<br>Ewe<br>Others (Dagoba, mamprusi, Fafra)	6(20.7)<br>5(41.7)<br>6(33.3)<br>7(25.9)	23(79.3)<br>7(58.3)<br>12(66.7)<br>21(74.1)	
Residential status			
Both parent<br>Single parent<br>Live alone	51(26.3)<br>41(34.1)<br>8(50.0)	143(73.7)<br>79(65.9)<br>8(50.0)	0.072
Employment status			
Unemployed<br>Employed	41(31.8)<br>59(29.4)	88(60.2)<br>142(70.6)	0.920
Do you have a boyfriend/girlfriend			
Yes<br>No	87(52.4)<br>13(7.9)	79(47.6)<br>151(92.1)	≤0.001*
Number of sexual partners			
0<br>1<br>2<br>3	0(0.0)<br>44(71.0)<br>18(75.0)<br>8(66.7)	204(100.0)<br>18(29.0)<br>6(25.0)<br>4(33.3)	≤0.001*

More than 3	25(89.2)	3(10.8)	
Heard of modern contraceptive			
Yes	97(65.7)	186(34.3)	≤0.001*
No	3(6.3)	44(93.7)	

Logistic regression predicting modern contraceptive use

The model explained between 43.3% (Cox & Snell R^2) and 61.4% (Nagelkerke R^2) of the variance in modern contraceptive use. The Hosmer-Lemeshow test produced a p-value of 0.512, indicating that the model fits the data adequately. Overall, the model correctly classified 90.0% of cases. The independent variables, whether the respondent has a boyfriend or girlfriend, the number of sexual partners, and whether the respondent has heard of modern contraceptives were statistically significant predictors in the model. Having heard of modern contraceptives, has an odds ratio of 5.105 indicating that adolescents who have heard of modern contraceptives are 5.105 times higher odds to use contraceptive compared to those who have not. Having multiple sexual partners, with an odds ratio of 10.631, indicating that those with multiple sexual partners has a higher odd to use contraceptive. Finally, the variable having a boyfriend or girlfriend had an odds ratio of 5.604, meaning adolescents with a boyfriend or girlfriend has 5.604 higher odds adjusting for other variables in the equation to use contraceptives than those without. **(Table 8)**

Table 8 Logistic regression predicting modern contraceptive use

Variable	B(Coefficient)	Adjusted Odds Ratio (EXP(B))	95%CL	p-value
Heard of modern contraceptive (Yes)	1.630	5.105	1.533-16.999	0.016
Do you have multiple partners (Yes)	2.364	10.631	5.016-22.532	< 0.001
Do you have boyfriend (Yes)	1.724	5.604	2.918-10.763	0.006

Discussion

This study aimed to assess the sexual activity and contraceptive use among adolescents. Findings from the study showed that, a little over half of adolescents are in a relationship with the opposite sex. This corroborates findings of other studies that showed that, majority of adolescents had ever been in a relationship with someone of the opposite sex (27,28).

Furthermore, the study found that, among adolescents who have had sex, majority of them had their sexual debut at an early adolescence stage (10-14 years). This could be explained by the exploration and heightened sexual curiosity among adolescents during their critical

growth phase of identity formation. A study conducted in Kenya reported that adolescents who initiate sex at an early stage are influenced by peer pressure, as well as easy access to phones and the internet at an early age(29). This may be due to increased exposure to erotic materials (nudity, photographs/videos of sexual activity), which are readily available. This increases their curiosity and finally commit to the act of sexual activity. This is further supported by the findings of this current study, which revealed that adolescents mostly feel pressurized from their friends and their boyfriend or girlfriend to have sex.

The study also reported high percentage of adolescents having multiple sexual partners. This is consistent with Attibu (27) who reported that nearly half of adolescent in the study were having more than one sexual partner. This can be an indicator for risk of pregnancy, unsafe abortion, and STIs, implying that adolescents do not maintain long-term sexual relationships but instead may enter new relationships due to unmet expectations, such as financial rewards or benefits (30).

The findings from this study revealed that, awareness of contraceptive was high among adolescents. This is in keeping with a study conducted by Agbanyo Agbanyo, (2018) which revealed that, 88% were aware of contraceptives. From our findings, most of the adolescents who have heard about contraception, 86.1% were able to mention at least one type of contraceptive and where it can be accessed. This high awareness of contraception could be attributed to the recent change in social media advertisement which includes both video and audio.

In this study, the most mentioned sources of information on contraceptives were television, peers and radio which is similar to research conducted in Tanzania (32). Also research by

Hagan & Buxton, indicated that, majority (60.0%) said they got their information through the radio or television(33). The least source of information on contraceptives use was found to be from parents. This is in agreement with a study conducted by Hagan & Buxton, which also revealed that, only 3.3% of adolescents received information on contraceptive from parents(33). This could be as a result of African cultural setting where discussing sexual issues in the house is seen as an immoral behaviour (34). Sometimes, this makes it uncomfortable for both adolescents and the elderly to share ideas concerning sexuality and contraceptive use. The source of information is a major factor in providing adolescents with correct information regarding contraceptives and its use. A reliable source of information from health care providers and mass media provide adolescents with accurate information than friends (32).

From the findings of the current study, the prevalence of contraceptive use among adolescents was high which is currently above Ghana Health Service national family planning target of 23.3% and higher than other studies conducted among adolescents(35,36). This is in agreement with findings from a study by Maness et al, which also showed high usage of contraceptives by adolescents(37). This is inconsistent with other studies which revealed low use of contraceptives among adolescents(35). Findings from a study by Kinaro et al (38) revealed that only 8.6% of all adolescents had ever used a contraceptive method. The high use of contraceptives may probably be attributed to the high level of awareness as revealed by findings of the current study.

Condom and Pills were the mostly used contraceptives by adolescents. Condoms were the most used method of contraception probably because it is the most popular, easily accessible,

cheap and have dual protection against teenage pregnancy and sexually transmitted diseases (39). The majority of adolescents access contraceptives from the pharmacy and peers. This is in line with the findings of Kara et al(32). Adolescents prefer to access condoms and pills from pharmacy shops probably because it is easily accessible in almost every community as compared to hospitals. This corroborates with a study conducted by Chandra-Mouli & Akwara, which revealed that pharmacies offer adolescents accessible locations, longer operating hours and more importantly anonymity in obtaining contraceptive methods(40). The study further indicated that the preference for pharmacies was as a result of difficulty in obtaining contraceptives from health facilities due to restrictive laws, health worker bias, or privacy concerns

Adolescents exhibited positive attitudes towards contraceptive usage. The present study reported that, majority of adolescents approved the “use contraceptive anytime they have sex”. This findings is in contrast to a previous study by Sam, where the level and attitude towards the use of contraceptives among adolescents were found to be very low and only few of them have ever used or currently using effective contraceptive methods(41).

However, few participants exhibited negative attitudes towards contraceptive use. For instance, majority of adolescents in this study supported the idea that the use of contraceptive by a girl before her first birth can lead to infertility. This is in agreement with a study conducted by Sam, where 67.0% of respondents strongly agreed that a girl using contraceptives before having her first child can cause infertility(41). This is also consistent with Lauren et al, who found that future fertility was among the main attitudes which tend to be an obstacle to the use of contraceptives(42). Adolescents have a misconception about

contraceptive use. Perhaps, this is because information on contraception given by unreliable sources like friends who lacked in-depth knowledge was high. This may also be as a result of the side effects of contraceptives (especially oral pills and emergency contraceptives) on the menstrual cycle of female adolescents.

With regard to contraceptive use, awareness, number of sexual partners, and having a boyfriend or girlfriend were found to influence adolescents' use of contraceptives. This finding contrasts with a study conducted in Nigeria by Abdul et al among university students, which revealed no association between awareness and contraceptive use(43).

Research conducted by James et al. showed a significant association between the number of sexual partners and contraceptive use(44). Adolescents with two or more sexual partners are more likely to engage in risky sexual behaviors (45). To prevent unintended pregnancies, STIs, school dropout, and unsafe abortions, they often resort to contraceptive use. This aligns with the findings of the current study, where adolescents with multiple partners were found to be four times more likely to use contraceptives. Additionally, all adolescents who had ever used contraceptives indicated either the prevention of unwanted pregnancy or the prevention of STIs as their primary reason for use.

Being in long-term or casual relationships is also significantly associated with effective contraceptive use (28). This finding is consistent with the results of the current study, where having a boyfriend or girlfriend increased an adolescent's likelihood of using contraceptives by approximately three times.

Conclusion and recommendation

Based on the findings of this study, it is evident that adolescent sexual activity and contraceptive use are significant issues that require attention and targeted interventions. The prevalence of multiple sexual partners and early sex debut among adolescents reflects potential risks such as unintended pregnancies, unsafe abortions, and sexually transmitted infections (STIs). On a positive note, there is a high level of awareness and positive attitudes towards contraceptive use among adolescents. However, misconceptions persist, potentially due to unreliable sources of information. Given this, healthcare providers and other interested stakeholders should use multidimensional and individualized interventions to improve the reproductive health of adolescents.

Parents and guardians should be encouraged to educate adolescents on sexual activity and contraceptive use. Collaboration between the Ministry of Health and the Ministry of Education is essential to organize regular health seminars to keep adolescents well-informed on issues related to sexuality and contraception. Additionally, there is a need to reconsider incorporating comprehensive sex education starting at the basic school level. Peer educators should be identified and trained to provide sexual education, as adolescents tend to feel more comfortable seeking information from their peers. Finally, future studies should explore the role of parents and guardians in influencing adolescents' sexual activity and contraceptive use.

Efforts should be directed towards providing training to health workers and educators on how to effectively communicate accurate and evidence-based information to parents and the

general public. This can include workshops on health communication and the use of trusted sources.

Limitations of the study

The use of self-reporting and school environment may have influenced the participants to provide responses that they perceived as socially desirable. However, the findings of the current research have relevant implications for improving adolescent sexual and reproductive health. Future research should prioritize assessing sexual activity and contraceptive use beyond the school setting.

Acknowledgements

The authors sincerely thank the management of the school for granting us the opportunity to conduct this study in their esteemed institution. We also extend our heartfelt gratitude to the students who participated in this study.

References

1. Alhassan AR, Wepeba TW, Abdulai K, Iddrisu R, Aninanya GA. Prevalence and Associated Factors of Adolescent (15-19 Years) Childbearing in Ghana. Biomed Res Int. 2024;2024.
2. WHO. Ghana Contraception within the context of adolescents' sexual and reproductive lives: Country profile Microsoft Word - UGANDA. 2021;5:9–11. Available from: who.int
3. Vijayakumar N, Op de Macks Z, Shirtcliff EA, Pfeifer JH. Puberty and the human brain:

Insights into adolescent development. Neurosci Biobehav Rev [Internet]. 2018;92(January):417–36. Available from: https://doi.org/10.1016/j.neubiorev.2018.06.004

4. Kyei-Arthur F, Agyekum MW, Kyei-Gyamfi S. "You cannot stay with one person once you begin having sex at a young age": the prevalence, correlates and effects of early sexual debut among children in Ghana. Reprod Health [Internet]. 2024;21(1):1–10. Available from: https://doi.org/10.1186/s12978-024-01775-4

5. Pringle J, Mills KL, McAteer J, Jepson R, Hogg E, Anand N, et al. The physiology of adolescent sexual behaviour: A systematic review. Cogent Soc Sci. 2017;3(1):1–14.

6. Kyilleh JM, Tabong PTN, Konlaan BB. Adolescents' reproductive health knowledge, choices and factors affecting reproductive health choices: A qualitative study in the West Gonja District in Northern region, Ghana. BMC Int Health Hum Rights. 2018;18(1):1–12.

7. Wondimagegne YA, Debelew GT, Koricha ZB. Barriers to contraceptive use among secondary school adolescents in Gedeo zone, South Ethiopia: A formative qualitative study. BMJ Open. 2023;13(3):1–10.

8. Masonbrink AR, Hurley EA, Schuetz N, Rodean J, Rupe E, Lewis K, et al. Sexual behaviors, contraception use and barriers among adolescents and young adults in rural Haiti. BMC Womens Health. 2023;23(1):1–10.

9. Dandala S, Hodambia M. Journal Wetenskap Health. J Wet Heal [Internet]. 2021;4(4):22–7. Available from:

https://pdfs.semanticscholar.org/dc01/0a49f45e1d6d020306d577378e24a2bd59ea.pdf

10. James PB, Osborne A, Babawo LS, Bah AJ, Margao EK. The use of condoms and other birth control methods among sexually active school-going adolescents in nine sub-Saharan African countries. BMC Public Health [Internet]. 2022;22(1):1–11. Available from: https://doi.org/10.1186/s12889-022-14855-6

11. Agbanyo Ri. KNOWLEDGE AND BARRIERS TO CONTRACEPTIVE USE AMONG IN-SCHHOL ADOLESCENT IN ADAKLU DISTRICT. 2018;(10637685):1–74.

12. Amoah EJ, Hinneh T, Aklie R. Determinants and prevalence of modern contraceptive use among sexually active female youth in the Berekum East Municipality, Ghana. PLoS One [Internet]. 2023;18(6 JUNE):1–18. Available from: http://dx.doi.org/10.1371/journal.pone.0286585

13. Beson P, Appiah R, Adomah-Afari A. Modern contraceptive use among reproductive-aged women in Ghana: Prevalence, predictors, and policy implications. BMC Womens Health. 2018;18(1):1–8.

14. Alidou S, Dahourou LD, Dah TTE, Sogo A, Kenao TS, Yehadji D, et al. Unmet needs for modern contraceptive methods among sexually active adolescents and young women in Togo: a nationwide cross-sectional study. Front Public Heal. 2023;11(July).

15. Atiglo DY, Biney AAE. Correlates of sexual inactivity and met need for contraceptives among young women in Ghana. BMC Womens Health. 2018;18(1):1–10.

16. Mahama E, Owusu-agyei S. Use of contraceptives among adolescents in Kintampo ,

Ghana : a cross-sectional study. 2014;7–15.

17. WHO. Contraception Evidence brief. 2019;(3).

18. Agyemang J, Newton S, Nkrumah I, Tsoka-Gwegweni JM, Cumber SN. Contraceptive use and associated factors among sexually active female adolescents in Atwima Kwanwoma District, Ashanti region-Ghana. Pan Afr Med J. 2019;32:1–13.

19. Sumankuuro J, Asuuri A, Mikare MD, Ngmenkpieo F, Crockett J, Wulifan JK. High School Student's Knowledge, Attitude and Participation in Sexual Health Education in Rural Northern Ghana. J Biosci Med. 2020;08(05):64–83.

20. Keogh SC, Otupiri E, Castillo PW, Li NW, Apenkwa J, Polis CB. Contraceptive and abortion practices of young Ghanaian women aged 15–24: evidence from a nationally representative survey. Reprod Health [Internet]. 2021;18(1):1–17. Available from: https://doi.org/10.1186/s12978-021-01189-6

21. Oppong FB, Logo DD, Agbedra SY, Adomah AA, Amenyaglo S, Arhin- K, et al. Determinants of contraceptive use among sexually active unmarried adolescent girls and young women aged 15 – 24 years in Ghana : a nationally representative cross- - sectional study. 2021;1–10.

22. Li Z, Patton G, Sabet F, Zhou Z, Subramanian S V., Lu C. Contraceptive Use in Adolescent Girls and Adult Women in Low- And Middle-Income Countries. JAMA Netw Open. 2020;3(2):1–15.

23. Wang X, Cheng Z. Cross-Sectional Studies: Strengths, Weaknesses, and Recommendations. Chest [Internet]. 2020;158(1):S65–71. Available from:

https://doi.org/10.1016/j.chest.2020.03.012

24. Ghana Statistical Service. Ghana 2021 Population and Housing Census. 2021;6.

25. Slovin's. Slovin formula in research pdf. 1960;

26. Krishna R, Maithreyi R, Surapaneni KM. Research bias: A review for medical students. J Clin Diagnostic Res. 2010;4(2):2320–4.

27. Attibu RIE. Sexual Behaviour among Adolescents in Senior High Schools within the Tamale Metropolis, Northern Region of Ghana. 2015;(10268216):1–98.

28. Upadhyay UD, Raifman S, Raine-Bennett T. Effects of relationship context on contraceptive use among young women. Contraception [Internet]. 2016;94(1):68–73. Available from: http://dx.doi.org/10.1016/j.contraception.2016.02.025

29. Mauti G, Muthoni S. Early Sexual Debut and the Associated Factors among School Going Adolescents in Selected Schools in Kenya. East African Heal Res J. 2023;7(2):309–17.

30. Vincent G, Alemu FM. Factors contributing to, and effects of, teenage pregnancy in Juba. South Sudan Med J. 2016;9(2):28–31.

31. Richmond Agbanyo. Knowledge and barriers to contraceptive use among in school adolescent in Adaklu District. 2018;7(2):1–74.

32. Kara WSK, Benedicto M, Mao J. Knowledge, Attitude, and Practice of Contraception Methods Among Female Undergraduates in Dodoma, Tanzania. Cureus. 2019;11(4).

33. Hagan JE, Buxton C. Contraceptive Knowledge, Perceptions and Use among

Adolescents in Selected Senior High Schools in the Central Region of Ghana. J Sociol Res. 2012;3(2):170–80.

34. Khumalo S, Taylor M, Makusha T, Mabaso M. Intersectionality of cultural norms and sexual behaviours: a qualitative study of young Black male students at a university in KwaZulu-Natal, South Africa. Reprod Health. 2020;17(1):1–10.

35. Nkenguye W, Ismail H, Urassa EP, Yongolo NM, Kagoye S, Msuya SE. Factors Associated with Modern Contraceptive Use Among Out of School Adolescent Girls in Majengo and Njoro Wards of Moshi Municipality, Tanzania. East African Heal Res J. 2023;7(1):32–9.

36. Michael TO, Ojo TF, Ijabadeniyi OA, Ibikunle MA, Oni JO, Agboola AA. Prevalence and factors associated with contraceptive use among sexually active adolescent girls in 25 sub-Saharan African countries. PLoS One [Internet]. 2024;19(2 February):1–15. Available from: http://dx.doi.org/10.1371/journal.pone.0297411

37. Maness SB, Thompson EL, Lu Y. Associations Between Social Determinants of Health and Adolescent Contraceptive Use: An Analysis From the National Survey of Family Growth. Fam Community Heal. 2022;45(2):91–102.

38. Kinaro J, Kimani M, Ikamari L, Ayiemba EHO. Perceptions and Barriers to Contraceptive Use among Adolescents Aged 15 - 19 Years in Kenya: A Case Study of Nairobi. Health (Irvine Calif). 2015;07(01):85–97.

39. Beksinska M, Wong R, Smit J. Male and female condoms: Their key role in pregnancy and STI/HIV prevention. Best Pract Res Clin Obstet Gynaecol. 2020;66:55–67.

40. Chandra-Mouli V, Akwara E, Engel D, Plessons M, Asnake M, Mehra S, et al. Progress in adolescent sexual and reproductive health and rights globally between 1990 and 2016: what progress has been made, what contributed to this, and what are the implications for the future? Sex Reprod Heal Matters [Internet]. 2020;28(1). Available from: https://doi.org/10.1080/26410397.2020.1741495

41. Sam NB. Knowledge and Awareness Level of Contraceptive Usage Among Adolescents in Mankranso of Ahafo-Ano South District. Biomed J Sci Tech Res. 2020;31(4).

42. Chernick S L, Orav EJ, Brennan TA, Burstin HR. in an Emergency Department. J Gen Intern Med. 1999;14(3):82–7.

43. Abdul I, Imohagene A, Nigeria LO… S of, 2018 undefined. Awareness, knowledge and contraceptive use among female students in. JesonOrgNg [Internet]. 2018;1(2). Available from: https://jeson.org.ng/index.php/jeson/article/view/17

44. James PB, Osborne A, Babawo LS, Bah AJ, Margao EK. The use of condoms and other birth control methods among sexually active school-going adolescents in nine sub-Saharan African countries. BMC Public Health. 2022;22(1):1–13.

45. Cruzeiro ALS, Souza LD de M, Silva RA da, Pinheiro RT, Rocha CLA da, Horta BL. Comportamento sexual de risco: fatores associados ao número de parceiros sexuais e ao uso de preservativo em adolescentes. Cien Saude Colet. 2010;15(suppl 1):1149–58.

Supporting Information

S-1 File (DOCX)

S-2 File (DOCX)

S- 3 Data (XLS)

S-4 File (DOCX)

S-5 File (DOCX)

Click here to access/download
Supporting Information
S-1.docx

Click here to access/download
Supporting Information
S-2.docx

Click here to access/download
Supporting Information
S-3.xlsx

Click here to access/download
Supporting Information
S-4.docx

Click here to access/download
Supporting Information
S-5.docx
