# Supplementary material for: Sexual activity and contraceptive use among adolescents: A descriptive survey in a Ghanaian municipality
Source: PLOS Glob Public Health. 2025 Aug 25;5(8):e0005039. doi: 10.1371/journal.pgph.0005039 (PMC12377570; doi:10.1371/journal.pgph.0005039)
Supplement: Attachment — Submitted filename: Manuscriptt.pdf [file pgph.0005039.s007.pdf]

PLOS Global Public Health

Sexual Activity and Contraceptive Use Among Adolescents: A Descriptive Survey in a Ghanaian Municipality

--Manuscript Draft--

**Manuscript Number:**	PGPH-D-25-00048R1
**Article Type:**	Research Article
**Full Title:**	Sexual Activity and Contraceptive Use Among Adolescents: A Descriptive Survey in a Ghanaian Municipality
**Short Title:**	Contraceptive Use Among Senior High School Adolescents
**Corresponding Author:**	Peter Boakye, BSc. Nursing<br>KNUST: Kwame Nkrumah University of Science and Technology<br>Kumasi, GHANA
**Order of Authors:**	Peter Boakye, BSc. Nursing
	Evans Adaboh, BSc. Nursing
	Alberta Yemotsoo Lomotey, Master's in public health (MPH)
	Jacob Tetteh, BSc. Nursing
	Abigail Kusi-Amponsah Diji, Dip., BSc., MSc., PhD
	Victoria Bubunyo Bam, PhD
**Keywords:**	Adolescents; Sexual activity; Contraceptive use; High School; Reproductive health; Ghana
**Abstract:**	Contraceptive use is an imperative approach to reducing unwanted pregnancies and sexually transmitted infections (STI's) among adolescents. Sexual activity during adolescence, particularly in developing countries, remains a major public health concern with significant implications for reproductive health. Adolescents aged 10-19 experience heightened sexual drive due to hormonal changes, leading to early sexual activity and low contraceptive uptake, despite widespread knowledge of modern methods. The study aims to assess sexual activity and contraceptive use among Senior High School (S.H.S) adolescents, and to identify key predictors of contraceptive use. The study addresses gaps in literature regarding factors influencing contraceptive use among S.H.S adolescents, a group often overlooked in previous studies. A cross-sectional study was conducted from August to September 2022, involving 330 S.H.S adolescents selected through simple random sampling. Categorical data were analyzed using frequencies and percentages. Pearson's chi-squared test and binary logistic regression, were employed to assess associations and predict the relationship between independent variables, including sociodemographic characteristics (age, sex, class, ethnicity, religion, residential status), sexual activity characteristics (relationship status, multiple sexual partners), awareness of contraceptives and contraceptive use. Statistical significance was set at $p \leq 0.05$ with a confidence interval of 95%. Out of the total participants, 290 (87.9%) were within the age range of 15-19 years and majority of the respondents were females (n=196, 59.4%). Approximately half (n=166, 50.3%) were in an intimate relationship and 126(38%) had ever engaged in sexual intercourse. Of those who have ever engaged in sexual intercourse, over half (n=65, 51.6%) had their first sexual intercourse between the ages of 10-14 years and 100(79.4%) reported having ever used contraceptive. Almost two-thirds of those who had ever used contraceptive reported of having used condom, (n=66, 66.0%), followed by pills, (n=43, 43.0%), with IUD being the least used method (n=2, 2.0%). Adolescents who had boyfriend or girlfriend (AOR=7.208; 95%CL: 3.533-14.706), had multiple sexual partners (AOR=9.518; 95%CL: 4.320-20.969), and were aware of contraceptive (AOR=4.655; 95%CI: 1.395-15.530) had higher odds of contraceptive use than their counterparts. Early sexual initiation and contraceptive use are prevalent among adolescents. Comprehensive sex education and peer counseling on reproductive health are needed to address the consequences of early sexual activity without contraceptive use.

Powered by Editorial Manager® and ProduXion Manager® from Aries Systems Corporation

Additional Information:	
Question	Response
**Financial Disclosure**<br><br>Enter a financial disclosure statement that describes the sources of funding for the work included in this submission and the role the funder(s) played. This includes grants and any commercial funding of the work or authors.<br><br>This statement will be typeset if the manuscript is accepted for publication.<br><br>*Please review the submission guidelines and the instructions link below for detailed requirements and guidance.*	There was no funding
**Competing Interests**<br><br>On behalf of all authors, disclose any competing interests that could be perceived to bias this work.<br><br>This statement will be typeset if the manuscript is accepted for publication.<br><br>*Please review the instructions link below and PLOS Global Public Health's competing interests policy to determine what information must be disclosed at submission.*	The authors declare no competing interests
**Data Availability**<br><br>Before publication, Authors are required to make fully available and without restriction all data underlying their findings. Please see our PLOS Data Policy page for detailed information on this policy.<br><br>A **Data Availability Statement**, detailing	The Data is available in Excel format, and it has been uploaded as a supporting document.

Powered by Editorial Manager® and ProduXion Manager® from Aries Systems Corporation

where the data can be accessed, is required at first submission. Insert your Data Availability Statement in the box below.<br><br>Please see the data reporting section of our submission guidelines for instructions on what you need to include in your Data Availability Statement.<br><br>This statement will be typeset if the manuscript is accepted for publication.<br><br>*PLOS allows rare exemptions to address legal and ethical concerns. If you have legal or ethical restrictions, please use the box below to detail these in full sentences for the Journal team to consider.*	

Powered by Editorial Manager® and ProduXion Manager® from Aries Systems Corporation

Manuscript Click here to access/download;Manuscript;Manuscript.docx

Full title: Sexual activity and contraceptive use among adolescents: A descriptive survey in a Ghanaian municipality

Short title: Contraceptive use among Senior High School adolescents

Peter Boakye[1*], Evans Adaboh[1], Alberta Yemotsoo Lomotey[1], Jacob Tetteh[1], Abigail Kusi-Amponsah Diji[1], Victoria Bubunyo Bam[1]

[1] School of Nursing and Midwifery, College of Health Sciences, Kwame Nkrumah University of Science and Technology, Kumasi, Ghana

* peterboakye180@gmail.com

ABSTRACT

Contraceptive use is an imperative approach to reducing unwanted pregnancies and sexually transmitted infections (STIs) among adolescents. Sexual activity during adolescence, particularly in developing countries, remains a major public health concern with significant implications for reproductive health. Adolescents aged 10-19 experience heightened sexual drive due to hormonal changes, leading to early sexual activity and low contraceptive uptake, despite widespread knowledge of modern methods. The study aims to assess sexual activity and contraceptive use among Senior High School (SHS) adolescents, and to identify key predictors of contraceptive use. The study addresses gaps in literature regarding factors influencing contraceptive use among SHS adolescents, a group often overlooked in previous studies. A cross-sectional study was conducted from August to September 2022, involving 330 SHS adolescents selected through simple random sampling. Categorical data were analyzed using frequencies and percentages. Pearson's chi-squared test and binary logistic regression, were employed to assess associations and predict the relationship between independent variables, including sociodemographic characteristics (age, sex, class, ethnicity, religion, residential status), sexual activity characteristics (relationship status, multiple sexual partners), awareness of contraceptives and contraceptive use. Statistical significance was set at $p \leq 0.05$ with a confidence interval of 95%. Out of the total participants, 290 (87.9%) were within the age range of 15-19 years and majority of the respondents were females (n=196, 59.4%). Approximately half (n=166, 50.3%) were in an intimate relationship and 126(38%) had ever engaged in sexual intercourse. Of those who have ever engaged in sexual intercourse, over half (n=65, 51.6%) had their first sexual intercourse between the ages of 10-14 years and 100(79.4%) reported having ever used contraceptive. Almost two-

thirds of those who had ever used contraceptive reported of having used condom, (n=66, 66.0%), followed by pills, (n=43, 43.0%), with IUD being the least used method (n=2, 2.0%). Adolescents who had boyfriend or girlfriend (AOR=7.208; 95%CL: 3.533-14.706), had multiple sexual partners (AOR=9.518; 95%CL: 4.320-20.969), and were aware of contraceptive (AOR=4.655; 95%CI: 1.395-15.530) had higher odds of contraceptive use than their counterparts. Early sexual initiation and contraceptive use are prevalent among adolescents. Comprehensive sex education and peer counseling on reproductive health are needed to address the consequences of early sexual activity without contraceptive use.

Keywords

Adolescents; sexual activity; contraceptive use; High School; Reproductive Health, Ghana

1.0 Introduction

Adolescent sexual activity is a global public health concern that significantly impacts reproductive health outcomes(1), especially in developing countries such as Ghana(2). Globally, more than 16 million adolescent girls aged 15–19 years give birth each year, most of which occur in Low-and Middle-Income Countries (LMICs)(3,4). Adolescence is a transitional phase of growth and development between childhood and adulthood between the ages of 10 to 19(5). This phase is a very critical period for sexual drive as a result of hormonal changes which occur naturally at this stage of life (6). In Ghana, as in many other countries globally, adolescents engage in sexual activity at earlier ages, often initiating sexual intercourse before the age of 15(7,8), contributing to a host of challenges, including unintended pregnancies, unsafe abortions and sexually transmitted infections (STIs)(7,9).

According to the World Health Organization(WHO) (5), 37.1% of female adolescents and 21.3% of male adolescents had ever had sex in Ghana. Among these, 9.9% of females and 6.9% of males reported having sex before the age of 15. In 2020, the adolescent birth rate was 62.5 per 1,000 adolescent girls, and nearly 20,000 adolescents were living with HIV, with more than 2,000 new cases reported annually (5).

The World Health Organization emphasizes that adolescents have the right to access appropriate information and services regarding sexual and reproductive health(10). However, lack of comprehensive knowledge, misconceptions, fears, and social and cultural norms including religious prejudices limit adolescents' contraceptive use(11). Additionally, psychological factors, such as the fear of judgement from peers and parents, fear of side effects, and accessibility issues like the location of service points, make adolescents hesitant to discuss or seek contraceptive options(12–14). Consequently, many adolescents engage in unprotected sex, leading to a high prevalence of unintended pregnancies and unsafe abortions, as well as increased the risk of STIs(15,16). Studies indicate that contraceptive use among Ghanaian adolescents remains low, despite the increasing knowledge about modern contraceptive methods(17–19). Multiple studies conducted among sexually active adolescents and young women reported high unmet need for modern contraceptive methods(20,21)

One of the necessary approaches to reducing the burden of the consequences of unprotected sex is the use of contraceptives (22). Contraceptive use is very substantive in preventing unwanted pregnancies, unsafe abortions, and abortion- related complications, which expose adolescents to health related risks such as infertility and in some cases death (22). Globally,

contraceptive use in regions such as Europe and North America exceeds 70%, whereas usage in Western and Central Africa remains below 25% , illustrating a wide gap in access and utilization (23).

Many studies on adolescent sexual behaviour in the Ghanaian context have predominantly focused on adolescent girls and young women (18,24–26). Additionally, studies that included all adolescents have primarily focused on those in late adolescence (22,27,28), failing to capture the full range of adolescent experiences. Notably, research specifically targeting adolescents in senior high schools is lacking. These students are typically at a developmental stage where sexual experimentation increases (29), yet, their access to credible sexual and reproductive health information is often limited, and in some cases, they are unable to utilize the available information appropriately(29).

Contraceptive use in Ghana remains low, as reported by multiple studies(30–32). A study by Mavis et al(30) revealed that 26.7% of late adolescents used contraceptives in a cross-sectional study. Similarly, Begetayinoral et al(31) also reported that 26.3% of women of reproductive age were using modern contraceptives. These findings are below the national acceptor rate, which is 33.8%(33). In addition, factors influencing contraceptive use among high school adolescents have not been adequately assessed. Addressing this issue is crucial not only for improving adolescent health outcomes but also for advancing economic growth and long-term national development. Understanding the factors that influence contraceptive use among adolescents is therefore essential, as existing literature provides important insights into the dynamics surrounding adolescent sexual and reproductive health(34).

Some studies have identified a wide range of factors associated with contraceptive use among adolescents(24,34,35). For instance, Michael et al(35), in a cross-sectional study among adolescent girls in some sub-Saharan countries, found that girls who had multiple children and prior knowledge of contraceptives were more likely to use them compared to those without such experiences. Similarly, a study by William et al(34) in Tanzania revealed that factors independently associated with contraceptive use included age, marital status, knowledge of contraceptives, cohabitation, and having multiple sexual partners. However, it is important to note that in the latter study, some participants were interviewed in the presence of their parents, which may have influenced their willingness to respond openly and honestly.

Therefore, given the sparse of literature on sexual activity and contraceptive use among adolescents in senior high schools, this study aims to assess the factors influencing contraceptive use within this population. Exploring the sexual behaviour and contraceptive use among this group is vital for informing interventions and policy reforms that will improve sexual and reproductive health.

2.0 Operational definitions

Independent variables were the sociodemographic variables (age, sex, class, religion, ethnicity, residential status [the person adolescent lives with], employment status of the person adolescent lives with) and sexual activity characteristics (relationship status, multiple sexual partners), and awareness of contraceptives. These were selected based on multiple existing literatures(18,24,34–36)

Adolescent: Individuals aged between 10-19 years, as defined by the World Health Organization (WHO), who are transitioning from childhood to adulthood and experiencing physical, hormonal, and emotional changes.

Sexual Activity refers to the engagement in any form of sexual intercourse (penile-vaginal), by adolescents. In this study, it includes individuals who reported having had sexual intercourse at least once.

Contraceptive Use: The use of any method (such as condoms, pills, IUDs, injectables, etc.) to prevent pregnancy and reduce the risk of sexually transmitted infections (STIs) during sexual intercourse. In this study, contraceptive use refers to adolescents who reported using any form of contraception.

3.0 Materials and methods

3.1 Study design

A descriptive cross-sectional study was deemed appropriate as the researchers aimed to collect data at a specific point in time and describe the characteristics of adolescents concerning sexual activity and contraceptive use(37).

3.2 Study setting

The study was conducted within a senior high school in Ejisu municipality in the Ashanti region of Ghana, which constitutes both rural and urban localities(38). In the Ghanaian context, senior high school is a second-cycle institution that students attend after one graduate from Junior high school, typically covering grades equivalent to 10 through 12 in the international education system This school was selected as the study setting due to its

diverse student population and its ability to cater for students from various economic backgrounds. The school is a public mixed school which admits day students and also has boarding facilities for a section of the student body. The school offers five (5) programmes which include; Business, Home Economics, Visual Arts, General Arts and General Science. Facilities within the school include classrooms, administrative offices, a library, laboratories and outdoor spaces such as playgrounds and sports fields. This school was purposively selected for its unique representation of both urban and rural student populations. The school's status as a large, public, mixed-gender institution offering a wide range of academic programs made it an ideal microcosm for exploring adolescent sexual and reproductive behaviors in the municipality. Additionally, logistical feasibility and the willingness of the school management to approve the study were also factors in its selection.

3.3 Study population

Between 16/08/2022 and 15/09/2022, adolescents of both genders in senior high school were selected for the survey. The actual age range of participants in this study was 11 to 19 years. However, for the purposes of analysis and discussion, participants were categorized into early adolescence (10–14 years) and late adolescence (15–19 years), based on the WHO classification(39). SHS1 students were excluded from the study as they were on vacation during the data collection period. As a result, only SHS 2 and SHS 3 students were included in the study.

3.4 Inclusion and exclusion criteria

Adolescents aged 10 to 19 who were present in school at the time of the study and could speak, understand, and write English were eligible to participate. Sections of students who

were on vacation, suspension, critically ill, or had mental impairments were excluded from the study.

3.5 Sample size determination and sampling

A sample size of 330 was determined using the Slovin's formula:

$n = \frac{N}{1+N(e)^2}$ (40), and with a confidence interval of 95% (at a Z-score of 1.96), a margin of error of 5%, where **n** signifies the sample size, **N** signifies the population under study and **e** signifies the margin of error (0.05). The total population was 1210 using the total enrollment of students at the time of the study obtained from the school's administrative office. The minimum required sample size obtained was 300. A 10% non-response rate was considered to compensate for a non- response, resulting in a total estimated sample size of 330.

3.6 Data collection Tool

A structured questionnaire was used in this study, developed based on current literature and tailored to the study context. The questionnaire was pretested to ensure clarity and relevance. It was divided into six sections; the first section was on sociodemographic characteristics of adolescents (7 items). This section included questions on age, gender, class, religion, ethnicity, residential status (i.e., whom the adolescent lives with), and the employment status of the person the adolescent stays with. Second section focused on sexual activity (8 items). This section covers topics such as whether the respondent has a boyfriend or girlfriend, the age of their sexual partner, whether they have ever had sex, the number of sexual partners, the age of first sexual intercourse, how the first sexual experience occurred (whether they were coaxed, forced, or it was their own will), and whether they feel pressured

to have sex. Section three was awareness of contraceptives (11 items). Questions in this section included whether the respondent has heard of modern contraceptives, their source of information, the methods of contraception they were aware of, where one can access contraceptives, as well as similar inquiries regarding emergency contraceptives. The fourth section examined contraceptive use (11 items). This section included questions on whether the respondent had ever used contraceptives, which methods they had used, whether they used contraceptives every time they had sex, and the reasons for using them, such as "to avoid teenage pregnancy" or "to prevent STIs." Other questions included whether they used contraceptives during their first sexual intercourse, where they accessed contraceptives, and how frequently they used them, with options like "every time I have sex," "only during my first sexual intercourse," or "once in a while." The fifth section was on attitudes of adolescents towards contraceptives (7 Likert scale items). This section included statements like "I approve of adolescents using contraceptives every time they have sex." Higher scores reflect a positive attitude. Other statements, presented in a negative form and were reverse-coded, included: "Using contraceptives before a girl's first birth can lead to infertility," "Sex is not enjoyable when I use a condom," and "People who insist on condom use are promiscuous." Sixth section was barriers to contraceptive use among adolescents (8 items). Respondents were asked to indicate whether certain factors were barriers to their contraceptive use. These factors included religious beliefs, the attitude of providers, fear of side effects, embarrassment when buying contraceptives, fear of being seen by parents, partner disapproval, unavailability of contraceptives, and cultural beliefs.

3.7 Data collection procedure

A structured questionnaire was administered to 330 adolescents attending a Senior High School. A simple random sampling technique was employed to ensure an unbiased selection of participants(41). The researchers first obtained permission from the school administration, and students were briefed on the purpose of the study before participation. Participants were asked to choose between sealed opaque folded papers with an equal number labelled 'yes' and 'no'. Those who selected 'yes' after providing informed consent were included in the study.

To minimize potential bias, teachers were asked to leave the classrooms during the questionnaire administration to ensure participants did not feel intimidated or coerced. The questionnaire, comprising 45 questions, was distributed to participants, and they were given a maximum of one hour to complete it. Data was collected in all classes simultaneously to prevent students from discussing the questionnaire’s content beforehand. After collection, the completed questionnaires were reviewed by the research team to ensure completeness and accuracy before analysis.

3.8 Measurement of variables

Sexual activity, awareness of contraceptives and contraceptive usage

Sexual activity, awareness of contraceptives, and contraceptive use were measured using structured questions with binary response options (Yes/No). The percentage of participants who responded “Yes” was used to describe prevalence of each variable.

Attitudes toward contraception

Attitudes toward contraceptive use were assessed using a seven-item tool graded on a five-point Likert-scale (1 = Strongly Disagree, 2 = Disagree, 3 = Neutral, 4 = Agree, and 5 = Strongly Agree). Higher scores indicated more positive attitudes toward contraceptive use. Three of the items were phrased negatively ("Using contraceptives before a girl's first birth can lead to infertility," "Sex is not enjoyable when I use a condom," and "People who insist on condom use are promiscuous") and were reverse-coded before analysis so that higher scores consistently reflected a positive attitude. The total attitude score was then calculated by summing the responses to all seven items, with a possible range of 7 to 35. Respondents with total scores above the median were classified as having a positive attitude, while those scoring below the median were considered to have a negative attitude toward contraceptive use.

3.9 Data processing and analysis

The researchers confirmed the completeness of all collected questionnaires by reviewing each section, ensuring there were no missing data. Serial numbers were assigned to the questionnaire and were entered into Microsoft Excel for data cleaning and transferred to Statistical Product and Service Solutions (SPSS) version 22.0 for Windows (IBM SPSS Statistics) for the data to be analysed. Descriptive statistics (frequencies and percentages) were used to describe the categorical characteristics of participants.

To identify the factors associated with contraceptive use, first chi-square test was done to assess the association between the independent variables and the dependent variable. The ones that were found to be significant were further analyzed using binary logistic regression.

In model 1, univariate binary logistic regression was performed for each significant variable from the chi-square tests to calculate crude odds ratios (OR).

Model 2 was a multivariable binary logistic regression that included all independent variables (relationship status, multiple sexual partners, and awareness of contraceptives) found to be significant in Model 1, while adjusting for control variables such as age, sex, class, religion, and residential status. Adjusted odds ratios (AOR) and 95% confidence intervals (CI) were reported. Statistical significance was set at $p \leq 0.05$.

3.10 Quality assurance; Validity

To ensure content validity, we invited five public health experts to assess the relevance of each question to its corresponding factor through a face-to-face approach. This process helped determine whether the questionnaire accurately measured the intended information. Minor adjustments were made to some questions by all five experts to enhance their cultural relevance and acceptability to the study participants.

3.11 Ethical considerations

Prior to the conduct of the study, an institutional-based approval was sought from the management of the school in order to conduct the research in the school. Ethical approval, referenced as CHRPE/AP/426/22, was granted by the Committee on Human Research, Publication, and Ethics (CHRPE) at the School of Medicine and Dentistry, Kwame Nkrumah University of Science and Technology in Kumasi, Ghana. Since majority of the participants resided in the school's boarding house and many parents lived far from the school, direct consent from parents was not feasible. Therefore, verbal consent was obtained from the school authorities acting in loco parentis due to the boarding school arrangement for the

participation of the students. The participants were briefed on the research's objectives before seeking their consent to participate. They were provided with an opportunity to inquire about the study procedures. Verbal consent was documented by the research team and witnessed by the teachers who acted as custodians in the school setting. Participation was entirely voluntary, with no direct benefits to participants, and they had the freedom to withdraw at any point. To protect anonymity, no names or other identifying details were obtained.

4.0 RESULTS

4.1 Sociodemographic characteristics of adolescents

Majority of the participants were in their late adolescents' stage (n=290, 87.9%), and were females (n=196, 59.4%). Most of the participants lived with both parents (n=194, 58.8%), and were Christians (90.9%, n=300) ***(Table 1)*****.**

Table 1. Sociodemographic characteristics of adolescents

Variable	Frequency	Percentage (%)
**Age**		
10-14	40	12.1
15-19	290	87.9
**Sex**		
Male	134	40.6
Female	196	59.4

**Class**		
SHS Level 2	190	57.6
SHS Level 3	140	42.4
**Religion**		
Christian	300	90.9
Muslim	30	9.1
**Ethnicity**		
Akan	272	82.4
Ga-Adangbe	12	3.6
Ewe	18	5.5
Others (Dagoba, Mamprusi, Fafra)	28	8.5
**Residential status**		
Living with both Parent	194	58.8
Living with Single parent	120	36.4
Living alone	16	4.8
**Employment status [of the person adolescent lives with]**		
Unemployed	129	39.1
Employed	201	60.9

4.2 Sexual activity among adolescents

Half of the participants (n=166, 50.3%) had boyfriends or girlfriends and majority had multiple sexual partners (n=64, 50.2%). Additionally, over a-third of participants (n=126,

38.2%) have ever had sex, and of this, more than half (n=65, 51.6%) experienced their first sexual intercourse during early adolescence, between the ages of 10 and 14 years. Participants who engaged in sex on their own will were (n= 87, 69%). Furthermore, 119 (36.1%) of the participants reported feeling pressured to have sex, predominantly from friends (n=70, 58.8%) ***(Table 2).***

Table 2. Sexual Activity among Adolescents

Variable	Frequency	Percentage (%)
**Having a Boyfriend/Girlfriend**		
Yes	166	50.3
No	164	49.7
**Age of boyfriend/girlfriend (N=166)**		
10-19	108	65.0
More than 19	58	35.0
**Ever had sex**		
Yes	126	38.2
No	204	61.8
**Do you have multiple partners**		
Yes	65	19.7

No	265	
		80.3
**Number of sexual partners (N=126)**		
1	62	49.2
2	24	19.0
3	12	9.6
More than 3	28	22.2
**Age of first Sexual Intercourse**		
10-14	65	51.6
15-19	61	48.4
**How first sexual intercourse happened (N=126)**		
Own will	84	66.7
Coaxed	17	13.5
Forced	15	11.9
**Pressure to have sex**		
Yes	119	36.1
No	211	63.9

**Sources of pressure to have sex (N=119)**		
Friends	70	58.8
Relatives	12	10.0
Teachers	10	8.4
Boyfriend/Girlfriend	27	22.7

4.3 Awareness of contraceptives among Adolescents

Majority of the participants (n=283, 85.8%) surveyed were aware of contraceptive methods. Study results indicated that (n=155, 54.8%) were aware of male condoms; the least (n=12, 4.20%) were aware of IUD. Among those who knew about the contraceptive methods, television (n=144, 50.9%) and peers (n=113, 39.9%) were the major sources of their information and the least were parents (n=10, 5.4%). On whether modern contraceptives provide 100% protection from pregnancy, a notable number of participants, 117(35.5%) reported that they did not know ***(Table 3).***

Table 3. Awareness of contraceptives among Adolescents

**Variables**	**Frequency**	**Percentage (%)**
**Heard of contraceptives**		
Yes	283	85.8
No	47	14.2

**Heard of any method of contraceptive**		
Yes	283	85.8
No	47	14.2
**Where did you hear it from (N=283)**		
Radio	105	37.1
Television	144	50.9
Peers	113	39.9
Parent	62	21.9
Teacher	81	28.6
Health worker	79	27.9
Internet	113	39.9
**What method of contraception do you know (N=283)**		
Male condom	155	54.8
Abstinence	35	12.4
Implant	18	6.4
Withdrawal method	44	15.5
Female condom	40	14.1
IUD	12	4.2

Urinating after sex	30	10.6
Injectable	20	7.1
Pills	96	33.9
**Heard about emergency contraceptive**		
Yes	185	56.1
No	145	43.9
**If yes, where did you hear about this emergency contraceptive from (N=185)**		
Radio	88	47.6
Television	103	55.7
Peers	74	40
Health worker	73	39.5
Teacher	26	14.1
Internet	73	39.5
Parents	10	5.4
**How often do you know emergency contraceptives can be used within a year (N=185)**		

Once a year	40	21.6
Twice a year	48	26.5
Three times a year	22	11.4
More than three times a year	75	40.5
**Do you know where you can access contraceptive**		
Yes	249	75.5
No	40	12.1
Unanswered	41	12.42
**If yes, where (N=249)**		
Pharmacy	194	58.8
Friend	51	15.5
Hospital or clinic	208	63.0
Health personnel	68	20.6
**Can a girl become pregnant from just one sexual intercourse**		
Yes	240	72.7
No	29	8.8
I don’t know	61	18.5

**Do contraceptives provide 100% protection from pregnancy**		
Yes	79	23.9
No	134	40.6
I don't know	117	35.5

4.4 Use of contraceptives among adolescents

Among the participants who have ever had sex, 100 (79.4%) had ever used contraceptives. Condoms (n=66, 66%) and pills (n=43, 43%) were the most common methods of contraception. Contraceptive use during the first sexual encounter was reported by 67 (67%). The majority of the participants used contraceptives to avoid pregnancy (n=58, 58.0%). Regarding the frequency of emergency contraceptive usage, 20 (42.3%) indicated they use it every time they have sex ***(Table 4)*.**

Table 4. Use of contraceptive among adolescents who have had sex

**Variable**	**Frequency**	**Percentage (%)**
**Use of Contraceptives(N=126)**		
Yes	100	79.4
No	26	20.6

**Method of Contraceptive used (N=100)**		
Condom	66	66.0
Pills	43	43.0
IUD	2	2.0
Implants	3	3.0
Injectable	3	3.0
Withdrawal	14	14.0
Safe Period	23	23.0
**Do you use contraceptive anytime you have sex**		
Yes	50	50.0
No	50	50.0
**Reasons for using contraceptives (N=100)**		
To avoid teenage pregnancy	58	58.0
To prevent STI	42	42.0
**Discuss Contraceptive use with your partner(N=100)**		
Yes	59	59.0
No	31	31.0
I do not remember	10	10.00

**Use of contraceptive first time you have sex**		
Yes	67	67.0
No	33	33.0
**Method of Contraceptive use first time of having sex (N=67)**		
Condom	46	68.7
Pills	32	47.8
Implants	3	4.5
IUD	1	1.5
Withdrawal	12	17.9
Safe Period	18	26.7
Injectable	1	1.5
**Access to Contraceptive(s) (N=67)**		
Hospital	16	23.9
Pharmacy	49	73.1
Friends	22	32.8
Health Personnel	7	10.4

**Frequency of Contraceptive use (N=100)**		
Every time I have sex	50	50.0
Only on my first sexual intercourse	7	7.0
Once a while	43	43.0
**Use of Emergency Contraceptives(N=75)**		
Yes	47	62.7
No	28	37.3
**Frequency of Emergency Contraceptive use (N=47)**		
Every time I have sex	20	42.5
Once a year	10	21.3
Twice a year	9	19.1
Three times a year	4	8.5
More than three times a year	4	8.5

4.5 Attitude Towards Contraceptive Use

Participants were asked to indicate their level of agreement to seven statements on contraceptive use, using a five-point Likert scale rating of strongly agree=5, agree=4,

uncertain=3, disagree=2 and strongly disagree=1. All the negative worded statements were reverse-coded. The minimum, maximum and median scores were 7, 35 and 18 respectively; participants whose overall scores were above the median score (18) were said to have a positive overall attitude towards contraceptive use. Overall, 76.7% of the adolescents were found to have a positive attitude towards contraceptive use ***(Figure 1).***

Responses Regarding Attitude of Adolescents Towards Contraceptive Use

From the survey, majority (n= 80, 24.2%) agree with the usage of contraceptives by adolescents. Most of the participants (n=129, 39.1%) were uncertain that the use of contraceptives by a girl before her first birth can lead to infertility. Approximately, 117(35%) of the respondents were not certain as to whether "sex is not enjoyable when I use condom". Participants whose religion was against usage of contraceptives were (n=141, 42.7%), and 207(62.7%) reported contraceptive use is not only the responsibility of females ***(Table 5)****.*

Table 5. Attitude of adolescents towards contraceptive use

**Variable**	**Frequency**				
	**Strongly disagree**	**Disagree**	**Uncertain**	**Agree**	**Strongly agree**
I approve the use of contraceptive by adolescents anytime they have sex [preferred]	53(16.1%)	73(22.1%)	58(17.6%)	80(24.2%)	66(20.0%)

Use of contraceptive by a girl before her first birth can lead to infertility	35(10.6%)	46(13.9%)	129(39.1%)	73(22.1%)	47(14.2%)
Sex is not enjoyable when I use condom	51(15.5%)	30(9.1%)	117(35.3%)	71(21.5%)	61(18.5%)
People who insist on condom use are promiscuous	44(13.3%)	68(20.6%)	86(26.1%)	68(20.6%)	64(19.4%)
Contraception usage is against my religion	59(17.9)	75(22.7%)	55(16.7%)	74(22.4%)	67(20.3%)
I will use contraception in the future	96(29.1%)	79(23.9%)	79(23.9%)	47(14.2%)	29(8.8%)
Contraception is the responsibility of only females	125(37.9%)	82(24.8%)	51(15.5%)	42(12.7)	30(9.1%)

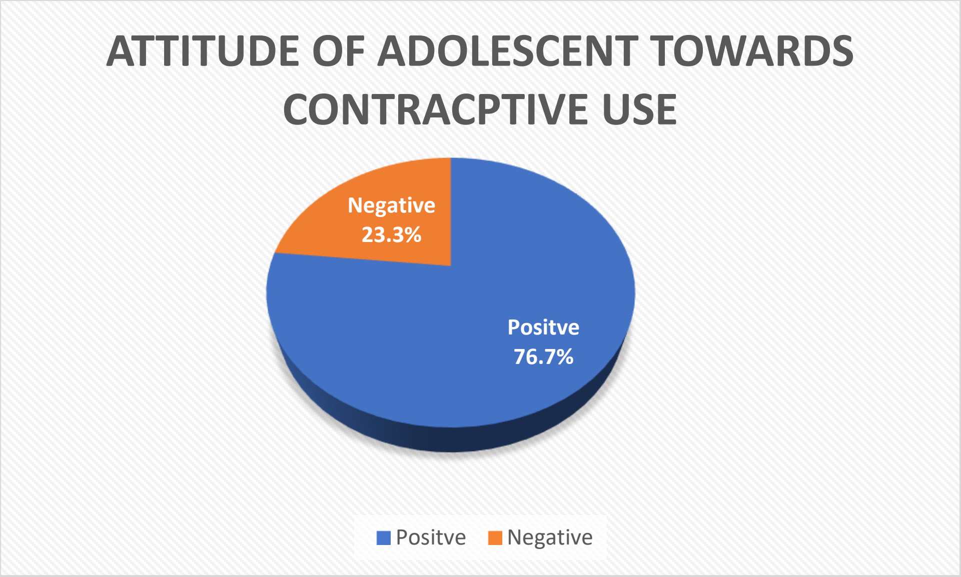

Fig 1. Attitude of Adolescent towards contraceptive use.

4.6 Association between socio-demographic characteristics of Adolescents and use of contraceptives

The study found that having a boyfriend or girlfriend, the number of sexual partners an adolescent had, and awareness of modern contraceptives (all with a p-value of $p \le 0.001$) were statistically significant factors influencing contraceptive use. There was no significant association between some of the sociodemographic characteristics (age, sex, class, religion, ethnicity, residential status, and employment status of the person adolescent lives with) ***(Table 6).***

Table 6. Association between socio-demographic characteristics of Adolescents and use of contraceptives

Variables	Usage of contraceptive	Chi-square	Total

	Yes, (%)	No, (%)	(p-value)	(N)
Age				330
14-16	20.0	80.0	0.309	40
17-19	31.7	68.3		290
Sex				330
Male	30.6	69.4	0.700	134
Female	30.1	69.9		196
Class				330
SHS 2	29.8	70.2	0.666	191
SHS 3	30.9	69.1		139
Religion				330
Christian	29.3	70.7	0.220	300
Muslim	40.0	60.0		30
Ethnicity				330
Akan	31.7	68.3	0.887	243
Fante	20.7	79.3		29
Ga-Adangbe	41.7	58.3		12
Ewe	33.3	66.7		18
Others (Dagoba, mamprusi, Fafra)	25.9	74.1		28
Residential status				330
Both parent	26.3	73.7	0.072	194

Single parent	34.1	65.9		120
Live alone	50.0	50.0		16
Employment status				330
Unemployed	31.8	60.2	0.920	129
Employed	29.4	70.6		201
Do you have a boyfriend/girlfriend				330
Yes	52.4	47.6	≤0.001*	166
No	7.9	92.1		164
Number of sexual partners				330
0	0.0	100.0	≤0.001*	204
1	71.0	29.0		62
2	75.0	25.0		24
3	66.7	33.3		12
More than 3	89.2	10.8		28
Heard of modern contraceptive				330
Yes	65.7	34.3	≤0.001*	283
No	6.3	93.7		47

4.7 Logistic regression predicting modern contraceptive use

Age, residential status, religion, class, and gender were included as control variables, however, none of the control variables significantly predicted contraceptive use. The independent variables, whether the respondent had a boyfriend or girlfriend, the number of sexual partners, and whether the respondent had heard of modern contraceptives were statistically significant predictors in the model. Adolescents who had heard of modern contraceptives were 4.655 times more likely to use them compared to those who had not (AOR = 4.655, 95% CI = 1.395–15.530, p = 0.012). Similarly, those with multiple sexual partners were significantly more likely to use contraceptives than those without (AOR = 9.518, 95% CI = 4.320–20.969, p < 0.001). Additionally, having a boyfriend or girlfriend was associated with approximately seven times higher odds of contraceptive use (AOR = 7.208, 95% CI = 3.533–14.706, p < 0.001). These associations remained significant after adjusting for the control variables in the model ***(Table 8).***

Table 7. Logistic regression predicting modern contraceptive use

**Variable**	**B(Coefficient)**	**Adjusted Odds Ratio (EXP(B))**	**95%CL**	**p-value**
Age (years)				
10-14years	0.216	1.241	0.464-3.322	0.667
15-19years**(ref)**				
Sex				
Male	0.017	1.017	0.505-2.047	0.963

Female**(ref)**				
Religion				
Christian	-0.240	0.787	0.283-2.183	0.645
Muslim**(ref)**				
Residential status				
Both parents	-0.147	0.863	0.206-3.620	0.841
Single parent	0.091	1.095	0.255-4.697	0.902
Live alone**(ref)**				
Heard of modern contraceptive				
Yes	1.538	4.655	1.395-15.530	0.012*
No**(ref)**				
Do you have multiple partners				
Yes	2.253	9.518	4.320-20.969	< 0.001*
No**(ref)**				
Do you have boyfriend/girldriend				
Yes	1.975	7.208	3.533-14.706	< 0.001*
No**(ref)**				
Class				
SHS 2	-0.380	0.684	0.344-1.359	0.278

SHS 3**(ref)**

5.0 Discussion

This study aimed to assess sexual activity and contraceptive use among adolescents. Findings from the study showed that a little over half of adolescents were in a relationship with someone of the opposite sex. This corroborates findings from other studies, which have shown that the majority of adolescents have been in a relationship with someone of the opposite sex (36,42).

Furthermore, the study found that among adolescents who had engaged sexual activity, the majority had their sexual debut during early adolescence stage (10-14 years). This may be explained by the exploration and heightened sexual curiosity that occur among adolescents during their critical growth phase of identity formation. A study conducted in Kenya reported that adolescents who initiate sex at an early stage are influenced by peer pressure, as well as easy access to phones and the internet(43). This early exposure may be due to increased exposure to erotic materials (nudity, photographs/videos of sexual activity), which are readily available. This increases their curiosity and finally commit to the act of sexual activity. This is further supported by the findings of this current study, which revealed that adolescents mostly feel pressured by their friends and their boyfriend or girlfriend to have sex.

The study also reported high percentage of adolescents having multiple sexual partners. This is consistent with the findings of Attibu (42) who reported that nearly half of the adolescent

in the study had more than one sexual partner. This can be an indicator of increased risk for pregnancy, unsafe abortion, and STIs, implying that adolescents may not maintain long-term sexual relationships but instead enter new relationships due to unmet expectations, such as financial rewards or other benefits (44).

The findings from this study revealed that awareness of contraceptive was high among adolescents. This is in keeping with a study conducted by Agbanyo(17),which revealed that 88% of adolescents were aware of contraceptives. From our findings, 86.1% of adolescents who had heard about contraception were able to mention at least one type of contraceptive and where it could be accessed. This high awareness of contraception may be attributed to recent change in social media advertisement which includes both video and audio content.

In this study, the most frequently mentioned sources of information on contraceptives were television, peers and radio which is similar to findings from research conducted in Tanzania (45). Additionally, a study by Hagan and Buxton indicated that, majority (60.0%) of adolescents reported receiving information through the radio or television(46). The least source of information on contraceptives use was found to be from parents. This is in agreement with a study conducted by Hagan and Buxton, which also revealed that, only 3.3% of adolescents received information on contraceptive from parents(46). This could be as a result of African cultural setting where discussing sexual issues at home is often considered immoral behaviour (47). As a results, both adolescents and adults may feel uncomfortable discussion topics related to sexuality and contraceptive use. The source of information is a major factor in providing adolescents with correct information regarding contraceptives and

their use. A reliable source of information from health care providers and mass media provide adolescents with more accurate information than friends (45).

Findings from the current study revealed a high prevalence of contraceptive use among adolescents, which exceeds the Ghana Health Service's national family planning target of 33.8%(33), and is higher than what has been reported in other studies conducted among adolescents(34,35). This is in agreement with findings from a study by Maness et al, which also showed high usage of contraceptives by adolescents(48). However, this is inconsistent with other studies which revealed low use of contraceptives among adolescents(34). For instance, findings from a study by Kinaro et al (49) revealed that only 8.6% of all adolescents had ever used a contraceptive method. The high rate of contraceptive use in the present study may probably be attributed to the high level of awareness as observed among participants.

Condom and Pills were the most commonly used contraceptives by adolescents. Condoms were the most frequently used method of contraception probably because it is the most popular, easily accessible, affordable, and have dual protection against teenage pregnancy and sexually transmitted diseases (50). The majority of adolescents accessed contraceptives from the pharmacy and peers, which is in line with the findings of Kara et al(45). Adolescents tend to prefer to obtaining condoms and pills from pharmacies, likely because it is easily accessible in almost every community as compared to hospitals. This corroborates with a study conducted by Chandra-Mouli and Akwara, which revealed that pharmacies offer adolescents accessible locations, longer operating hours, and most importantly, anonymity in obtaining contraceptives(51). The study further indicated that the preference for

pharmacies was as a result of difficulty in obtaining contraceptives from health facilities due to restrictive laws, health worker bias, or privacy concerns

Adolescents exhibited positive attitudes towards contraceptive use. The present study reported that majority of adolescents approved the “use contraceptive anytime they have sex”. These findings contrast with those of a previous study by Sam, where the level of awareness and attitude towards contraceptives use among adolescents were found to be very low and only few of them have ever used or currently using effective contraceptive methods(52).

However, a few participants exhibited negative attitudes towards contraceptive use. For instance, most of the adolescents in this study supported the idea that contraceptive use by a girl before her first birth can lead to infertility. This is in agreement with a study conducted by Sam, where 67.0% of respondents strongly agreed that a girl using contraceptives before having her first child could cause infertility(52). This is also consistent with Lauren et al, who found that future fertility was among the main attitudes which tend to be an obstacle to contraceptives use(53). Adolescents have a misconception about contraceptive use, possibly because much of the information they receive comes from unreliable sources like friends who lacked in-depth knowledge. Additionally, concerns may also be as a result of the side effects of contraceptives (especially oral pills and emergency contraceptives) on the menstrual cycle of female adolescents.

With regard to contraceptive use, awareness, number of sexual partners, and having a boyfriend or girlfriend were found to influence adolescents' use of contraceptives. This

finding contrasts with a study conducted in Nigeria by Abdul et al among university students, which found no association between awareness and contraceptive use(54).

Research conducted by James et al. showed a significant association between the number of sexual partners and contraceptive use(55). Adolescents with two or more sexual partners are more likely to engage in risky sexual behaviors (56). To prevent unintended pregnancies, STIs, school dropout, and unsafe abortions, they often resort to contraceptive use. This aligns with the findings of the current study, which adolescents with multiple partners were found to be nine times more likely to use contraceptives. Additionally, all adolescents who had ever used contraceptives indicated that their primary reason was either to prevent unwanted pregnancy or prevent STIs.

Being in long-term or casual relationships is also significantly associated with effective contraceptive use (36). This finding is consistent with the results of the current study, which showed that having a boyfriend or girlfriend increased an adolescent's likelihood of using contraceptives by approximately seven times.

6.0 Conclusion and recommendation

Based on the findings of this study, it is evident that adolescent sexual activity and contraceptive use are significant issues that require attention and targeted interventions. The prevalence of multiple sexual partners and early sex debut among adolescents reflects potential risks such as unintended pregnancies, unsafe abortions, and sexually transmitted infections (STIs). On a positive note, there is a high level of awareness and positive attitudes towards contraceptive use among adolescents. However, misconceptions persist, potentially due to unreliable sources of information. Given this, healthcare providers and other relevant

stakeholders should use multidimensional and individualized interventions to improve the reproductive health of adolescents.

Parents and guardians should be encouraged to educate adolescents about sexual activity and contraceptive use. Collaboration between the Ministry of Health and the Ministry of Education is essential to organize regular health seminars to keep adolescents well-informed about issues related to sexuality and contraception. Additionally, there is a need to reconsider incorporating comprehensive sex education starting at the basic school level. Peer educators should be identified and trained to provide sexual education, as adolescents often feel more comfortable seeking information from their peers. Finally, future studies should explore the role of parents and guardians in influencing adolescents' sexual activity and contraceptive use.

Efforts should be directed towards providing training to health workers and educators on how to effectively communicate accurate, evidence-based information to parents and the general public. This can include workshops on health communication and the use of trusted sources.

7.0 Limitations of the study

The use of self-reporting and the school environment may have influenced participants to provide responses that they perceived as socially desirable. In addition, the researchers used a small sample size. However, the findings of the current research have relevant implications for improving adolescent sexual and reproductive health. Future research should prioritize assessing sexual activity and contraceptive use beyond the school setting.

8.0 Acknowledgements

The authors sincerely thank the management of the school for granting us the opportunity to conduct this study in their esteemed institution. We also extend our heartfelt gratitude to the students who participated in this study.

References

1. Jing Z, Li J, Wang Y, Zhou C. Prevalence and Trends of Sexual Behaviors Among Young Adolescents Aged 12 Years to 15 Years in Low and Middle-Income Countries: Population-Based Study. JMIR Public Heal Surveill. 2023;9.

2. Alhassan AR, Wepeba TW, Abdulai K, Iddrisu R, Aninanya GA. Prevalence and Associated Factors of Adolescent (15-19 Years) Childbearing in Ghana. Biomed Res Int. 2024;2024.

3. WHO. Adolescent pregnancy fact sheet. Adolesc Pregnancy Fact Sheet [Internet]. 2020;1. Available from: https://www.who.int/news-room/fact-sheets/detail/adolescent-pregnancy

4. Ndahimana R, Umutoni NJ, Nteziryayo JP, Semafara S, Binayisa G, Ishimwe J, et al. Prevalence, Trends in and Determinants of Teenage Pregnancies in Rwanda: Analysis of Rwanda Demographic and Health Survey (2010 to 2020). J Biomed Res Environ Sci. 2023;4(10):1425–34.

5. WHO. Ghana Contraception within the context of adolescents' sexual and reproductive lives: Country profile Microsoft Word - UGANDA. 2021;5:9–11.

Available from: who.int

6. Vijayakumar N, Op de Macks Z, Shirtcliff EA, Pfeifer JH. Puberty and the human brain: Insights into adolescent development. Neurosci Biobehav Rev [Internet]. 2018;92(January):417–36. Available from: https://doi.org/10.1016/j.neubiorev.2018.06.004

7. Kyei-Arthur F, Agyekum MW, Kyei-Gyamfi S. "You cannot stay with one person once you begin having sex at a young age": the prevalence, correlates and effects of early sexual debut among children in Ghana. Reprod Health [Internet]. 2024;21(1):1–10. Available from: https://doi.org/10.1186/s12978-024-01775-4

8. Pringle J, Mills KL, McAteer J, Jepson R, Hogg E, Anand N, et al. The physiology of adolescent sexual behaviour: A systematic review. Cogent Soc Sci. 2017;3(1):1–14.

9. Kyilleh JM, Tabong PTN, Konlaan BB. Adolescents' reproductive health knowledge, choices and factors affecting reproductive health choices: A qualitative study in the West Gonja District in Northern region, Ghana. BMC Int Health Hum Rights. 2018;18(1):1–12.

10. WHO. Adolescent health. World Heal Organ. 2020;83–108.

11. Wondimagegne YA, Debelew GT, Koricha ZB. Barriers to contraceptive use among secondary school adolescents in Gedeo zone, South Ethiopia: A formative qualitative study. BMJ Open. 2023;13(3):1–10.

12. Masonbrink AR, Hurley EA, Schuetz N, Rodean J, Rupe E, Lewis K, et al. Sexual behaviors, contraception use and barriers among adolescents and young adults in

rural Haiti. BMC Womens Health. 2023;23(1):1–10.

13. Dandala S, Hodambia M. Journal Wetenskap Health. J Wet Heal [Internet]. 2021;4(4):22–7. Available from: https://pdfs.semanticscholar.org/dc01/0a49f45e1d6d020306d577378e24a2bd59ea.pdf

14. James PB, Osborne A, Babawo LS, Bah AJ, Margao EK. The use of condoms and other birth control methods among sexually active school-going adolescents in nine sub-Saharan African countries. BMC Public Health [Internet]. 2022;22(1):1–11. Available from: https://doi.org/10.1186/s12889-022-14855-6

15. Devi P. Sexual and Reproductive Health Behaviour of Adolescents-A Survey article. 2016;III(10):10918–29. Available from: www.euacademic.org

16. Etrawati F, Yeni Y, Lionita W, Rahmawaty A, Fajarningtiyas DN. Sexual Risk Behavior and its Impact on Unwanted Pregnancy Among Adolescents. J Ilmu Kesehat Masy. 2024;14(3):319–21.

17. Agbanyo Ri. KNOWLEDGE AND BARRIERS TO CONTRACEPTIVE USE AMONG IN-SCHHOL ADOLESCENT IN ADAKLU DISTRICT. 2018;(10637685):1–74.

18. Amoah EJ, Hinneh T, Aklie R. Determinants and prevalence of modern contraceptive use among sexually active female youth in the Berekum East Municipality, Ghana. PLoS One [Internet]. 2023;18(6 JUNE):1–18. Available from: http://dx.doi.org/10.1371/journal.pone.0286585

19. Beson P, Appiah R, Adomah-Afari A. Modern contraceptive use among reproductive-

aged women in Ghana: Prevalence, predictors, and policy implications. BMC Womens Health. 2018;18(1):1–8.

20. Alidou S, Dahourou LD, Dah TTE, Sogo A, Kenao TS, Yehadji D, et al. Unmet needs for modern contraceptive methods among sexually active adolescents and young women in Togo: a nationwide cross-sectional study. Front Public Heal. 2023;11(July).

21. Atiglo DY, Biney AAE. Correlates of sexual inactivity and met need for contraceptives among young women in Ghana. BMC Womens Health. 2018;18(1):1–10.

22. Mahama E, Owusu-agyei S. Use of contraceptives among adolescents in Kintampo , Ghana : a cross-sectional study. 2014;7–15.

23. WHO. Contraception Evidence brief. 2019;(3).

24. Agyemang J, Newton S, Nkrumah I, Tsoka-Gwegweni JM, Cumber SN. Contraceptive use and associated factors among sexually active female adolescents in Atwima Kwanwoma District, Ashanti region-Ghana. Pan Afr Med J. 2019;32:1–13.

25. Sumankuuro J, Asuuri A, Mikare MD, Ngmenkpieo F, Crockett J, Wulifan JK. High School Student's Knowledge, Attitude and Participation in Sexual Health Education in Rural Northern Ghana. J Biosci Med. 2020;08(05):64–83.

26. Keogh SC, Otupiri E, Castillo PW, Li NW, Apenkwa J, Polis CB. Contraceptive and abortion practices of young Ghanaian women aged 15–24: evidence from a nationally representative survey. Reprod Health [Internet]. 2021;18(1):1–17. Available from: https://doi.org/10.1186/s12978-021-01189-6

27. Oppong FB, Logo DD, Agbedra SY, Adomah AA, Amenyaglo S, Arhin- K, et al.

Determinants of contraceptive use among sexually active unmarried adolescent girls and young women aged 15 – 24 years in Ghana : a nationally representative cross- - sectional study. 2021;1–10.

28. Li Z, Patton G, Sabet F, Zhou Z, Subramanian S V., Lu C. Contraceptive Use in Adolescent Girls and Adult Women in Low- And Middle-Income Countries. JAMA Netw Open. 2020;3(2):1–15.

29. Muhlisa M, Amiruddin R, Moedjiono AI, Suriah S, Hadju V, Salmah U, et al. Risky reproductive health behavior and information access on adolescents: Mix-method analysis. Sci Eng Heal Stud. 2024;18:1–6.

30. Anyomi MA, Adjei JS, Anyomi BK. Knowledge , attitudes , and perceptions of contraceptive use among senior high school students in Hohoe , Ghana : Addressing barriers to adolescent sexual health . Knowledge , attitudes , and perceptions of contraceptive use among senior high school stude. 2025;(December 2024).

31. Lahole BK, Banga D, Mare KU. Modern contraceptive utilization among women of reproductive age in Ghana: a multilevel mixed-effect logistic regression model. Contracept Reprod Med. 2024;9(1).

32. Osborne A, Aboagye RG, Bangura C, Ahinkorah BO. Predictors of intention to use contraceptives among married and cohabiting women in Ghana: A cross-sectional study. Contracept Reprod Med. 2024;9(1).

33. Ministry of Health. 2021 Holistic Assessment Report. Minist Heal [Internet]. 2022;(April):56. Available from: https://www.moh.gov.gh/wp-

content/uploads/2022/09/2021-Holistic-Assessment-Report_v1.7.3.pdf

34. Nkenguye W, Ismail H, Urassa EP, Yongolo NM, Kagoye S, Msuya SE. Factors Associated with Modern Contraceptive Use Among Out of School Adolescent Girls in Majengo and Njoro Wards of Moshi Municipality, Tanzania. East African Heal Res J. 2023;7(1):32–9.

35. Michael TO, Ojo TF, Ijabadeniyi OA, Ibikunle MA, Oni JO, Agboola AA. Prevalence and factors associated with contraceptive use among sexually active adolescent girls in 25 sub-Saharan African countries. PLoS One [Internet]. 2024;19(2 February):1–15. Available from: http://dx.doi.org/10.1371/journal.pone.0297411

36. Upadhyay UD, Raifman S, Raine-Bennett T. Effects of relationship context on contraceptive use among young women. Contraception [Internet]. 2016;94(1):68–73. Available from: http://dx.doi.org/10.1016/j.contraception.2016.02.025

37. Wang X, Cheng Z. Cross-Sectional Studies: Strengths, Weaknesses, and Recommendations. Chest [Internet]. 2020;158(1):S65–71. Available from: https://doi.org/10.1016/j.chest.2020.03.012

38. Ghana Statistical Service. Ghana 2021 Population and Housing Census. 2021;6.

39. Bhargava M, Bhargava A, Ghate SD, Shyama Prasad Rao R. Nutritional status of Indian adolescents (15-19 years) from National Family Health Surveys 3 and 4: Revised estimates using WHO 2007 Growth reference. PLoS One [Internet]. 2020;15(6 June):1–24. Available from: http://dx.doi.org/10.1371/journal.pone.0234570

40. Slovin's. Slovin formula in research pdf. 1960;

41. Krishna R, Maithreyi R, Surapaneni KM. Research bias: A review for medical students. J Clin Diagnostic Res. 2010;4(2):2320–4.

42. Attibu RIE. Sexual Behaviour among Adolescents in Senior High Schools within the Tamale Metropolis, Northern Region of Ghana. 2015;(10268216):1–98.

43. Mauti G, Muthoni S. Early Sexual Debut and the Associated Factors among School Going Adolescents in Selected Schools in Kenya. East African Heal Res J. 2023;7(2):309–17.

44. Vincent G, Alemu FM. Factors contributing to, and effects of, teenage pregnancy in Juba. South Sudan Med J. 2016;9(2):28–31.

45. Kara WSK, Benedicto M, Mao J. Knowledge, Attitude, and Practice of Contraception Methods Among Female Undergraduates in Dodoma, Tanzania. Cureus. 2019;11(4).

46. Hagan JE, Buxton C. Contraceptive Knowledge, Perceptions and Use among Adolescents in Selected Senior High Schools in the Central Region of Ghana. J Sociol Res. 2012;3(2):170–80.

47. Khumalo S, Taylor M, Makusha T, Mabaso M. Intersectionality of cultural norms and sexual behaviours: a qualitative study of young Black male students at a university in KwaZulu-Natal, South Africa. Reprod Health. 2020;17(1):1–10.

48. Maness SB, Thompson EL, Lu Y. Associations Between Social Determinants of Health and Adolescent Contraceptive Use: An Analysis From the National Survey of Family Growth. Fam Community Heal. 2022;45(2):91–102.

49. Kinaro J, Kimani M, Ikamari L, Ayiemba EHO. Perceptions and Barriers to

Contraceptive Use among Adolescents Aged 15 - 19 Years in Kenya: A Case Study of Nairobi. Health (Irvine Calif). 2015;07(01):85–97.

50. Beksinska M, Wong R, Smit J. Male and female condoms: Their key role in pregnancy and STI/HIV prevention. Best Pract Res Clin Obstet Gynaecol. 2020;66:55–67.

51. Chandra-Mouli V, Akwara E, Engel D, Plessons M, Asnake M, Mehra S, et al. Progress in adolescent sexual and reproductive health and rights globally between 1990 and 2016: what progress has been made, what contributed to this, and what are the implications for the future? Sex Reprod Heal Matters [Internet]. 2020;28(1). Available from: https://doi.org/10.1080/26410397.2020.1741495

52. Sam NB. Knowledge and Awareness Level of Contraceptive Usage Among Adolescents in Mankranso of Ahafo-Ano South District. Biomed J Sci Tech Res. 2020;31(4).

53. Chernick S L, Orav EJ, Brennan TA, Burstin HR. in an Emergency Department. J Gen Intern Med. 1999;14(3):82–7.

54. Abdul I, Imohagene A, Nigeria LO… S of, 2018 undefined. Awareness, knowledge and contraceptive use among female students in. JesonOrgNg [Internet]. 2018;1(2). Available from: https://jeson.org.ng/index.php/jeson/article/view/17

55. James PB, Osborne A, Babawo LS, Bah AJ, Margao EK. The use of condoms and other birth control methods among sexually active school-going adolescents in nine sub-Saharan African countries. BMC Public Health. 2022;22(1):1–13.

56. Cruzeiro ALS, Souza LD de M, Silva RA da, Pinheiro RT, Rocha CLA da, Horta BL.

Comportamento sexual de risco: fatores associados ao número de parceiros sexuais e ao uso de preservativo em adolescentes. Cien Saude Colet. 2010;15(suppl 1):1149–58.

Supporting Information

S-1 File (DOCX)

S-2 File (DOCX)

S- 3 Data (XLS)

S-4 File (DOCX)

S-5 File (DOCX)

Figure
Click here to access/download;Figure;Figure.pdf

Lorem Ipsum

Lorem ipsum dolor sit amet, consectetur adipiscing elit. Mauris maximus fringilla ligula, in malesuada erat tempor ac. Quisque dapibus posuere turpis, vel aliquam massa vehicula non.

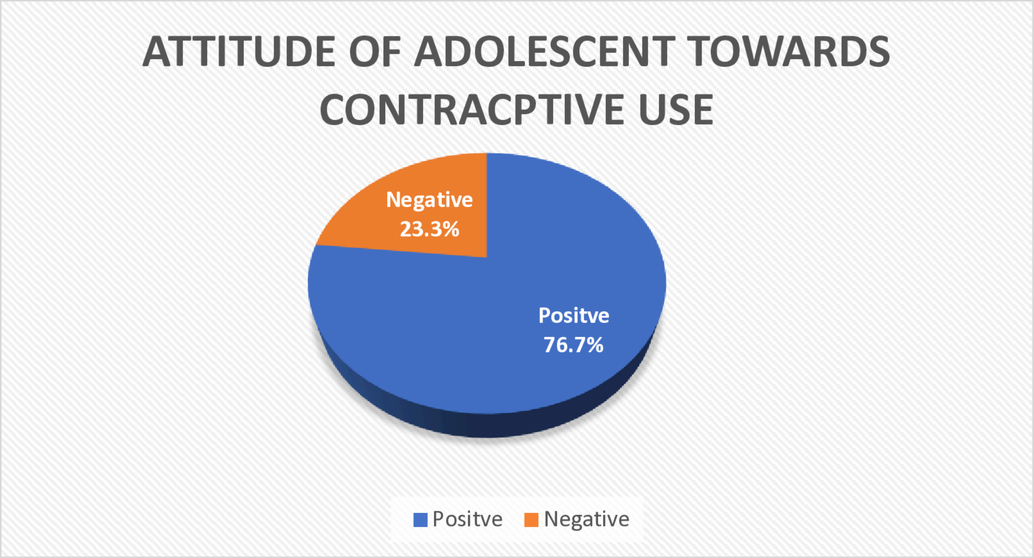

test-test-1 This is a preview of your figure rendered on a simulated PLOS journal page.

Maecenas ac est sit amet odio sollicitudin euismod. In risus odio, convallis a neque ac, varius ultricies arcu. Vestibulum et quam iaculis, ultricies odio et, molestie magna. Suspendisse vehicula purus id turpis eleifend, et convallis dui dignissim. Praesent tempus elit a metus sollicitudin, sed fringilla nulla porttitor. Nullam in tempus massa. Nunc maximus magna massa, nec volutpat risus rhoncus ut. Fusce quis ante sem. Aenean nulla nibh, tempus sit amet rhoncus at, eleifend vel risus. Sed dictum, sem ultrices elementum pharetra, lacus diam volutpat orci, scelerisque semper dui lacus ut enim.

Suspendisse in nunc id lacus commodo consequat. Proin semper aliquam varius. Fusce vitae neque aliquam nisi ultrices sodales vitae ut enim. Vivamus nec dictum ipsum. Sed condimentum ante eu urna tincidunt tincidunt. In ac lacus nec ipsum viverra volutpat posuere vel lacus. Class aptent taciti sociosqu ad litora torquent per conubia nostra, per inceptos himenaeos. Morbi rhoncus ipsum quis lorem hendrerit, at vulputate massa tempus. Ut arcu nisl, gravida vitae risus ultricies, porta venenatis massa. Cras dignissim, enim at faucibus aliquam, sapien nisl eleifend dolor, vel mollis nulla nisi id ipsum. Pellentesque vehicula ultricies risus sit amet faucibus. Praesent sit amet mi ac est faucibus accumsan. Praesent pulvinar sit amet orci auctor feugiat.

Click here to access/download
Supporting Information
S-1.docx

Click here to access/download
Supporting Information
S-2.docx

Click here to access/download
Supporting Information
S-3.xlsx

Click here to access/download
Supporting Information
S-4.docx

Click here to access/download
Supporting Information
S-5.docx

Full title: Sexual activity and contraceptive use among adolescents: A descriptive survey in a Ghanaian municipality

Short title: Contraceptive use among Senior High Sschool adolescents

Peter Boakye[1*], Evans Adaboh[1], Alberta Yemotsoo Lomotey[1], Jacob Tetteh[1], Abigail Kusi-Amponsah Diji[1], Victoria Bubunyo Bam[1]

[1] School of Nursing and Midwifery, College of Health Sciences, Kwame Nkrumah University of Science and Technology, Kumasi, Ghana

* peterboakye180@gmail.com

ABSTRACT

Contraceptive use is an imperative approach to reducing unwanted pregnancies and sexually transmitted infections (STIs) among adolescents. Sexual activity during adolescence, particularly in developing countries, remains a major public health concern with significant implications for reproductive health. Adolescents aged 10-19 experience heightened sexual drive due to hormonal changes, leading to early sexual activity and low contraceptive uptake, despite widespread knowledge of modern methods. The study aims to assess sexual activity and contraceptive use among Senior High School (SHS) adolescents, and to identify key predictors of contraceptive use. The study addresses gaps in literature regarding factors influencing contraceptive use among SHS adolescents, a group often overlooked in previous studies. A cross-sectional study was conducted from August to September 2022, ~~collecting data from~~ involving 330 ~~Senior High School (~~SHS~~)~~ adolescents selected through~~using~~ simple random sampling. Categorical data were analyzed using frequencies and percentages. Pearson's chi-squared test and binary logistic regression, were employed to assess associations and predict the relationship between independent variables, including sociodemographic characteristics (age, sex, class, ethnicity, religion, residential status), sexual activity characteristics (relationship status, multiple sexual partners), awareness of contraceptives and contraceptive use. Statistical significance was set at p ≤ 0.05 with a confidence interval of 95%. Out of the total participants, 290 (87.9%) were within the age range of 15-19 years and majority of the respondents were females (n=196, 59.4%). Approximately half (n=166, 50.3%) were in an intimate relationship ~~(n=166, 50.3%)~~ and 126(38%) had ever engaged in sexual intercourse. Of those who have ever engaged in sexual intercourse, over half (n=65, 51.6%) had their first sexual intercourse between the ages of

10-14 years and 100(79.4%) reported having ~~indicated that they have~~ ever used contraceptive. Almost two-thirds of those who had ever used contraceptive reported of having used condom, (n=66, 66.0%), followed by pills, (n=43, 43.0%), with~~and the least used method was~~ IUD being the least used method (n=2, 2.0%). Adolescents who had boyfriend or girlfriend ~~(AOR=5.604; 95%CL: 2.918-10.763)~~(AOR=7.208; 95%CL: 3.533-14.706), had~~having~~ multiple sexual partners ~~(AOR=10.631; 95%CL: 5.016-22.532)~~ (AOR=9.518; 95%CL: 4.320-20.969), and were~~being~~ aware of contraceptive (AOR=4.655; 95%CI: 1.395-15.530) ~~(AOR=5.105; 95%CI: 1.533-16.999)~~ had higher odds of contraceptive use than their counterparts. Early sexual initiation and contraceptive use are prevalent among adolescents. Comprehensive sex education and peer counseling on reproductive health are needed to address the consequences of early sexual activity without contraceptive use.

Keywords

Adolescents; sexual activity; contraceptive use; High School; Reproductive Health, Ghana

1.0 Introduction

Adolescent sexual activity is a global public health concern that significantly impacts reproductive health outcomes(1), especially in developing countries such as Ghana(2). Globally, more than 16 million adolescent girls aged 15–19 years give birth each year, most of which occur in Low-and Middle-Income Countries (LMICs)(3)(4). Adolescence is a

transitional phase of growth and development between childhood and adulthood between the ages of 10 to 19(5). This phase is a very critical period for sexual drive as a result of hormonal changes which occur naturally at this stage of life (6). In Ghana, as in many other countries globally~~other African nations~~, adolescents engage in sexual activity at earlier ages, often initiating sexual intercourse before the age of 15(7,8), contributing to a host of challenges, including unintended pregnancies, unsafe abortions and sexually transmitted infections (STIs)(7,9).

According to the World Health Organization(WHO) (5), 37.1% of female adolescents and 21.3% of male adolescents had ever had sex in Ghana. Among these, 9.9% of females and 6.9% of males reported having sex before the age of 15. ~~The number of births per 1,000 adolescent girls was 62.5 in 2020. Additionally, almost 20,000 adolescents were living with HIV and more than 2000 new cases every year~~ In 2020, the adolescent birth rate was 62.5 per 1,000 adolescent girls, and nearly 20,000 adolescents were living with HIV, with more than 2,000 new cases reported annually (5).

The World Health Organization emphasizes that adolescents have the right to access appropriate information and services regarding sexual and reproductive health(10). However, lack of comprehensive knowledge, misconceptions, fears, and social and cultural norms including religious prejudices limit adolescents' contraceptive use(11). Additionally, psychological factors, such as the fear of judgement from peers and parents, fear of side effects, and ~~along with~~ accessibility issues like the location of service points, make adolescents hesitant to discuss or seek contraceptive options(12–14). Consequently, many adolescents engage in unprotected sex, leading to a high prevalence of unintended

pregnancies and unsafe abortions, as well as increased~~ing~~ the risk of STIs(15,16). Studies indicate that ~~Ghanaian adolescents' ~~contraceptive use among Ghanaian adolescents remains low, despite the increasing knowledge about modern contraceptive methods(17–19). Multiple studies conducted among sexually active adolescents and young women reported high unmet need~~s~~ for modern ~~methods of ~~contraceptive~~s~~ methods(20,21)

One of the necessary approaches to reducing~~e~~ the burden of the consequences of unprotected sex~~ mentioned above~~ is the use of contraceptives (22). Contraceptive us~~ag~~e is very substantive in preventing unwanted pregnancies, unsafe abortions, and abortion-related complications, which~~that~~ expose adolescents to health related risks such as infertility and in some~~times~~ cases death (22). Globally, contraceptive use in regions such as Europe and North America exceeds 70%, whereas usage in Western and Central Africa remains below 25% , illustrating a wide gap in access and utilization ~~Compared to other regions like Europe, Latin America and the Caribbean, and Northern America contraceptive use in Middle and Western Africa is below 25 percent~~(23).

Many studies on adolescent sexual behaviour in the Ghanaian context have predominantly focused on adolescent girls and young women (18,24–26). Additionally, studies that included all adolescents have primarily focused on those in late adolescence (22,27,28), failing to capture the full range of adolescent experiences. Notably, research specifically targeting adolescents in senior high schools is lacking. These students are typically at a developmental stage where sexual experimentation increases (29), yet, their access to credible sexual and reproductive health information is often limited, and in some cases, they are unable to utilize the available information appropriately(29).

Contraceptive use in Ghana remains low, as reported by multiple studies(30–32). A study by Mavis et al(30) revealed that 26.7% of late adolescents used contraceptives in a cross-sectional study. Similarly, Begetayinoral et al(31) also reported that 26.3% of women of reproductive age were using modern contraceptives. These findings are below the national acceptor rate, which is 33.8%(33). In addition, ~~and the~~ factors influencing contraceptive use among high school adolescents have not been adequately assessed. ~~Therefore, this study aims to fill this gap by examining the sexual activity and contraceptive use among adolescents in senior high schools.~~ Addressing this issue is crucial not only for improving adolescent health outcomes but also for advancing economic growth and long-term national development. Understanding the factors that influence contraceptive use among adolescents is therefore essential, as existing literature provides important insights into the dynamics surrounding adolescent sexual and reproductive health(34).

Some studies have identified a wide range of factors associated with contraceptive use among adolescents(24,34,35). For instance, Michael et al(35), in a cross-sectional study among adolescent girls in some sub-Saharan countries, found that girls who had multiple children and prior knowledge of contraceptives were more likely to use them compared to those without such experiences. Similarly, a study by William et al(34) in Tanzania revealed that factors independently associated with contraceptive use included age, marital status, knowledge of contraceptives, cohabitation, and having multiple sexual partners. However, it is important to note that in the latter study, some participants were interviewed in the presence of their parents, which may have influenced their willingness to respond openly and honestly.

Therefore, given the sparse of literature on sexual activity and contraceptive use among adolescents in senior high schools, this study aims to assess the factors influencing contraceptive use within this population. Exploring the sexual behaviour and contraceptive use among this group is vital for informing interventions and policy reforms that will improve sexual and reproductive health.

2.0 Operational definitions

Independent variables were the sociodemographic variables (age, sex, class, religion, ethnicity, residential status [the person adolescent lives with], employment status of the person adolescent lives with) and sexual activity characteristics (relationship status, multiple sexual partners), and awareness of contraceptives. These were selected based on multiple existing literatures(18,24,34–36)

Adolescent: Individuals aged between 10-19 years, as defined by the World Health Organization (WHO), who are transitioning from childhood to adulthood and experiencing physical, hormonal, and emotional changes.

Sexual Activity refers to the engagement in any form of sexual intercourse (penile-vaginal), by adolescents. In this study, it includes individuals who reported having had sexual intercourse at least once.

Contraceptive Use: The use of any method (such as condoms, pills, IUDs, injectables, etc.) to prevent pregnancy and reduce the risk of sexually transmitted infections (STIs) during sexual intercourse. In this study, contraceptive use refers to adolescents who reported using any form of contraception.

3.0 Materials and methods

3.1 Study design

A descriptive cross-sectional study was deemed appropriate as the researchers aimed to collect data at a specific point in time and describe the characteristics of adolescents concerning sexual activity and contraceptive use(37).

3.2 Study setting

The study was conducted within a senior high school in Ejisu municipality in the Ashanti region of Ghana,~~ Ghana,~~ which constitutes both rural and urban localities(38). In the Ghanaian context, s~~S~~enior high school is a second-cycle institution that students attend after one graduate from Junior high school, typically covering grades equivalent to 10 through 12 in the international education system~~.~~ This school was selected as the study setting due to its diverse student population and its ability to cater for students from various economic backgrounds. The school is a public mixed school which admits day students and ~~it~~ also has boarding facilities for a section of the student body. The school offers five (5) programmes which include; Business, Home Economics, Visual Arts, General Arts and General Science. ~~The average yearly enrollment is about 600.~~ Facilities within the school include classrooms, administrative offices, a library, laboratories and outdoor spaces such as playgrounds and sports fields. This school was purposively selected for its unique representation of both urban and rural student populations. The school's status as a large, public, mixed-gender institution offering a wide range of academic programs made it an ideal microcosm for exploring adolescent sexual and reproductive behaviors in the municipality. Additionally,

logistical feasibility and the willingness of the school management to approve the study were also factors in its selection.

3.3 Study population

Between 16/08/2022 and 15/09/2022, adolescents of both genders in senior high school were selected for the survey.~~a total of 330 adolescents participated in the survey~~. The actual age range of participants in this study was 11 to 19 years. However, for the purposes of analysis and discussion, participants were categorized into early adolescence (10–14 years) and late adolescence (15–19 years), based on the WHO classification(39). SHS1 students were excluded from the study as they were on vacation during the data collection period. As a result, only SHS 2 and SHS 3 students were included in the study.

~~The study population was made up of adolescent from Senior High School. Adolescents were categorized into two age groups; early (10–14 years) and late (15–19 years).~~

3.4 Inclusion and exclusion criteria

Adolescents aged 10 to 19 who were present in school at the time of the study and could speak, understand, and write English were eligible to participate. Sections of students who were on vacation, suspension, critically ill, or had mental impairments were excluded from the study.

3.5 Sample size determination and sampling

A sample size of 330 was determined using the Slovin's formula:

$n = \frac{N}{1+N(e)^2}$ (40), and with a confidence interval of 95% (at a Z-score of 1.96), a margin of error of 5%, where **n** signifies the sample size, **N** signifies the population under study and **e** signifies the margin of error (0.05). The total population was 1210 using the total enrollment of students at the time of the study obtained from the school's administrative office. The minimum required sample size obtained was 300. A 10% non-response rate was considered to compense for a non- response, resulting in a total estimated sample size of 330.

3.6 Data collection Tool

A structured questionnaire was used in this study, developed based on current literature and tailored to the study context. The questionnaire was pretested to ensure clarity and relevance. It was divided into six sections, the first section was on s~~S~~ociodemographic characteristics of adolescents (7 items).~~:~~ This section included~~s~~ questions on age, gender, class, religion, ethnicity, residential status (i.e., whom the adolescent lives with), and the employment status of the person the adolescent stays with. Second section focused on~~was~~ sexual activity (8 items).~~:~~ This section covers topics such as whether the respondent has a boyfriend or girlfriend, the age of their sexual partner, whether they have ever had sex, the number of sexual partners, the age of first sexual intercourse, how the first sexual experience occurred (whether they were coaxed, forced, or it was their own will), and whether they feel pressured to have sex. Section three was awareness of contraceptives (11 items).~~:~~ Questions in this section included whether the respondent has heard of modern contraceptives, their source of information, the methods of contraception they were aware of, where one can access contraceptives, as well as similar inquiries regarding emergency contraceptives. The fourth section examined~~was~~ contraceptive use (11 items).~~:~~ This section included~~s~~ questions on whether the respondent had~~s~~ ever used contraceptives, which methods they had~~ve~~ used,

whether they used contraceptives every time they had~~ve~~ sex, and the reasons for using them, such as “to avoid teenage pregnancy” or “to prevent STIs.” Other questions included whether they used contraceptives during their first sexual intercourse, where they accessed contraceptives, and how frequently they used them, with options like “every time I have sex,” “only during my first sexual intercourse,” or “once in a while.” The fifth section was on attitudes of adolescents towards contraceptives (7 Likert scale items).~~:~~ This section included~~s~~ statements like “I approve of adolescents using contraceptives every time they have sex.” Higher scores reflect a positive attitude. Other statements, presented in a negative form and were reverse-coded, included: “Using contraceptives before a girl’s first birth can lead to infertility,” “Sex is not enjoyable when I use a condom,” and “People who insist on condom use are promiscuous.” Sixth section was barriers to contraceptive use among adolescents (8 items).~~:~~ Respondents were asked to indicate whether certain factors were barriers to their contraceptive use. These factors included religious beliefs, the attitude of providers, fear of side effects, embarrassment when buying contraceptives, fear of being seen by parents, partner disapproval, unavailability of contraceptives, and cultural beliefs.

3.7 Data collection procedure

A structured questionnaire was administered to 330 adolescents attending a Senior High School. A simple random sampling technique was employed to ensure an unbiased selection of participants(41). The researchers first obtained permission from the school administration, and students were briefed on the purpose of the study before participation. Participants were asked to choose between ~~two~~ sealed opaque folded papers with an equal

number ~~one~~ labelled 'yes' and ~~the other~~ 'no'. Those who selected 'yes' after providing informed consent were included in the study.

To minimize potential bias, teachers were asked to leave the classrooms during the questionnaire administration to ensure participants did not feel intimidated or coerced. The questionnaire, comprising 45 questions, was distributed to participants, and they were given a maximum of one hour to complete it. Data was collected in all classes simultaneously to prevent students from discussing the questionnaire's content beforehand. After collection, the completed questionnaires were reviewed by the research team to ensure completeness and accuracy before analysis.

3.8 Measurement of variables

Sexual activity, awareness of contraceptives and contraceptive usage

Sexual activity, awareness of contraceptives, and contraceptive use were measured using structured questions with binary response options (Yes/No). The percentage of participants who responded "Yes" was used to describe prevalence of each variable.

Attitudes toward contraception

Attitudes toward contraceptive use were assessed using a seven-item tool graded on a five-point Likert-scale (1 = Strongly Disagree, 2 = Disagree, 3 = Neutral, 4 = Agree, and 5 = Strongly Agree). Higher scores indicated more positive attitudes toward contraceptive use. Three of the items were phrased negatively ("Using contraceptives before a girl's first birth can lead to infertility," "Sex is not enjoyable when I use a condom," and "People who insist on condom use are promiscuous") and were reverse-coded before analysis so that higher scores

consistently reflected a positive attitude. The total attitude score was then calculated by summing the responses to all seven items, with a possible range of 7 to 35. Respondents with total scores above the median were classified as having a positive attitude, while those scoring below the median were considered to have a negative attitude toward contraceptive use.

3.9 Data processing and analysis

The researchers confirmed the completeness of all collected questionnaires by reviewing each section, ensuring there were no missing data. Serial numbers were assigned to the questionnaire and were entered into Microsoft Excel for data cleaning and transferred to Statistical Product and Service Solutions (SPSS) version 22.0 for Windows (IBM SPSS Statistics) for the data to be analysed. Descriptive statistics (frequencies and percentages) were used to describe the categorical characteristics of participants.

To identify the factors associated with contraceptive use, first chi-square test was done to assess the association between the independent variables and the dependent variable. The ones that were found to be significant were further analyzed using binary logistic regression.

In model 1, univariate binary logistic regression was performed for each significant variable from the chi-square tests to calculate crude odds ratios (OR).

Model 2 was a multivariable binary logistic regression that included all independent variables (relationship status, multiple sexual partners, and awareness of contraceptives) found to be significant in Model 1, while adjusting for control variables such as age, sex, class, religion, and residential status. Adjusted odds ratios (AOR) and 95% confidence intervals (CI) were reported. Statistical significance was set at $p \leq 0.05$.

Multivariable logistic regression analyses were performed and reported using odds ratio and95% confidence intervals. The significance level was set at a p-value of ≤ 0.05.

Operational definitions

Sociodemographic variables (age, sex, class, religion, ethnicity, residential status [the person adolescent lives with], employment status of the person adolescent lives with).

Adolescent: Individuals aged between **10-19 years**, as defined by the World Health Organization (WHO), who are transitioning from childhood to adulthood and experiencing physical, hormonal, and emotional changes.

Sexual Activity refers to the engagement in any form of sexual intercourse (penile-vaginal), by adolescents. In this study, it includes individuals who reported having had sexual intercourse at least once.

Contraceptive Use: The use of any method (such as condoms, pills, IUDs, injectables, etc.) to prevent pregnancy and reduce the risk of sexually transmitted infections (STIs) during sexual intercourse. In this study, contraceptive use refers to adolescents who reported using any form of contraception.

3.10 Quality assurance; Validity

To ensure content validity, we invited fivesix public health experts to assess the relevance of each question to its corresponding factor through a face-to-face approach. This process helped determine whether the questionnaire accurately measured the intended information. Minor adjustments were made to some questions by all five experts to enhance their cultural relevance and acceptability to the study participants.

3.11 Ethical considerations

Prior to the conduct of the study, an institutional-based approval was sought from the management of the school in order to conduct the research in the school. Ethical approval, referenced as CHRPE/AP/426/22, was granted by the Committee on Human Research, Publication, and Ethics (CHRPE) at the School of Medicine and Dentistry, Kwame Nkrumah University of Science and Technology in Kumasi, Ghana. Since majority of the participants resided in the school's boarding house and many parents lived far from the school, direct consent from parents was not feasible. Therefore, verbal consent was obtained from the school authorities acting in loco parentis due to the boarding school arrangement for the participation of the students. The participants were briefed on the research's objectives before seeking their consent to participate. They were provided with an opportunity to inquire about the study procedures. Verbal consent was documented by the research team and witnessed by the teachers who acted as custodians in the school setting. Participation was entirely voluntary, with no direct benefits to participants, and they had the freedom to withdraw at any point. To protect anonymity, no names or other identifying details were obtained.

4.0 RESULTS

4.1 Sociodemographic characteristics of adolescents

Majority of the participants were in their late adolescents' stage (n=290, 87.9%), and were females (n=196, 59.4%). Most of the participants lived with both parents (n=194, 58.8%), and were Christians (90.9%, n=300) ***(Table 1).***

Table 1. Sociodemographic characteristics of adolescents

Variable	Frequency	Percentage (%)
**Age**		
10-14	40	12.1
15-19	290	87.9
**Sex**		
Male	134	40.6
Female	196	59.4
**Class**		
SHS Level 2	190	57.6
SHS Level 3	140	42.4
**Religion**		
Christian	300	90.9
Muslim	30	9.1
**Ethnicity**		
Akan	272	82.4
Ga-Adangbe	12	3.6
Ewe	18	5.5
Others (Dagoba, Mamprusi, Fafra)	28	8.5
**Residential status**		

Living with both Parent	194	58.8
Living with Single parent	120	36.4
Living alone	16	4.8
**Employment status [of the person adolescent lives with]**		
Unemployed	129	39.1
Employed	201	60.9

4.2 Sexual activity among adolescents

Half of the participants (n=166, 50.3%) had boyfriends or girlfriends and majority had multiple sexual partners (n=64, 50.2%). Additionally, over a-third of participants (n=126, 38.2%) have ever had sex, and of this, more than half (n=65, 51.6%) experienced their first sexual intercourse during early adolescence, between the ages of 10 and 14 years. Participants who engaged in sex on their own will were (n= 87, 69%). Furthermore, 119 (36.1%) of the participants reported feeling pressured to have sex, predominantly from friends (n=70, 58.8%) ***(Table 2).***

Table 2. Sexual Activity among Adolescents

**Variable**	**Frequency**	**Percentage (%)**
**Having a Boyfriend/Girlfriend**		
Yes	166	50.3
No	164	49.7
**Age of boyfriend/girlfriend (N=166)**		

10-19	108	65.0
More than 19	58	35.0
**Ever had sex**		
Yes	126	38.2
No	204	61.8
**Do you have multiple partners**		
Yes	65	19.7
No	265	80.3
**Number of sexual partners (N=126)**		
1	62	49.2
2	24	19.0
3	12	9.6
More than 3	28	22.2
**Age of first Sexual Intercourse**		
10-14	65	51.6
15-19	61	48.4
**How first sexual intercourse happened (N=126)**		
Own will	84	66.7
Coaxed	17	13.5
Forced	15	11.9
**Pressure to have sex**		
Yes	119	36.1
No	211	63.9

**Sources of pressure to have sex (N=119)**		
Friends	70	58.8
Relatives	12	10.0
Teachers	10	8.4
Boyfriend/Girlfriend	27	22.7

4.3 Awareness of contraceptives among Adolescents

Majority of the participants (n=283, 85.8%) surveyed were aware of contraceptive methods. Study results indicated that (n=155, 54.8%) were aware of male condoms; the least (n=12, 4.20%) were aware of IUD. Among those who knew about the contraceptive methods, television (n=144, 50.9%) and peers (n=113, 39.9%) were the major sources of their information and the least were parents (n=10, 5.4%). On whether modern contraceptives provide 100% protection from pregnancy, a notable number of participants, 117(35.5%) reported that they did not know ***(Table 3).***

Table 3. Awareness of contraceptives among Adolescents

Variables	Frequency	Percentage (%)
**Heard of contraceptives**		
Yes	283	85.8
No	47	14.2
**Heard of any method of contraceptive**		
Yes	283	85.8
No	47	14.2

**Where did you hear it from (N=283)**		
Radio	105	37.1
Television	144	50.9
Peers	113	39.9
Parent	62	21.9
Teacher	81	28.6
Health worker	79	27.9
Internet	113	39.9
**What method of contraception do you know (N=283)**		
Male condom	155	54.8
Abstinence	35	12.4
Implant	18	6.4
Withdrawal method	44	15.5
Female condom	40	14.1
IUD	12	4.2
Urinating after sex	30	10.6
Injectable	20	7.1
Pills	96	33.9
**Heard about emergency contraceptive**		
Yes	185	56.1
No	145	43.9
**If yes, where did you hear about this emergency contraceptive from (N=185)**		
Radio	88	47.6

Television	103	55.7
Peers	74	40
Health worker	73	39.5
Teacher	26	14.1
Internet	73	39.5
Parents	10	5.4
**How often do you know emergency contraceptives can be used within a year (N=185)**		
Once a year	40	21.6
Twice a year	48	26.5
Three times a year	22	11.4
More than three times a year	75	40.5
**Do you know where you can access contraceptive**		
Yes	249	75.5
No	40	12.1
Unanswered	41	12.42
**If yes, where (N=249)**		
Pharmacy	194	58.8
Friend	51	15.5
Hospital or clinic	208	63.0
Health personnel	68	20.6
**Can a girl become pregnant from just one sexual intercourse**		
Yes	240	72.7
No	29	8.8

I don't know	61	18.5
**Do contraceptives provide 100% protection from pregnancy**		
Yes	79	23.9
No	134	40.6
I don't know	117	35.5

4.4 Use of contraceptives among adolescents

Among the participants who have ever had sex, 100 (79.4%) had ever used contraceptives. Condoms (n=66, 66%) and pills (n=43, 43%) were the most common methods of contraception. Contraceptive use during the first sexual encounter was reported by 67 (67%). The majority of the participants used contraceptives to avoid pregnancy (n=58, 58.0%). Regarding the frequency of emergency contraceptive usage, 20 (42.3%) indicated they use it every time they have sex ***(Table 4)*.**

Table 4. Use of contraceptive among adolescents who have had sex

Variable	Frequency	Percentage (%)
**Use of Contraceptives(N=126)**		
Yes	100	79.4
No	26	20.6
**Method of Contraceptive used (N=100)**		
Condom	66	66.0
Pills	43	43.0

IUD	2	2.0
Implants	3	3.0
Injectable	3	3.0
Withdrawal	14	14.0
Safe Period	23	23.0
**Do you use contraceptive anytime you have sex**		
Yes	50	50.0
No	50	50.0
**Reasons for using contraceptives (N=100)**		
To avoid teenage pregnancy	58	58.0
To prevent STI	28	42.0
**Discuss Contraceptive use with your partner(N=100)**		
Yes	59	59.0
No	31	31.0
I do not remember	10	10.00
**Use of contraceptive first time you have sex**		
Yes	67	67.0
No	33	33.0
**Method of Contraceptive use first time of having sex**		
**(N=67)**		
Condom	46	68.7
Pills	32	47.8
Implants	3	4.5

IUD	1	1.5
Withdrawal	12	17.9
Safe Period	18	26.7
Injectable	1	1.5
**Access to Contraceptive(s) (N=67)**		
Hospital	16	23.9
Pharmacy	49	73.1
Friends	22	32.8
Health Personnel	7	10.4
**Frequency of Contraceptive use (N=100)**		
Every time I have sex	50	50.0
Only on my first sexual intercourse	7	7.0
Once a while	43	43.0
**Use of Emergency Contraceptives(N=75)**		
Yes	47	62.7
No	28	37.3
**Frequency of Emergency Contraceptive use (N=47)**		
Every time I have sex	20	42.5
Once a year	10	21.3
Twice a year	9	19.1
Three times a year	4	8.5
More than three times a year	4	8.5

4.5 Attitude Towards Contraceptive Use

Participants were asked to indicate their level of agreement to seven statements on contraceptive use, using a five-point Likert scale rating of strongly agree=5, agree=4, uncertain=3, disagree=2 and strongly disagree=1. All the negative worded statements were reverse-coded. The minimum, maximum and median scores were 7, 35 and 18 respectively; participants whose overall scores were above the median score (18) were said to have a positive overall attitude towards contraceptive use. Overall, 76.7% of the adolescents were found to have a positive attitude towards contraceptive use ***(Figure 1).***

Responses Regarding Attitude of Adolescents Towards Contraceptive Use

From the survey, majority (n= 80, 24.2%) agree with the usage of contraceptives by adolescents. Most of the participants (n=129, 39.1%) were uncertain that the use of contraceptives by a girl before her first birth can lead to infertility. Approximately, 117(35%) of the respondents were not certain as to whether "sex is not enjoyable when I use condom". Participants whose religion was against usage of contraceptives were (n=141, 42.7%), and 207(62.7%) reported contraceptive use is not only the responsibility of females ***(Table 5)***.

Table 5. Attitude of adolescents towards contraceptive use

**Variable**	**Frequency**				
	**Strongly disagree**	**Disagree**	**Uncertain**	**Agree**	**Strongly agree**

I approve the use of contraceptive by adolescents anytime they have sex [preferred]	53(16.1%)	73(22.1%)	58(17.6%)	80(24.2%)	66(20.0%)
Use of contraceptive by a girl before her first birth can lead to infertility	35(10.6%)	46(13.9%)	129(39.1%)	73(22.1%)	47(14.2%)
Sex is not enjoyable when I use condom	51(15.5%)	30(9.1%)	117(35.3%)	71(21.5%)	61(18.5%)
People who insist on condom use are promiscuous	44(13.3%)	68(20.6%)	86(26.1%)	68(20.6%)	64(19.4%)
Contraception usage is against my religion	59(17.9)	75(22.7%)	55(16.7%)	74(22.4%)	67(20.3%)
I will use contraception in the future	96(29.1%)	79(23.9%)	79(23.9%)	47(14.2%)	29(8.8%)

Contraception is the responsibility of only females	125(37.9%)	82(24.8%)	51(15.5%)	42(12.7)	30(9.1%)

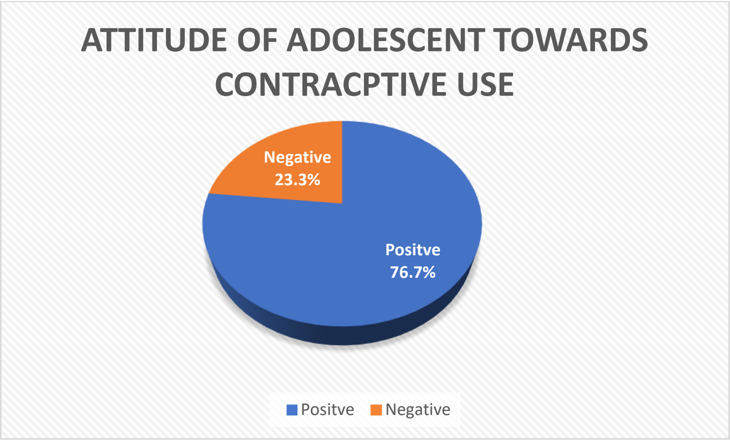

Fig 1. Attitude of Adolescent towards contraceptive use.

4.6 Barriers to contraceptive use among adolescents

Among these barriers to the use of contraceptives, majority of the respondent reported fear of side effects of contraceptive as a barrier (n=16, 63.2%). Other barriers were religious beliefs (n=14, 55.3%) and embarrassment on the part of adolescents (n=9, 35.5%). The least reported barrier to the use of contraceptive was unavailability of contraceptive (n= 2, 9.2%) ***(Table 6).***

Table 6. Barriers to contraceptive use among adolescents

Variable	Frequency	Percentage

**Reason(s) for not using any of the contraceptive methods (N=26)**		
Religious beliefs	14	55.3
Attitude of contraceptive providers	3	14.5
Fear of side effects	16	63.2
Embarrassment to buy	9	35.5
Fear of being seen by parents	8	30.3
Partner disapproval	4	17.1
Unavailability of contraceptive	2	9.2
Cultural beliefs	7	28.9

4.7 Association between socio-demographic characteristics of Adolescents and use of contraceptives

The study found that having a boyfriend or girlfriend, the number of sexual partners an adolescent had, and awareness of modern contraceptives (all with a p-value of $p \leq 0.001$) were statistically significant factors influencing contraceptive use. There was no significant association between some of the sociodemographic characteristics (age, sex, class, religion, ethnicity, residential status, and employment status of the person adolescent lives with) ***(Table 7).***

Table 7. Association between socio-demographic characteristics of Adolescents and use of contraceptives

Variables	Usage of contraceptive		Chi-square	Total
	Yes, (%)	No, (%)	(p-value)	(N)
Age				330
14-16	20.0	80.0	0.309	40
17-19	31.7	68.3		290
Sex				330
Male	30.6	69.4	0.700	134
Female	30.1	69.9		196
Class				330
SHS 2	29.8	70.2	0.666	191
SHS 3	30.9	69.1		139
Religion				330
Christian	29.3	70.7	0.220	300
Muslim	40.0	60.0		30
Ethnicity				330
Akan	31.7	68.3	0.887	243
Fante	20.7	79.3		29
Ga-Adangbe	41.7	58.3		12
Ewe	33.3	66.7		18
Others (Dagoba, mamprusi, Fafra)	25.9	74.1		28

Residential status				330
Both parent	26.3	73.7	0.072	194
Single parent	34.1	65.9		120
Live alone	50.0	50.0		16
Employment status				330
Unemployed	31.8	60.2	0.920	129
Employed	29.4	70.6		201
Do you have a boyfriend/girlfriend				330
Yes	52.4	47.6	≤0.001*	166
No	7.9	92.1		164
Number of sexual partners				330
0	0.0	100.0	≤0.001*	204
1	71.0	29.0		62
2	75.0	25.0		24
3	66.7	33.3		12
More than 3	89.2	10.8		28
Heard of modern contraceptive				330
Yes	65.7	34.3	≤0.001*	283
No	6.3	93.7		47

4.8 Logistic regression predicting modern contraceptive use

~~The model explained between 43.3% (Cox & Snell R^2) and 61.4% (Nagelkerke R^2) of the variance in modern contraceptive use. The Hosmer-Lemeshow test produced a p-value of 0.512, indicating that the model fits the data adequately. Overall, the model correctly classified 90.0% of cases.~~ Age, residential status, religion, class, and gender were included as control variables, however, none of the control variables significantly predicted contraceptive use. The independent variables, whether the respondent had~~s~~ a boyfriend or girlfriend, the number of sexual partners, and whether the respondent had~~s~~ heard of modern contraceptives were statistically significant predictors in the model. Adolescents who had heard of modern contraceptives were 4.655 times more likely to use them compared to those who had not (AOR = 4.655, 95% CI = 1.395–15.530, $p = 0.012$). Similarly, those with multiple sexual partners were significantly more likely to use contraceptives than those without (AOR = 9.518, 95% CI = 4.320–20.969, $p < 0.001$). Additionally, having a boyfriend or girlfriend was associated with approximately seven times higher odds of contraceptive use (AOR = 7.208, 95% CI = 3.533–14.706, $p < 0.001$). These associations remained significant after adjusting for the control variables in the model ***(Table 8).***

Table 8. Logistic regression predicting modern contraceptive use

Variable	B(Coefficient)	Adjusted Odds Ratio (EXP(B))	95%CL	p-value
Age (years)				
10-14years	0.216	1.241	0.464-3.322	0.667

15-19years**(ref)**				
Sex				
Male	0.017	1.017	0.505-2.047	0.963
Female**(ref)**				
Religion				
Christian	-0.240	0.787	0.283-2.183	0.645
Muslim**(ref)**				
Residential status				
Both parents	-0.147	0.863	0.206-3.620	0.841
Single parent	0.091	1.095	0.255-4.697	0.902
Live alone**(ref)**				
Heard of modern contraceptive				
Yes	1.538	4.655	1.395-15.530	0.012*
No**(ref)**				
Do you have multiple partners				
Yes	2.253	9.518	4.320-20.969	< 0.001*
No**(ref)**				

Do you have boyfriend				
Yes	1.975	7.208	3.533-14.706	< 0.001*
No**(ref)**				
Class				
SHS 2	-0.380	0.684	0.344-1.359	0.278
SHS 3**(ref)**				

5.0 Discussion

This study aimed to assess ~~the~~ sexual activity and contraceptive use among adolescents. Findings from the study showed that~~,~~ a little over half of adolescents were~~are~~ in a relationship with someone of the opposite sex. This corroborates findings from~~of~~ other studies, which have ~~that~~ shown~~ed~~ that~~,~~ the majority of adolescents have~~d~~ ~~ever~~ been in a relationship with someone of the opposite sex (36,42).

Furthermore, the study found that~~,~~ among adolescents who ~~have~~ had engaged sexual activity, the majority ~~of them~~ had their sexual debut during~~at an~~ early adolescence stage (10-14 years). This may~~could~~ be explained by the exploration and heightened sexual curiosity that occur among adolescents during their critical growth phase of identity formation. A study conducted in Kenya reported that adolescents who initiate sex at an early stage are influenced by peer pressure, as well as easy access to phones and the internet~~ at an early age~~(43). This early exposure may be due to increased exposure to erotic materials (nudity, photographs/videos of sexual activity), which are readily available. This increases their

curiosity and finally commit to the act of sexual activity. This is further supported by the findings of this current study, which revealed that adolescents mostly feel pressured~~ized~~ by~~from~~ their friends and their boyfriend or girlfriend to have sex.

The study also reported high percentage of adolescents having multiple sexual partners. This is consistent with the findings of Attibu (42) who reported that nearly half of the adolescent in the study had~~were having~~ more than one sexual partner. This can be an indicator of increased~~for~~ risk ~~o~~for pregnancy, unsafe abortion, and STIs, implying that adolescents may~~do~~ not maintain long-term sexual relationships but instead~~ may~~ enter new relationships due to unmet expectations, such as financial rewards or other benefits (44).

The findings from this study revealed that~~,~~ awareness of contraceptive was high among adolescents. This is in keeping with a study conducted by Agbanyo(17)~~, Agbanyo, (2018)~~ which revealed that~~,~~ 88% of adolescents were aware of contraceptives. From our findings, 86.1%~~most~~ of~~ the~~ adolescents who had~~ve~~ heard about contraception~~, 86.1% ~~ were able to mention at least one type of contraceptive and where it could~~an~~ be accessed. This high awareness of contraception may~~could~~ be attributed to~~ the~~ recent change in social media advertisement which includes both video and audio content.

In this study, the most frequently mentioned sources of information on contraceptives were television, peers and radio which is similar to findings from research conducted in Tanzania (45). Additionally,~~lso a~~ study~~research~~ by Hagan and Buxton~~,~~ indicated that, majority (60.0%) of adolescents reported receiving ~~said they got their~~ information through the radio or television(46). The least source of information on contraceptives use was found to be from parents. This is in agreement with a study conducted by Hagan and Buxton, ~~-~~which also

revealed that, only 3.3% of adolescents received information on contraceptive from parents(46). This could be as a result of African cultural setting where discussing sexual issues at~~in the~~ home~~use~~ is often considered ~~seen as an~~ immoral behaviour (47). As a results,~~Sometimes, this makes it uncomfortable for~~ both adolescents and adults may feel uncomfortable discussion topics related ~~the elderly~~ to ~~share ideas concerning~~ sexuality and contraceptive use. The source of information is a major factor in providing adolescents with correct information regarding contraceptives and ~~i~~theirs~~s~~ use. A reliable source of information from health care providers and mass media provide adolescents with more accurate information than friends (45).

Findings~~rom the~~ from~~indings~~ ~~of~~ the current study revealed~~,~~ a high ~~the~~ prevalence of contraceptive use among adolescents, ~~was high~~ which exceeds the~~is currently above~~ Ghana Health Service's national family planning target of 33.8%(33), ~~23.3%~~ and is higher than what has been reported in other studies conducted among adolescents(34,35). This is in agreement with findings from a study by Maness et al, which also showed high usage of contraceptives by adolescents(48). However, t~~T~~his is inconsistent with other studies which revealed low use of contraceptives among adolescents(34). For instance, findings from a study by Kinaro et al (49) revealed that only 8.6% of all adolescents had ever used a contraceptive method. The high rate ~~use~~ of contraceptive use in the present study~~s~~ may probably be attributed to the high level of awareness as observed among participants~~revealed by findings of the current study~~.

Condom and Pills were the most commonly used contraceptives by adolescents. Condoms were the most frequently used method of contraception probably because it is the most

popular, easily accessible, affordable~~cheap~~, and have dual protection against teenage pregnancy and sexually transmitted diseases (50). The majority of adolescents accessed contraceptives from the pharmacy and peers,~~.~~ which~~This~~ is in line with the findings of Kara et al(45). Adolescents tend to prefer to obtaining~~access~~ condoms and pills from pharmacies,~~y shops~~ like~~probab~~ly because it is easily accessible in almost every community as compared to hospitals. This corroborates with a study conducted by Chandra-Mouli and Akwara, which revealed that pharmacies offer adolescents accessible locations, longer operating hours, and most~~re~~ importantly, anonymity in obtaining contraceptive~~ method~~s(51). The study further indicated that the preference for pharmacies was as a result of difficulty in obtaining contraceptives from health facilities due to restrictive laws, health worker bias, or privacy concerns

Adolescents exhibited positive attitudes towards contraceptive us~~ag~~e. The present study reported that~~,~~ majority of adolescents approved the "use contraceptive anytime they have sex". The~~i~~se findings ~~is in~~ contrast with those of~~to~~ a previous study by Sam, where the level of awareness and attitude towards ~~the use of~~ contraceptives use among adolescents were found to be very low and only few of them have ever used or currently using effective contraceptive methods(52).

However, a few participants exhibited negative attitudes towards contraceptive use. For instance, the most~~ajority~~ of adolescents in this study supported the idea that~~ the use of~~ contraceptive use by a girl before her first birth can lead to infertility. This is in agreement with a study conducted by Sam, where 67.0% of respondents strongly agreed that a girl using contraceptives before having her first child could~~an~~ cause infertility(52). This is also

consistent with Lauren et al, who found that future fertility was among the main attitudes which tend to be an obstacle to ~~the use of~~ contraceptives use(53). Adolescents have a misconception about contraceptive use,~~.~~ Possibly~~erhaps, this is~~ because much of the information they receive comes from~~on contraception given by~~ unreliable sources like friends who lacked in-depth knowledge~~ was high~~. Additionaly, concerns~~This~~ may also be as a result of the side effects of contraceptives (especially oral pills and emergency contraceptives) on the menstrual cycle of female adolescents.

With regard to contraceptive use, awareness, number of sexual partners, and having a boyfriend or girlfriend were found to influence adolescents' use of contraceptives. This finding contrasts with a study conducted in Nigeria by Abdul et al among university students, which found~~revealed~~ no association between awareness and contraceptive use(54).

Research conducted by James et al. showed a significant association between the number of sexual partners and contraceptive use(55). Adolescents with two or more sexual partners are more likely to engage in risky sexual behaviors (56). To prevent unintended pregnancies, STIs, school dropout, and unsafe abortions, they often resort to contraceptive use. This aligns with the findings of the current study, whi~~ch~~ch~~ere~~ adolescents with multiple partners were found to be nine~~four~~ times more likely to use contraceptives. Additionally, all adolescents who had ever used contraceptives indicated that their primary reasin was either to~~he~~ prevent~~ion of~~ unwanted pregnancy or~~ the~~ prevent~~ion of~~ STIs~~ as their primary reason for use~~.

Being in long-term or casual relationships is also significantly associated with effective contraceptive use (36). This finding is consistent with the results of the current study, which

showed that~~ere~~ having a boyfriend or girlfriend increased an adolescent's likelihood of using contraceptives by approximately seven~~three~~ times.

6.0 Conclusion and recommendation

Based on the findings of this study, it is evident that adolescent sexual activity and contraceptive use are significant issues that require attention and targeted interventions. The prevalence of multiple sexual partners and early sex debut among adolescents reflects potential risks such as unintended pregnancies, unsafe abortions, and sexually transmitted infections (STIs). On a positive note, there is a high level of awareness and positive attitudes towards contraceptive use among adolescents. However, misconceptions persist, potentially due to unreliable sources of information. Given this, healthcare providers and other relevant~~interested~~ stakeholders should use multidimensional and individualized interventions to improve the reproductive health of adolescents.

Parents and guardians should be encouraged to educate adolescents about~~on~~ sexual activity and contraceptive use. Collaboration between the Ministry of Health and the Ministry of Education is essential to organize regular health seminars to keep adolescents well-informed about~~on~~ issues related to sexuality and contraception. Additionally, there is a need to reconsider incorporating comprehensive sex education starting at the basic school level. Peer educators should be identified and trained to provide sexual education, as adolescents often~~tend to~~ feel more comfortable seeking information from their peers. Finally, future studies should explore the role of parents and guardians in influencing adolescents' sexual activity and contraceptive use.

Efforts should be directed towards providing training to health workers and educators on how to effectively communicate accurate, ~~and~~ evidence-based information to parents and the general public. This can include workshops on health communication and the use of trusted sources.

7.0 Limitations of the study

The use of self-reporting and the school environment may have influenced ~~the~~ participants to provide responses that they perceived as socially desirable. In addition, the researchers used a small sample size. However, the findings of the current research have relevant implications for improving adolescent sexual and reproductive health. Future research should prioritize assessing sexual activity and contraceptive use beyond the school setting.

8.0 Acknowledgements

The authors sincerely thank the management of the school for granting us the opportunity to conduct this study in their esteemed institution. We also extend our heartfelt gratitude to the students who participated in this study.

References

1. Jing Z, Li J, Wang Y, Zhou C. Prevalence and Trends of Sexual Behaviors Among Young Adolescents Aged 12 Years to 15 Years in Low and Middle-Income Countries: Population-Based Study. JMIR Public Heal Surveill. 2023;9.

2. Alhassan AR, Wepeba TW, Abdulai K, Iddrisu R, Aninanya GA. Prevalence and

Associated Factors of Adolescent (15-19 Years) Childbearing in Ghana. Biomed Res Int. 2024;2024.

3. WHO. Adolescent pregnancy fact sheet. Adolesc Pregnancy Fact Sheet [Internet]. 2020;1. Available from: https://www.who.int/news-room/fact-sheets/detail/adolescent-pregnancy

4. Ndahimana R, Umutoni NJ, Nteziryayo JP, Semafara S, Binayisa G, Ishimwe J, et al. Prevalence, Trends in and Determinants of Teenage Pregnancies in Rwanda: Analysis of Rwanda Demographic and Health Survey (2010 to 2020). J Biomed Res Environ Sci. 2023;4(10):1425–34.

5. WHO. Ghana Contraception within the context of adolescents' sexual and reproductive lives: Country profile Microsoft Word - UGANDA. 2021;5:9–11. Available from: who.int

6. Vijayakumar N, Op de Macks Z, Shirtcliff EA, Pfeifer JH. Puberty and the human brain: Insights into adolescent development. Neurosci Biobehav Rev [Internet]. 2018;92(January):417–36. Available from: https://doi.org/10.1016/j.neubiorev.2018.06.004

7. Kyei-Arthur F, Agyekum MW, Kyei-Gyamfi S. "You cannot stay with one person once you begin having sex at a young age": the prevalence, correlates and effects of early sexual debut among children in Ghana. Reprod Health [Internet]. 2024;21(1):1–10. Available from: https://doi.org/10.1186/s12978-024-01775-4

8. Pringle J, Mills KL, McAteer J, Jepson R, Hogg E, Anand N, et al. The physiology of adolescent sexual behaviour: A systematic review. Cogent Soc Sci. 2017;3(1):1–14.

9. Kyilleh JM, Tabong PTN, Konlaan BB. Adolescents' reproductive health knowledge, choices and factors affecting reproductive health choices: A qualitative study in the West Gonja District in Northern region, Ghana. BMC Int Health Hum Rights. 2018;18(1):1–12.

10. WHO. Adolescent health. World Heal Organ. 2020;83–108.

11. Wondimagegne YA, Debelew GT, Koricha ZB. Barriers to contraceptive use among secondary school adolescents in Gedeo zone, South Ethiopia: A formative qualitative study. BMJ Open. 2023;13(3):1–10.

12. Masonbrink AR, Hurley EA, Schuetz N, Rodean J, Rupe E, Lewis K, et al. Sexual behaviors, contraception use and barriers among adolescents and young adults in rural Haiti. BMC Womens Health. 2023;23(1):1–10.

13. Dandala S, Hodambia M. Journal Wetenskap Health. J Wet Heal [Internet]. 2021;4(4):22–7. Available from: https://pdfs.semanticscholar.org/dc01/0a49f45e1d6d020306d577378e24a2bd59ea.pdf

14. James PB, Osborne A, Babawo LS, Bah AJ, Margao EK. The use of condoms and other birth control methods among sexually active school-going adolescents in nine sub-Saharan African countries. BMC Public Health [Internet]. 2022;22(1):1–11. Available from: https://doi.org/10.1186/s12889-022-14855-6

15. Devi P. Sexual and Reproductive Health Behaviour of Adolescents-A Survey article. 2016;III(10):10918–29. Available from: www.euacademic.org

16. Etrawati F, Yeni Y, Lionita W, Rahmawaty A, Fajarningtiyas DN. Sexual Risk Behavior and its Impact on Unwanted Pregnancy Among Adolescents. J Ilmu Kesehat Masy. 2024;14(3):319–21.

17. Agbanyo Ri. KNOWLEDGE AND BARRIERS TO CONTRACEPTIVE USE AMONG IN-SCHHOL ADOLESCENT IN ADAKLU DISTRICT. 2018;(10637685):1–74.

18. Amoah EJ, Hinneh T, Aklie R. Determinants and prevalence of modern contraceptive use among sexually active female youth in the Berekum East Municipality, Ghana. PLoS One [Internet]. 2023;18(6 JUNE):1–18. Available from: http://dx.doi.org/10.1371/journal.pone.0286585

19. Beson P, Appiah R, Adomah-Afari A. Modern contraceptive use among reproductive-aged women in Ghana: Prevalence, predictors, and policy implications. BMC Womens Health. 2018;18(1):1–8.

20. Alidou S, Dahourou LD, Dah TTE, Sogo A, Kenao TS, Yehadji D, et al. Unmet needs for modern contraceptive methods among sexually active adolescents and young women in Togo: a nationwide cross-sectional study. Front Public Heal. 2023;11(July).

21. Atiglo DY, Biney AAE. Correlates of sexual inactivity and met need for contraceptives among young women in Ghana. BMC Womens Health. 2018;18(1):1–10.

22. Mahama E, Owusu-agyei S. Use of contraceptives among adolescents in Kintampo , Ghana : a cross-sectional study. 2014;7–15.

23. WHO. Contraception Evidence brief. 2019;(3).

24. Agyemang J, Newton S, Nkrumah I, Tsoka-Gwegweni JM, Cumber SN. Contraceptive

use and associated factors among sexually active female adolescents in Atwima Kwanwoma District, Ashanti region-Ghana. Pan Afr Med J. 2019;32:1–13.

25. Sumankuuro J, Asuuri A, Mikare MD, Ngmenkpieo F, Crockett J, Wulifan JK. High School Student's Knowledge, Attitude and Participation in Sexual Health Education in Rural Northern Ghana. J Biosci Med. 2020;08(05):64–83.

26. Keogh SC, Otupiri E, Castillo PW, Li NW, Apenkwa J, Polis CB. Contraceptive and abortion practices of young Ghanaian women aged 15–24: evidence from a nationally representative survey. Reprod Health [Internet]. 2021;18(1):1–17. Available from: https://doi.org/10.1186/s12978-021-01189-6

27. Oppong FB, Logo DD, Agbedra SY, Adomah AA, Amenyaglo S, Arhin- K, et al. Determinants of contraceptive use among sexually active unmarried adolescent girls and young women aged 15 – 24 years in Ghana : a nationally representative cross- - sectional study. 2021;1–10.

28. Li Z, Patton G, Sabet F, Zhou Z, Subramanian S V., Lu C. Contraceptive Use in Adolescent Girls and Adult Women in Low- And Middle-Income Countries. JAMA Netw Open. 2020;3(2):1–15.

29. Muhlisa M, Amiruddin R, Moedjiono AI, Suriah S, Hadju V, Salmah U, et al. Risky reproductive health behavior and information access on adolescents: Mix-method analysis. Sci Eng Heal Stud. 2024;18:1–6.

30. Anyomi MA, Adjei JS, Anyomi BK. Knowledge , attitudes , and perceptions of contraceptive use among senior high school students in Hohoe , Ghana : Addressing

barriers to adolescent sexual health . Knowledge , attitudes , and perceptions of contraceptive use among senior high school stude. 2025;(December 2024).

31. Lahole BK, Banga D, Mare KU. Modern contraceptive utilization among women of reproductive age in Ghana: a multilevel mixed-effect logistic regression model. Contracept Reprod Med. 2024;9(1).

32. Osborne A, Aboagye RG, Bangura C, Ahinkorah BO. Predictors of intention to use contraceptives among married and cohabiting women in Ghana: A cross-sectional study. Contracept Reprod Med. 2024;9(1).

33. Ministry of Health. 2021 Holistic Assessment Report. Minist Heal [Internet]. 2022;(April):56. Available from: https://www.moh.gov.gh/wp-content/uploads/2022/09/2021-Holistic-Assessment-Report_v1.7.3.pdf

34. Nkenguye W, Ismail H, Urassa EP, Yongolo NM, Kagoye S, Msuya SE. Factors Associated with Modern Contraceptive Use Among Out of School Adolescent Girls in Majengo and Njoro Wards of Moshi Municipality, Tanzania. East African Heal Res J. 2023;7(1):32–9.

35. Michael TO, Ojo TF, Ijabadeniyi OA, Ibikunle MA, Oni JO, Agboola AA. Prevalence and factors associated with contraceptive use among sexually active adolescent girls in 25 sub-Saharan African countries. PLoS One [Internet]. 2024;19(2 February):1–15. Available from: http://dx.doi.org/10.1371/journal.pone.0297411

36. Upadhyay UD, Raifman S, Raine-Bennett T. Effects of relationship context on contraceptive use among young women. Contraception [Internet]. 2016;94(1):68–73.

Available from: http://dx.doi.org/10.1016/j.contraception.2016.02.025

37. Wang X, Cheng Z. Cross-Sectional Studies: Strengths, Weaknesses, and Recommendations. Chest [Internet]. 2020;158(1):S65–71. Available from: https://doi.org/10.1016/j.chest.2020.03.012

38. Ghana Statistical Service. Ghana 2021 Population and Housing Census. 2021;6.

39. Bhargava M, Bhargava A, Ghate SD, Shyama Prasad Rao R. Nutritional status of Indian adolescents (15-19 years) from National Family Health Surveys 3 and 4: Revised estimates using WHO 2007 Growth reference. PLoS One [Internet]. 2020;15(6 June):1–24. Available from: http://dx.doi.org/10.1371/journal.pone.0234570

40. Slovin's. Slovin formula in research pdf. 1960;

41. Krishna R, Maithreyi R, Surapaneni KM. Research bias: A review for medical students. J Clin Diagnostic Res. 2010;4(2):2320–4.

42. Attibu RIE. Sexual Behaviour among Adolescents in Senior High Schools within the Tamale Metropolis, Northern Region of Ghana. 2015;(10268216):1–98.

43. Mauti G, Muthoni S. Early Sexual Debut and the Associated Factors among School Going Adolescents in Selected Schools in Kenya. East African Heal Res J. 2023;7(2):309–17.

44. Vincent G, Alemu FM. Factors contributing to, and effects of, teenage pregnancy in Juba. South Sudan Med J. 2016;9(2):28–31.

45. Kara WSK, Benedicto M, Mao J. Knowledge, Attitude, and Practice of Contraception

Formatted: Finnish

Methods Among Female Undergraduates in Dodoma, Tanzania. Cureus. 2019;11(4).

46. Hagan JE, Buxton C. Contraceptive Knowledge, Perceptions and Use among Adolescents in Selected Senior High Schools in the Central Region of Ghana. J Sociol Res. 2012;3(2):170–80.

47. Khumalo S, Taylor M, Makusha T, Mabaso M. Intersectionality of cultural norms and sexual behaviours: a qualitative study of young Black male students at a university in KwaZulu-Natal, South Africa. Reprod Health. 2020;17(1):1–10.

48. Maness SB, Thompson EL, Lu Y. Associations Between Social Determinants of Health and Adolescent Contraceptive Use: An Analysis From the National Survey of Family Growth. Fam Community Heal. 2022;45(2):91–102.

49. Kinaro J, Kimani M, Ikamari L, Ayiemba EHO. Perceptions and Barriers to Contraceptive Use among Adolescents Aged 15 - 19 Years in Kenya: A Case Study of Nairobi. Health (Irvine Calif). 2015;07(01):85–97.

50. Beksinska M, Wong R, Smit J. Male and female condoms: Their key role in pregnancy and STI/HIV prevention. Best Pract Res Clin Obstet Gynaecol. 2020;66:55–67.

51. Chandra-Mouli V, Akwara E, Engel D, Plessons M, Asnake M, Mehra S, et al. Progress in adolescent sexual and reproductive health and rights globally between 1990 and 2016: what progress has been made, what contributed to this, and what are the implications for the future? Sex Reprod Heal Matters [Internet]. 2020;28(1). Available from: https://doi.org/10.1080/26410397.2020.1741495

52. Sam NB. Knowledge and Awareness Level of Contraceptive Usage Among Adolescents

in Mankranso of Ahafo-Ano South District. Biomed J Sci Tech Res. 2020;31(4).

53. Chernick S L, Orav EJ, Brennan TA, Burstin HR. in an Emergency Department. J Gen Intern Med. 1999;14(3):82–7.

54. Abdul I, Imohagene A, Nigeria LO… S of, 2018 undefined. Awareness, knowledge and contraceptive use among female students in. JesonOrgNg [Internet]. 2018;1(2). Available from: https://jeson.org.ng/index.php/jeson/article/view/17

55. James PB, Osborne A, Babawo LS, Bah AJ, Margao EK. The use of condoms and other birth control methods among sexually active school-going adolescents in nine sub-Saharan African countries. BMC Public Health. 2022;22(1):1–13.

56. Cruzeiro ALS, Souza LD de M, Silva RA da, Pinheiro RT, Rocha CLA da, Horta BL. Comportamento sexual de risco: fatores associados ao número de parceiros sexuais e ao uso de preservativo em adolescentes. Cien Saude Colet. 2010;15(suppl 1):1149–58.

Supporting Information

S-1 File (DOCX)

S-2 File (DOCX)

S- 3 Data (XLS)

S-4 File (DOCX)

S-5 File (DOCX)

REBUTTAL LETTER

ADDITIONAL EDITORS COMMENT	
**ABTRACT**	
**COMMENTS**	**RESPONSE**
The background should indicate the aim or objectives of the study as well as the gap the study is filling.	Thank you for the comment. The manuscript has been revised to include aim of the study and the gap the study is addressing. **(refer to page 2, lines 17-21).**
**COMMENTS**	**RESPONSE**
A sentence in the methods on lines 23-24 should indicate general categories of the independent variables – such as demographic, sexual activity, etc…	Thank you for the comment. We have indicated the general categories of the independent variables to ensure more clarity. **(refer to page 2, lines 25-27).**
**COMMENTS**	**RESPONSE**
A few more additional keywords can be included for the study.	Thank you for the comment. We have revised the manuscript and added three more keywords **(refer to page 3, lines 44).**
**REVIEWERS COMMENT**	
**REVIEWER ONE**	
**COMMENTS**	**RESPONSE**
The author did a great work; however, the abstract should be unstructured per the PLos GPH guidelines.	Thank you for drawing our attention, we have unstructured the abstract to be in line with PLos GPH guidelines (**refer to page 2-3, line 12-42**).

ADDITIONAL EDITORS COMMENT	
**INTRODUCTION**	
**COMMENTS**	**RESPONSE**

The Introduction is Ghana-focused and does not indicate where your study fits in the global discourse on adolescent sexual and reproductive health.	Thank you for the comment. The Introduction has been revised to better situate the study within the global discourse on adolescent sexual and reproductive health. We have added global information and statistics on adolescent births, sexual behaviour, and contraceptive use. **(refer to page 3, lines 47-49; line 52-54; Page 4,5, Lines 77-80)**
**COMMENTS**	**RESPONSE**
The Introduction does not include any discussion of a theory or conceptual framework adopted to support the study. How did the authors select the independent variables that they did?	Thank you for the comment. Although conceptual framework was not originally outlined in the study, the Introduction has been revised to include a discussion on the selection of independent variables. These variables were informed by a review of existing literature, which identified key factors such as age, residential status, employment status, and number of sexual partners as influencing contraceptive use among adolescents. This has now been clearly stated in the operational definition, along with relevant references. **(refer to page 6, lines 115-119).**
**COMMENTS**	**RESPONSE**
The authors should avoid general statements about adolescent sexual behaviour, especially without including references.	Thank you for the insightful comment. General statements about adolescent sexual behaviour have been supported with appropriate references from reviewed literature, such as those from the World Health Organization. For example, statements regarding early sexual initiation, hormonal influences during adolescence, and associated risks of unprotected sex have now been substantiated with relevant citations **(refer to page 3, line 51-52; page 4 lines 67-69).**

COMMENTS	RESPONSE
What does it mean to engage in sexual activity at earlier ages? The authors should specify what early sex entails – Line 60	Thank you for the comment. The manuscript has been revised to define what it means to engage in sexual activity at earlier ages. This refers to adolescents engaging in sexual intercourse before the age of 15, which is generally categorized by the WHO as early adolescents(10-14years) **(refer to page 3, lines 52-54).**
Why is it important to study SHS students? This should be further justified- Line 91	Thank you for the comment. The manuscript has been revised to provide a clearer justification for focusing on Senior High School (SHS) students. We now emphasize that SHS students represent a unique subgroup within the adolescent population marked by heightened curiosity and risk-taking behaviour, including sexual experimentation. However, they often have limited access to reliable sexual and reproductive health information, especially within the formal school curriculum. Targeting this group is therefore crucial for informing school-based interventions and policy reforms aimed at improving adolescent reproductive health outcomes **(refer to page 5, lines 85-88).**

ADDITIONAL EDITORS COMMENT	
**METHODS**	
**COMMENTS**	**RESPONSE**
State the region of Ghana that Ejisu Municipality is in – Line 104	Thank you for the comment. The manuscript has been revised to include the specific region. We have now specified that the study was conducted in Ejisu Municipality, located in the Ashanti Region of Ghana **(refer to page 7, lines 136-137).**

COMMENTS	RESPONSE
Additional justification for choice of conducting a study in only this school is needed	Thank you for the comment. We have expanded the “Study Setting” section to provide additional justification for selecting the school. The school was purposively chosen for its mixed-gender population, diverse socioeconomic representation, large enrollment, range of academic programs, and accessibility. The willingness of the school administration to support the research also contributed to its selection **(refer to page 8, lines 146-151).**
**COMMENTS**	**RESPONSE**
There is no sub-section indicating how variables were measured. This sub-section should include a description on how the attitudes to contraception variable was constructed.	Thank you for the comment. We have added a new sub-section titled “Measurement of Variables.” This includes a detailed explanation of how sexual activity, awareness, contraceptive use, and attitudes toward contraceptives were measured. In particular, we explained how the attitude variable was constructed using a 7-item Likert scale, with reverse coding for negatively worded items and median split for classification into positive and negative attitudes **(refer to page 11-12, lines 221-236).**
**COMMENTS**	**RESPONSE**
An indication of the number of models run by the authors should be made	Thank you for the comment. We have clarified in the “Data Processing and Analysis” section that two models were run.<br><br>**Model 1:** Univariate binary logistic regression to calculate crude odds ratios.<br><br>**Model 2:** Multivariable binary logistic regression adjusted for key sociodemographic variables (age,

	sex, class, religion, and residential status) (**refer to page 13, lines 247-252**).
**COMMENTS**	**RESPONSE**
Are there 10-year-olds in SHS? What was the minimum age? That should be stated instead of 10 years.	Thank you for the comment. While it is uncommon, it is possible for 10-year-olds to be enrolled in Senior High School in Ghana. For example, a recent case in Mfantsipim School involved a 10-year-old SHS2 student (https://www.instagram.com/tv3_ghana/reel/DIIy2qPNLR_/?locale=ja-JP&hl=en). Furthermore, with the WHO definition of adolescence (10–19 years), we retained the original age categorization. However, for clarity, we have now explicitly stated in the "Study Population" section that the actual age range of our participants was 11 to 19 years, and the 10–14 years category was used for analytical consistency with WHO standards (**refer to page 8, lines 154-159**).
**COMMENTS**	**RESPONSE**
Why were SHS 2 and 3 students selected and not SHS 1 students?	Thank you for the comment. We have explained in the "Study Population" section that SHS 1 students were on vacation at the time of data collection and were therefore not available to participate in the study (**refer to page 8, lines 157-159**).
**REVIEWERS COMMENT**	
**REVIEWER ONE**	
**COMMENTS**	**RESPONSE**
In methods, under Quality Assurance, Validity line 204, author mentioned that six public health experts reviewed the structured questionnaires	Thank you for drawing our attention to this inconsistency. The correct number of experts involved in the review was five, not six. This has been corrected in the manuscript for accuracy and consistency (**refer to page 13, line 254)**.

however, in line 207, he mentioned that ‘all five experts.	

ADDITIONAL EDITORS COMMENT	
**RESULTS**	
**COMMENTS**	**RESPONSE**
The authors should ensure that only the results that align with their study objectives are presented.	Thank you for the comment. The manuscript has been revised to present only the results that align with the objective. The section that presented on barriers to contraceptive use has been removed, since it was not primarily part of the objectives.
**COMMENTS**	**RESPONSE**
Table 7 should indicate percentages only in the cells and total numbers can be placed on a column to the right.	Thank you for the comment. Table 7 which is now table 6 has been revised to display only percentages within the cells, and the total numbers have been added in a separate column on the right, as suggested. **(refer to page 28-30, lines 338-340).**
**COMMENTS**	**RESPONSE**
The sample size keeps changing for different frequencies conducted and should be made clear why.	Thank you for the comment. The sample size kept changing because some of the questions were follow-up questions for participants who answered "yes" or "no" to a particular question. For instance, question 8 asked, "Do you have a boyfriend/girlfriend?" and question 9 was a follow-up question: "If yes, what is the age of your boyfriend/girlfriend?" A similar pattern applies to questions 14 and 15, as well as others.
**COMMENTS**	**RESPONSE**

The results indicate barriers to contraceptive use along with other variables which have not been discussed in the introduction. The lack of a conceptual framework discussing the variables being assessed results in this lack of clarity about the independent variables.	Thank you for the comment. We have revised the manuscript to include a discussion of the independent variables being assessed for greater clarity. As previously stated, the results on barriers have been removed**.** All revisions have been made to improve the overall quality of the manuscript **(refer to page 6, lines 99-108)**.
**COMMENTS**	**RESPONSE**
It is important to have an indication of which variables are independent, and which are controls. Why were no control variables included in the regression model.	Thank you for the comment. We have indicated the independent variables in the manuscript under the 'Operational Definition' section and the 'Data Processing and Analysis' section. Previously, we did not include control variables because only the variables found to be significant in the chi-square analysis were included in the model. However, following your comment and further review, we have revised the manuscript to include control variables (age, sex, class, religion, and residential status) (**refer to page 6, lines 115-119**) (**refer to page 13, lines 249 – 253**).
**REVIEWERS COMMENT**	
**REVIEWER ONE**	
**COMMENTS**	**RESPONSE**
Reasons for participants not using any contraceptives method (N=26) but total frequency of the responses was 63.	Thank you for your comment. Yes, 26 participants answered that question, but they were allowed to select more than one response, which accounts for the 63 responses. However, as previously stated, this section has been removed.

Again, the author used pie chart where negative responses to contraceptive use was 23%. 23% of the sample is approximately 76. That section needs to be revised.	Thank you for your comment. This section has been revised. The actual number of those who showed a negative attitude was 77, corresponding to 23.3%. The method used to determine this has been explained in "Attitude Towards Contraceptive Use" section (**refer to page 11-12, lines 225-236**).
Maybe I missed, but I wanted to see proportion of adolescents accepting or using any forms of the family planning methods.	Thank you for your comment. We presented the adolescents who accepted using any form of the family planning method and the methods used in table 4.<br>**(refer to page 21, lines 310-311).**
In the discussion, the author mentioned the Ghana Health Family planning target as 23.3%. with reference to Ghana Health Services Holistic Assessment Indicator tool, the FP acceptor rate target is 40% of the total WIFA population.	Thank you for drawing our attention to this. The manuscript has been revised to capture the actual national family planning target which is 33.8% with reference to Ghana Health Service Holistic Assessment Indicator tool. **(refer to page 35, line 400-401).**
**REVIEWER TWO**	
**COMMENTS**	**RESPONSE**
All the tables presented should be in APA format. Ensure that they are presented in APA format to improve the status of the manuscript.	Thank you for your comment. The manuscript has been improved by presenting all the tables in the ''results'' section in APA format.<br>**(refer to page 14-33, lines 281-356).**

**ADDITIONAL EDITORS COMMENT**
**LIMITATION**

COMMENTS	RESPONSE
The small sample size should be indicated as a limitation	Thank you for the comment. The small sample size of the study has been acknowledged and indicated as a limitation **(refer to page 38, lines 478-479).**
