# Supplementary material for: Sexual activity and contraceptive use among adolescents: A descriptive survey in a Ghanaian municipality
Source: PLOS Glob Public Health. 2025 Aug 25;5(8):e0005039. doi: 10.1371/journal.pgph.0005039 (PMC12377570; doi:10.1371/journal.pgph.0005039)
Supplement: Attachment — Submitted filename: Reviewers Comment.docx [file pgph.0005039.s005.docx]

**Reviewer’s Comment**

**Strengths**

- The manuscript examines sexual activity and contraceptive use among

adolescents in senior high school and the author(s) made a good attempt at it.

- The abstract contains the substance of the study.
- The methodology of the study is sufficient. Thus, adequate data was used, and the authors made use of appropriate sampling technique.
- The instrument used in gathering data was appropriate and well developed.
- The author(s) adequately discussed the protocols followed to uphold ethical standards.
- The authors adopted appropriate technique for analysing the data.
- The findings of the study were also in line with the study’s objectives, which informed the discussion of findings. The discussion was done in light of literature
- Conclusions drawn and recommendations made were based on the findings of the study.
- Appropriate standard used was used in the manuscript.

**Weaknesses**

The author(s) should work on the comments and suggestions made to improve the quality of the study.

- Subheadings should be given to the various headings in the work to make it easy for readers to follow and comprehend, particularly in the results section. For example;

1. Introduction
2. Operational definitions
3. Materials and methods
   3.1 Study setting
   3.2 Study design
   3.3 Study population
   3.4 Sample size determination and sampling
   3.5 Sampling technique
   3.6 Inclusion and exclusion criteria
   3.7 Data collection tool
   3.8 Data collection procedure
   3.9 Data processing and analysis
   3.10 Data validity
   3.11 Ethical considerations
4. Results
   4.1 Socio-demographic characteristics of respondents
5. Discussion
6. Conclusion and recommendation
7. Limitations of the study
8. Acknowledgement
9. References

- Few typographical and grammatical errors were identified in the manuscript. Work on those comments as suggested by the reviewer.
- All the tables presented should be in APA format. Ensure that they are presented in APA format to make improve the status of the manuscript.
- Work on all the other comments made in the text as suggested.
