# Supplementary material for: Sexual activity and contraceptive use among adolescents: A descriptive survey in a Ghanaian municipality
Source: PLOS Glob Public Health. 2025 Aug 25;5(8):e0005039. doi: 10.1371/journal.pgph.0005039 (PMC12377570; doi:10.1371/journal.pgph.0005039)
Supplement: Attachment — Submitted filename: Response to Reviewers.docx [file pgph.0005039.s006.docx]

**REBUTTAL LETTER**

| **ADDITIONAL EDITORS COMMENT** | |
| --- | --- |
| **ABTRACT** | |
| **COMMENTS** | **RESPONSE** |
| The background should indicate the aim or objectives of the study as well as the gap the study is filling. | Thank you for the comment. The manuscript has been revised to include aim of the study and the gap the study is addressing. **(refer to page 2, lines 17-21).** |
| **COMMENTS** | **RESPONSE** |
| A sentence in the methods on lines 23-24 should indicate general categories of the independent variables – such as demographic, sexual activity, etc… | Thank you for the comment. We have indicated the general categories of the independent variables to ensure more clarity. **(refer to page 2, lines 25-27).** |
| **COMMENTS** | **RESPONSE** |
| A few more additional keywords can be included for the study. | Thank you for the comment. We have revised the manuscript and added three more keywords **(refer to page 3, lines 44).** |
| **REVIEWERS COMMENT** | |
| **REVIEWER ONE** | |
| **COMMENTS** | **RESPONSE** |
| The author did a great work; however, the abstract should be unstructured per the PLos GPH guidelines. | Thank you for drawing our attention, we have unstructured the abstract to be in line with PLos GPH guidelines (**refer to page 2-3, line 12-42)**. |

| **ADDITIONAL EDITORS COMMENT** | |
| --- | --- |
| **INTRODUCTION** | |
| **COMMENTS** | **RESPONSE** |
| The Introduction is Ghana-focused and does not indicate where your study fits in the global discourse on adolescent sexual and reproductive health. | Thank you for the comment. The Introduction has been revised to better situate the study within the global discourse on adolescent sexual and reproductive health. We have added global information and statistics on adolescent births, sexual behaviour, and contraceptive use. **(refer to page 3, lines 47-49; line 52-54; Page 4,5, Lines 77-80)** |
| **COMMENTS** | **RESPONSE** |
| The Introduction does not include any discussion of a theory or conceptual framework adopted to support the study. How did the authors select the independent variables that they did? | Thank you for the comment. Although conceptual framework was not originally outlined in the study, the Introduction has been revised to include a discussion on the selection of independent variables. These variables were informed by a review of existing literature, which identified key factors such as age, residential status, employment status, and number of sexual partners as influencing contraceptive use among adolescents. This has now been clearly stated in the operational definition, along with relevant references. **(refer to page 6, lines 115-119).** |
| **COMMENTS** | **RESPONSE** |
| The authors should avoid general statements about adolescent sexual behaviour, especially without including references. | Thank you for the insightful comment. General statements about adolescent sexual behaviour have been supported with appropriate references from reviewed literature, such as those from the World Health Organization. For example, statements regarding early sexual initiation, hormonal influences during adolescence, and associated risks of unprotected sex have now been substantiated with relevant citations **(refer to page 3, line 51-52; page 4 lines 67-69).** |
| **COMMENTS** | **RESPONSE** |
| What does it mean to engage in sexual activity at earlier ages? The authors should specify what early sex entails – Line 60 | Thank you for the comment. The manuscript has been revised to define what it means to engage in sexual activity at earlier ages. This refers to adolescents engaging in sexual intercourse before the age of 15, which is generally categorized by the WHO as early adolescents(10-14years) **(refer to page 3, lines 52-54).** |
| Why is it important to study SHS students? This should be further justified- Line 91 | Thank you for the comment. The manuscript has been revised to provide a clearer justification for focusing on Senior High School (SHS) students. We now emphasize that SHS students represent a unique subgroup within the adolescent population marked by heightened curiosity and risk-taking behaviour, including sexual experimentation. However, they often have limited access to reliable sexual and reproductive health information, especially within the formal school curriculum. Targeting this group is therefore crucial for informing school-based interventions and policy reforms aimed at improving adolescent reproductive health outcomes **(refer to page 5, lines 85-88).** |

| **ADDITIONAL EDITORS COMMENT** | |
| --- | --- |
| **METHODS** | |
| **COMMENTS** | **RESPONSE** |
| State the region of Ghana that Ejisu Municipality is in – Line 104 | Thank you for the comment. The manuscript has been revised to include the specific region. We have now specified that the study was conducted in Ejisu Municipality, located in the Ashanti Region of Ghana **(refer to page 7, lines 136-137).** |
| **COMMENTS** | **RESPONSE** |
| Additional justification for choice of conducting a study in only this school is needed | Thank you for the comment. We have expanded the “Study Setting” section to provide additional justification for selecting the school. The school was purposively chosen for its mixed-gender population, diverse socioeconomic representation, large enrollment, range of academic programs, and accessibility. The willingness of the school administration to support the research also contributed to its selection **(refer to page 8, lines 146-151).** |
| **COMMENTS** | **RESPONSE** |
| There is no sub-section indicating how variables were measured. This sub-section should include a description on how the attitudes to contraception variable was constructed. | Thank you for the comment. We have added a new sub-section titled “Measurement of Variables.” This includes a detailed explanation of how sexual activity, awareness, contraceptive use, and attitudes toward contraceptives were measured. In particular, we explained how the attitude variable was constructed using a 7-item Likert scale, with reverse coding for negatively worded items and median split for classification into positive and negative attitudes **(refer to page 11-12, lines 221-236).** |
| **COMMENTS** | **RESPONSE** |
| An indication of the number of models run by the authors should be made | Thank you for the comment. We have clarified in the “Data Processing and Analysis” section that two models were run.  **Model 1:** Univariate binary logistic regression to calculate crude odds ratios.  **Model 2:** Multivariable binary logistic regression adjusted for key sociodemographic variables (age, sex, class, religion, and residential status) (**refer to page 13, lines 247-252**). |
| **COMMENTS** | **RESPONSE** |
| Are there 10-year-olds in SHS? What was the minimum age? That should be stated instead of 10 years. | Thank you for the comment. While it is uncommon, it is possible for 10-year-olds to be enrolled in Senior High School in Ghana. For example, a recent case in Mfantsipim School involved a 10-year-old SHS2 student (https://www.instagram.com/tv3_ghana/reel/DIIy2qPNLR_/?locale=ja-JP&hl=en). Furthermore, with the WHO definition of adolescence (10–19 years), we retained the original age categorization. However, for clarity, we have now explicitly stated in the “Study Population” section that the **actual age range** of our participants was **11 to 19 years**, and the 10–14 years category was used for analytical consistency with WHO standards (**refer to page 8, lines 154-159**). |
| **COMMENTS** | **RESPONSE** |
| Why were SHS 2 and 3 students selected and not SHS 1 students? | Thank you for the comment. We have explained in the “Study Population” section that SHS 1 students were on vacation at the time of data collection and were therefore not available to participate in the study (**refer to page 8, lines 157-159**). |
| **REVIEWERS COMMENT** | |
| **REVIEWER ONE** | |
| **COMMENTS** | **RESPONSE** |
| In methods, under Quality Assurance, Validity line 204, author mentioned that six public health experts reviewed the structured questionnaires however, in line 207, he mentioned that ‘all five experts. | Thank you for drawing our attention to this inconsistency. The correct number of experts involved in the review was **five,** not six. This has been corrected in the manuscript for accuracy and consistency (**refer to page 13, line 254)**. |

| **ADDITIONAL EDITORS COMMENT** | |
| --- | --- |
| **RESULTS** | |
| **COMMENTS** | **RESPONSE** |
| The authors should ensure that only the results that align with their study objectives are presented. | Thank you for the comment. The manuscript has been revised to present only the results that align with the objective. The section that presented on barriers to contraceptive use has been removed, since it was not primarily part of the objectives. |
| **COMMENTS** | **RESPONSE** |
| Table 7 should indicate percentages only in the cells and total numbers can be placed on a column to the right. | Thank you for the comment. Table 7 which is now table 6 has been revised to display only percentages within the cells, and the total numbers have been added in a separate column on the right, as suggested. **(refer to page 28-30, lines 338-340).** |
| **COMMENTS** | **RESPONSE** |
| The sample size keeps changing for different frequencies conducted and should be made clear why. | Thank you for the comment. The sample size kept changing because some of the questions were follow-up questions for participants who answered "yes" or "no" to a particular question. For instance, question 8 asked, "Do you have a boyfriend/girlfriend?" and question 9 was a follow-up question: "If yes, what is the age of your boyfriend/girlfriend?" A similar pattern applies to questions 14 and 15, as well as others. |
| **COMMENTS** | **RESPONSE** |
| The results indicate barriers to contraceptive use along with other variables which have not been discussed in the introduction. The lack of a conceptual framework discussing the variables being assessed results in this lack of clarity about the independent variables. | Thank you for the comment. We have revised the manuscript to include a discussion of the independent variables being assessed for greater clarity. As previously stated, the results on barriers have been removed**.** All revisions have been made to improve the overall quality of the manuscript **(refer to page 6, lines 99-108)**. |
| **COMMENTS** | **RESPONSE** |
| It is important to have an indication of which variables are independent, and which are controls. Why were no control variables included in the regression model. | Thank you for the comment. We have indicated the independent variables in the manuscript under the 'Operational Definition' section and the 'Data Processing and Analysis' section. Previously, we did not include control variables because only the variables found to be significant in the chi-square analysis were included in the model. However, following your comment and further review, we have revised the manuscript to include control variables (age, sex, class, religion, and residential status) (**refer to page 6, lines 115-119**) (**refer to page 13, lines 249 – 253**). |
| **REVIEWERS COMMENT** | |
| **REVIEWER ONE** | |
| **COMMENTS** | **RESPONSE** |
| Reasons for participants not using any contraceptives method (N=26) but total frequency of the responses was 63. | Thank you for your comment. Yes, 26 participants answered that question, but they were allowed to select more than one response, which accounts for the 63 responses. However, as previously stated, this section has been removed. |
| Again, the author used pie chart where negative responses to contraceptive use was 23%. 23% of the sample is approximately 76. That section needs to be revised. | Thank you for your comment. This section has been revised. The actual number of those who showed a negative attitude was 77, corresponding to 23.3%. The method used to determine this has been explained in "Attitude Towards Contraceptive Use" section (**refer to page 11-12, lines 225-236**). |
| Maybe I missed, but I wanted to see proportion of adolescents accepting or using any forms of the family planning methods. | Thank you for your comment. We presented the adolescents who accepted using any form of the family planning method and the methods used in table 4.  **(refer to page 21, lines 310-311).** |
| In the discussion, the author mentioned the Ghana Health Family planning target as 23.3%. with reference to Ghana Health Services Holistic Assessment Indicator tool, the FP acceptor rate target is 40% of the total WIFA population. | Thank you for drawing our attention to this. The manuscript has been revised to capture the actual national family planning target which is 33.8% with reference to Ghana Health Service Holistic Assessment Indicator tool. **(refer to page 35, line 400-401).** |
| **REVIEWER TWO** | |
| **COMMENTS** | **RESPONSE** |
| All the tables presented should be in APA format. Ensure that they are presented in APA format to improve the status of the manuscript. | Thank you for your comment. The manuscript has been improved by presenting all the tables in the ‘’results’’ section in APA format.  **(refer to page 14-33, lines 281-356).** |

| **ADDITIONAL EDITORS COMMENT** | |
| --- | --- |
| **LIMITATION** | |
| **COMMENTS** | **RESPONSE** |
| The small sample size should be indicated as a limitation | Thank you for the comment. The small sample size of the study has been acknowledged and indicated as a limitation **(refer to page 38, lines 478-479).** |
