# Supplementary material for: Sexual activity and contraceptive use among adolescents: A descriptive survey in a Ghanaian municipality
Source: PLOS Glob Public Health. 2025 Aug 25;5(8):e0005039. doi: 10.1371/journal.pgph.0005039 (PMC12377570; doi:10.1371/journal.pgph.0005039)
Supplement: Attachment — Submitted filename: Response_to_Reviewers_auresp_2.docx [file pgph.0005039.s008.docx]

| **ADDITIONAL EDITORS COMMENT** | |
| --- | --- |
|  | |
| **COMMENTS** | **RESPONSE** |
| lines 15 - 17 - revise the sentence since a heightened sexual drive and hormonal changes in adolescents does not lead to the low contraceptive use | Thank you for this insightful comment. We have revised the sentence in order to ensure clarity. This revision decouples the causal link previously implied between hormonal changes and low contraceptive use.  **(refer to page 2, lines 15 - 18).** |
| **COMMENTS** | **RESPONSE** |
| line 109 - indicate why studying adolescents in school, and particularly in senior high schools is important | Thank you for the comment. In response, we have revised line 109 to clearly highlight the importance of studying adolescents in senior high schools. This revision highlights the significance of the school environment as a key context in adolescent development, where attitudes and behaviors related to sexual and reproductive health are shaped. It also emphasizes that senior high school students represent a pivotal group for targeted interventions and policy development.  **(refer to page 6, lines 108-114).** |
| **COMMENTS** | **RESPONSE** |
| Table 1 – state as ‘living with both parents’ not ‘both parent’; regarding the living alone category, are they living with other relatives/guardians or are they truly living alone. | Thank you for the comment. The manuscript has been revised to conform with the suggestions made. Also, the category of ‘living alone’ pertains to those who truly stays alone.  **(refer to page 15 - 16, lines 293 - 294).** |
| **COMMENTS** | **RESPONSE** |
| Table 3 – consistency with the ‘N’ is needed by each variable. Some included the N and others do not. | Thank you for drawing our attention to this inconsistency. The manuscript has been revised and all variables in table 3 do has the N.  **(refer to page 19 - 23, lines 312 - 313).** |
| **COMMENTS** | **RESPONSE** |
| A major issue lies in the inclusion of non-sexually active adolescents in the bivariate and multivariate analysis where use of modern contraception is the dependent variable. Either they are removed for these analyses, or the study focusses on the dependent variable such as attitudes where both sexually active and non-sexually active respondents can be included. | Thank you for bringing such a major issue to our attention. The manuscript has been revised. All cases of non-sexually active adolescents have been filtered out in the inferential statistics and the authors have successfully re-run both the bivariate and multivariate analysis. These changes have been reflected in the reporting of the results, tables of all inferential statistics **(refer to page 29-34, lines 343 - 377)** and also a section of the discussion **(refer to page 38, lines 460 - 471).** |
| **COMMENTS** | **RESPONSE** |
| The authors must read through the entire manuscript and address all grammatical and typographical errors | Thank you for the comment. We have addressed all grammatical and typographical errors. |
| **COMMENTS** | **RESPONSE** |
| Note that some of the supporting documents attached include identifying information about the senior high school that was studied | Thank you for bringing this to our attention. In response to this, we have removed the document labeled S-5, which was the approval letter from the school containing their identifying information. |
| **COMMENTS** | **RESPONSE** |
| Include a paragraph or two providing some contextual information about the setting within which the school is situated - particularly the region or district. | Thank you for the comment. The study setting section have been expanded to include contextual details about the Ejisu Municipality, such as its geographical location, demographic characteristics, and educational infrastructure. This provides readers with a clearer understanding of the study environment **(refer to page 7, lines 122-130).** |
| **COMMENTS** | **RESPONSE** |
| The Operational Definitions section should be included in the Materials and Methods section. | Thank you for the comment. The Operational Definitions have been relocated to Section 3.3 of the Materials and Methods as requested **(refer to page 8-9, lines 145-160).** |
| **COMMENTS** | **RESPONSE** |
| Lines 152 - write out the dates - 16th August 2022 and 15th September 2022. | Thank you for the comment. We have revised the text to include the full date formats: '16th August 2022' and '15th September 2022'.  **(refer to page 9, lines 160).** |
| **COMMENTS** | **RESPONSE** |
| In the data collection tool sub-section - the reference to number of questions as items e.g. 7 items, 8 items, is inaccurate since they are questions. The only accurate reference would be on line 194 to the 7 Likert scale items. | Thank you for the comment. All references to numbered questions have been corrected to reflect them as 'questions' instead of 'items', except for the 7 Likert scale items which remain accurately labeled.  (**refer to page 10-11, lines 183, 186, 191, and 194**). |
| **COMMENTS** | **RESPONSE** |
| Measurement of variables section should include a description of all the variables and how they are measured - both independent and dependent variables. | Thank you for the comment. We have revised Section 3.8 (Measurement of Variables) to include detailed descriptions of both the dependent and independent variables and the formats used to measure them. (**refer to page 12-13, lines 231 – 255**). |
| **COMMENTS** | **RESPONSE** |
| Lines 253 to 258 - regarding the quality assurance, validity section - When was this process done? If after the tool was developed, then move it closer to that section. Placing this after the data analysis section suggests the tool was validated after the analysis. | Thank you for the comment. We have relocated the validity section (now 3.8) to immediately follow the Data Collection Tool section (**refer to page 11, lines 207 – 215**). |
| **COMMENTS** | **RESPONSE** |
| An additional assessment of the scale items for 'attitude toward contraception' where the Cronbach alpha value is computed could be useful for assessing reliability of the variable.” | Thank you for this suggestion. In response, we have now reported the calculated Cronbach’s alpha based on the pretest sample, which yielded a coefficient of 0.707 for the scale items measuring adolescents’ attitudes toward contraceptive use. This indicates that the items demonstrated acceptable reliability for assessing the construct.  (**refer to page 11, lines 213 – 215**). |

|  | **REVIEWERS COMMENT** | |
| --- | --- | --- |
|  | **CONCLUSION AND RECOMMENDATION** | |
| **COMMENTS** | | **RESPONSE** |
| Consider adding a brief section on programmatic implications for school-based sexual education programs based on your findings. | | Thank you for the comment. We have revised the manuscript to include adolescent-responsive sexual and reproductive health programs in school settings. This section emphasizes the importance of comprehensive sexuality education that addresses not only biological aspects but also values, consent, and communication skills. We further highlight the role of peer-led initiatives and youth-friendly approaches to ensure programs are contextually relevant and effective **(refer to page 40, lines 492 - 496).** |
| **COMMENTS** | | **RESPONSE** |
| A short paragraph acknowledging the evolving nature of adolescent sexual behaviors in light of technological and social media influences might strengthen the contemporary relevance. | | Thank you for the comment. To reflect the contemporary realities influencing adolescent sexual behavior, we have added a paragraph discussing the impact of technological and social media influences. We also propose leveraging digital platforms such as interactive apps, social media, and online counseling as tools to deliver accurate, age-appropriate information and counter misinformation. **(refer to page 39, lines 481 - 484).** |

| **JOURNAL REQUIREMENTS** | |
| --- | --- |
|  | |
| **COMMENTS** | **RESPONSE** |
| Please review your reference list o ensure that it is complete and correct. If you have cited papers that have been retracted, please include the rationale for doing so in the manuscript text, or remove these references and replace them with relevant current references. Any changes to the reference list should be mentioned in the rebuttal letter that accompanies your revised manuscript. If you need to cite a retracted article, indicate the articles retracted status in the reference list and also included a citation and full reference for the retracted notice. | Thank you for the comment. The authors have reviewed the reference list and none of the references have been retracted. Moreover, the authors have made changes to the reference list, as some of the previously cited articles have been removed and new articles have been added when we were revising the manuscript. These changes were made to address all the comments from the Editor and the reviewers in order to improve the manuscript for publication. |
